# Amino Functionality Enables Aqueous Synthesis of Carboxylic Acid-Based MOFs at Room Temperature by Biomimetic Crystallization

**DOI:** 10.1021/acs.inorgchem.4c00245

**Published:** 2024-05-14

**Authors:** Xiangyu Wang, Samarth Pratap Singh, Tongtong Zhang, Rebecca Andrews, Maria Giovanna Lizio, George F. S. Whitehead, Imogen A. Riddell

**Affiliations:** Department of Chemistry, University of Manchester, Oxford Road, Manchester M13 9PL, United Kingdom

## Abstract

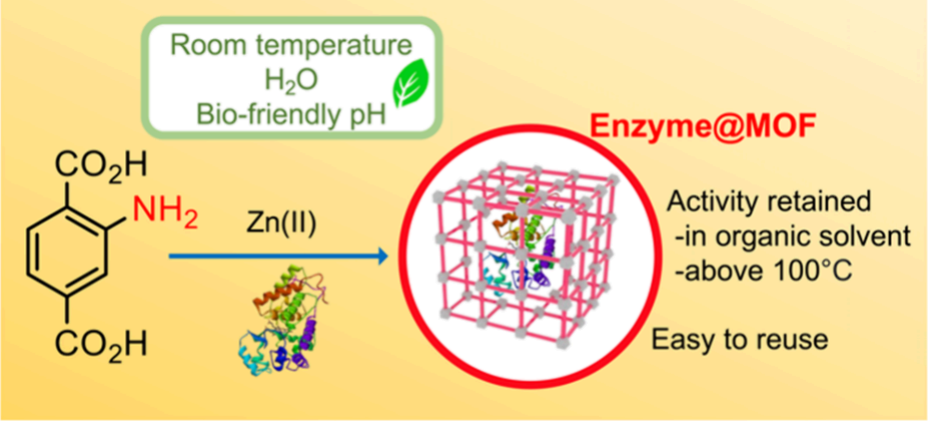

Enzyme immobilization within metal–organic frameworks (MOFs) is a promising solution to avoid denaturation and thereby utilize the desirable properties of enzymes outside of their native environments. The biomimetic mineralization strategy employs biomacromolecules as nucleation agents to promote the crystallization of MOFs in water at room temperature, thus overcoming pore size limitations presented by traditional postassembly encapsulation. Most biomimetic crystallization studies reported to date have employed zeolitic imidazole frameworks (ZIFs). Herein, we expand the library of MOFs suitable for biomimetic mineralization to include zinc(II) MOFs incorporating functionalized terephthalic acid linkers and study the catalytic performance of the enzyme@MOFs. Amine functionalization of terephthalic acids is shown to accelerate the formation of crystalline MOFs enabling new enzyme@MOFs to be synthesized. The structure and morphology of the enzyme@MOFs were characterized by PXRD, FTIR, and SEM-EDX, and the catalytic potential was evaluated. Increasing the linker length while retaining the amino moiety gave rise to a family of linkers; however, MOFs generated with the 2,2′-aminoterephthalic acid linker displayed the best catalytic performance. Our data also illustrate that the pH of the reaction mixture affects the crystal structure of the MOF and that this structural transformation impacts the catalytic performance of the enzyme@MOF.

## Abstract 2

Inclusion of the amine functional group within terephthalic acid linkers enables aqueous, room temperature synthesis of MOFs incorporating catalytically active enzymes. The MOF structure is determined by the pH of the biomimetic crystallization reaction and has a consequent effect on the catalytic behavior. Here we demonstrate that a 2D layered MOF encapsulates HRP, and the enzyme@MOF shows activity in organic solvent and at 100 °C. The enzyme@MOF particles also enable straightforward enzyme recycling.

## Introduction

Enzymes are an important class of biomacromolecules that catalyze chemical transformations with a high degree of selectivity and efficiency and thus have long been regarded as exciting biotechnological prospects.^1^ Their application within industrial processes is however limited by the fragile tertiary structure of enzymes which can readily be disrupted by high temperature, organic solvents, acidic or basic pH, and degradative proteases.^2^ Indeed, using enzymes homogeneously without a suitable stabilization mechanism tends to reduce the catalytic potential of enzymes, resulting in low reuse efficiency and related cost implications. In order to prevent enzyme denaturation and activity loss, enzyme immobilization on the surface or within solid materials has been trialled with promising results.^3^

Metal–organic frameworks (MOFs) have undergone a combinatorial explosion since their initial emergence as a class of material.^4^ Self-assembled from organic linkers and inorganic metal ions or clusters, MOFs have a modular porous structure, making them an ideal choice for enzyme immobilization. Specifically, MOFs can act as a constrictive framework that can inhibit peptide unfolding and aggregation while the large specific surface area supports high enzyme loading.^5^ Furthermore, the tunable pore size and functionality can enable highly selective uptake of the substrate, which can further enhance the catalytic activity of enzymes.^2,5a,6,7^

Enzyme–MOF composites can be synthesized by a variety of methods including physical adsorption^8^ and chemical bonding.^9^ As physical adsorption relies on weak interactions between enzyme molecules and organic linkers, this can lead to enzyme leaching from MOFs, resulting in low immobilization efficiency and reusability. Additionally, MOF pore sizes restrict physical adsorption methods to biomolecules of smaller sizes.^6c,10^ Chemical bonding has also been shown as a route to successfully immobilize enzymes, but it frequently exhibits drawbacks, particularly loss of enzymatic activity as a result of structural damage.^11^ In contrast, facile biomimetic mineralization^12^ overcomes pore size limitations by employing mild self-assembly conditions suitable for the inclusion of native enzymes.^13^ A range of biomolecules, including enzymes, have been reported to nucleate MOF formation under conditions distinctly different from those employed for conventional MOF synthesis. To date, however, a limited number of MOFs have been reported to form using biomimetic crystallization, with ZIFs (zeolitic imidazolate frameworks) making up the majority of the reported examples.^12,14^ Expanding the library of MOFs that can be generated by biomimetic mineralization would provide access to materials with different internal properties (including size, shape, and electrostatics) and thus enable optimization of enzyme encapsulation and conformation as well as substrate uptake and product egress. Recently several new MOFs, which are not ZIFs but are well established to form via solvothermal methods, have been reported following biomimetic mineralization including UiO-66, IRMOFs, and MILs.^15^ Among these, MOFs generated from carboxylic acid-based linkers, including terephthalic acid (BDC), [1,1′-biphenyl]-4,4′-dicarboxylic acid (BPDC), benzene-1,3,5-tricarboxylic acid (BTC), and their derivatives, are promising for use in enzyme immobilization via biomimetic mineralization^12,16^ but suffer from shortcomings including long reaction times, isolation of amorphous precipitation, and the requirement for heavy metal ions with poor biocompatibility. In contrast, we demonstrate rapid, biomimetic crystallization occurs with zinc(II) and amino-functionalized carboxylate ligands: 2-aminobenzene-1,4-dicarboxylic acid (BDC-NH_2_), 3,3′-diamino-[1,1′-biphenyl]-4,4′-dicarboxylic acid (BPDC-NH_2_), and 3,3″-diamino-[1,1′:4′,1″-terphenyl]-4,4″-dicarboxylic acid (TPDC-NH_2_). Deprotonation of the carboxylic acid groups is required to dissolve these ligands in water (a prerequisite for biomimetic crystallization); however, once dissolved MOF formation proceeds spontaneously in the presence of zinc(II) nitrate, and studies demonstrate that encapsulated horseradish peroxidase (HRP) maintains its catalytic activity.

## Results and Discussion

### Biomimetic Mineralization of Functionalized ZnBDC MOFs

Preliminary studies set out to evaluate the suitability of functionalized terephthalic acid linkers for use in the one-pot biomimetic crystallization of MOFs in the presence of bovine serum albumin (BSA). Negatively charged moieties on the surface of BSA have previously been reported to promote ZIF-8 nucleation;^17^ we hypothesized that electrostatic interactions between BSA and zinc(II) would promote nucleation of MOFs with terephthalic acid linkers in a comparable fashion. The effect of the functional group (−H, −NH_2_, −Me, −OH, and −Br) on the terephthalic acid linkers was unknown. Few systematic studies investigating the preparation of MOFs by biomimetic crystallization have been reported,^16c^ and in particular, studies investigating the variation of functional groups on one family of ligands employed for biomineralization reactions have yet to be reported.

**Figure 1:**
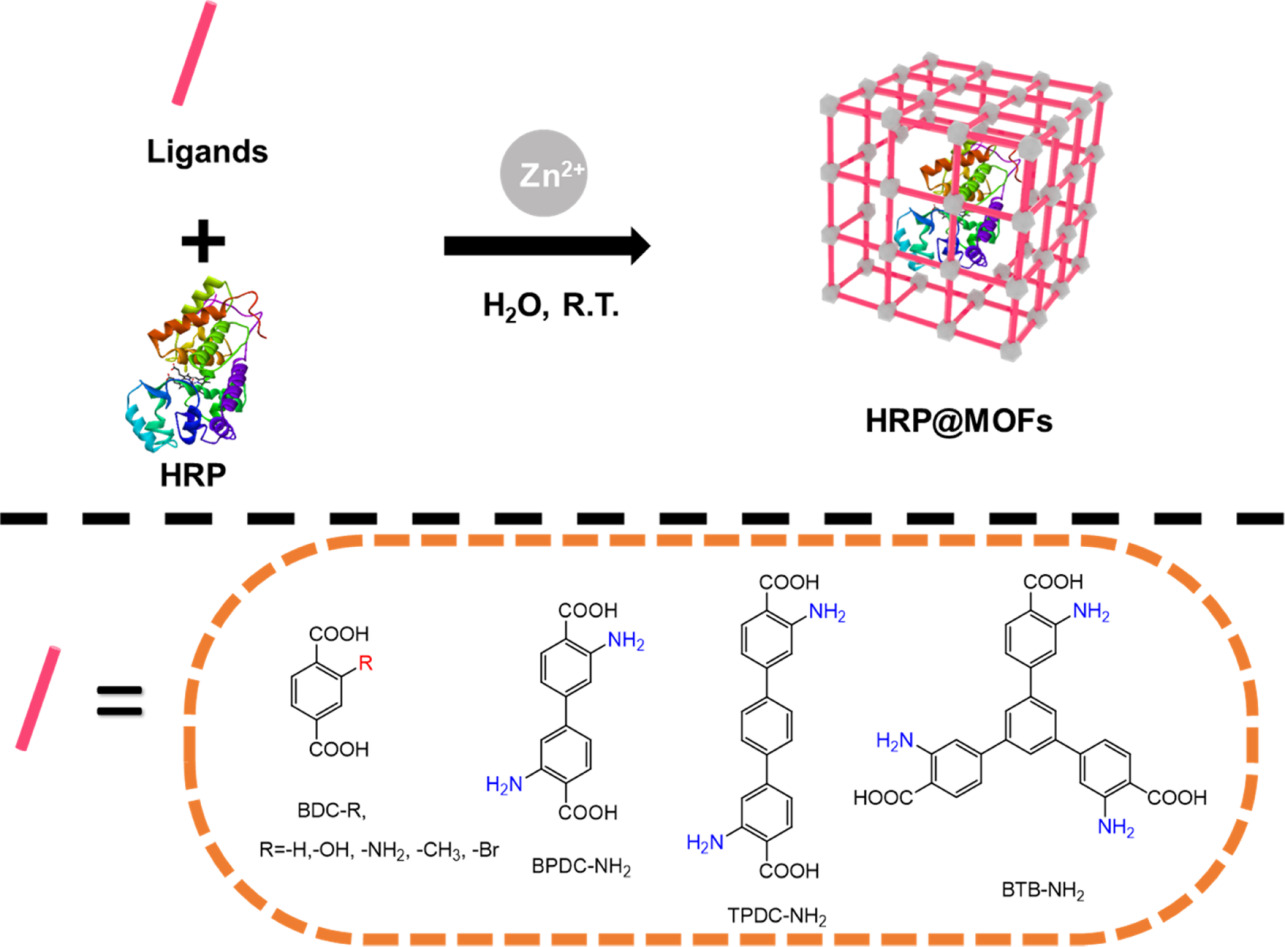
(a, top) Biomimetic mineralization of HRP@ZnBDC-NH_2_ via self-assembly of ligand and zinc(II) ions in aqueous conditions at room temperature in the presence of a protein (HRP). (b, bottom) Amino-functionalized organic ligands employed in this study.

Each of these five dicarboxylic ligands (Figure 1) was deprotonated using sodium hydroxide before being mixed with BSA at 0.25 mg/mL to prepare a ligand solution with a final concentration of 25 mM. This solution was then added to a separate solution of zinc(II) nitrate (25 mM) under rapid stirring. Any solid precipitates were collected and analyzed. For linker BDC-NH_2_ which incorporates an amino group ortho to one of the carboxylic acid moieties, the rate of precipitate formation was significantly enhanced over that of precipitate formation with the parent terephthalic acid. Moreover, BDC-NH_2_ yielded a greater mass of precipitate (∼150 mg) than the other linkers investigated, the precipitate formation was complete within 30 min, and powder X-ray diffraction (PXRD) analysis of the precipitate supported the formation of a highly crystalline material. In contrast, the parent terephthalic acid linker necessitated at least 36 h for the crystallization to complete (Table S1) and yielded negligible product under reaction conditions analogous to those employed with the BDC-NH_2_ linker. These results are consistent with prior research indicating the rapid precipitation of Fe(III) and Al(IV) MOFs^16e,16f^ with BDC-NH_2_ during biomimetic mineralization.

### Amino-Functionalized Carboxylic Acids as Precursors for Biomimetically Crystallized MOFs

**Figure 2:**
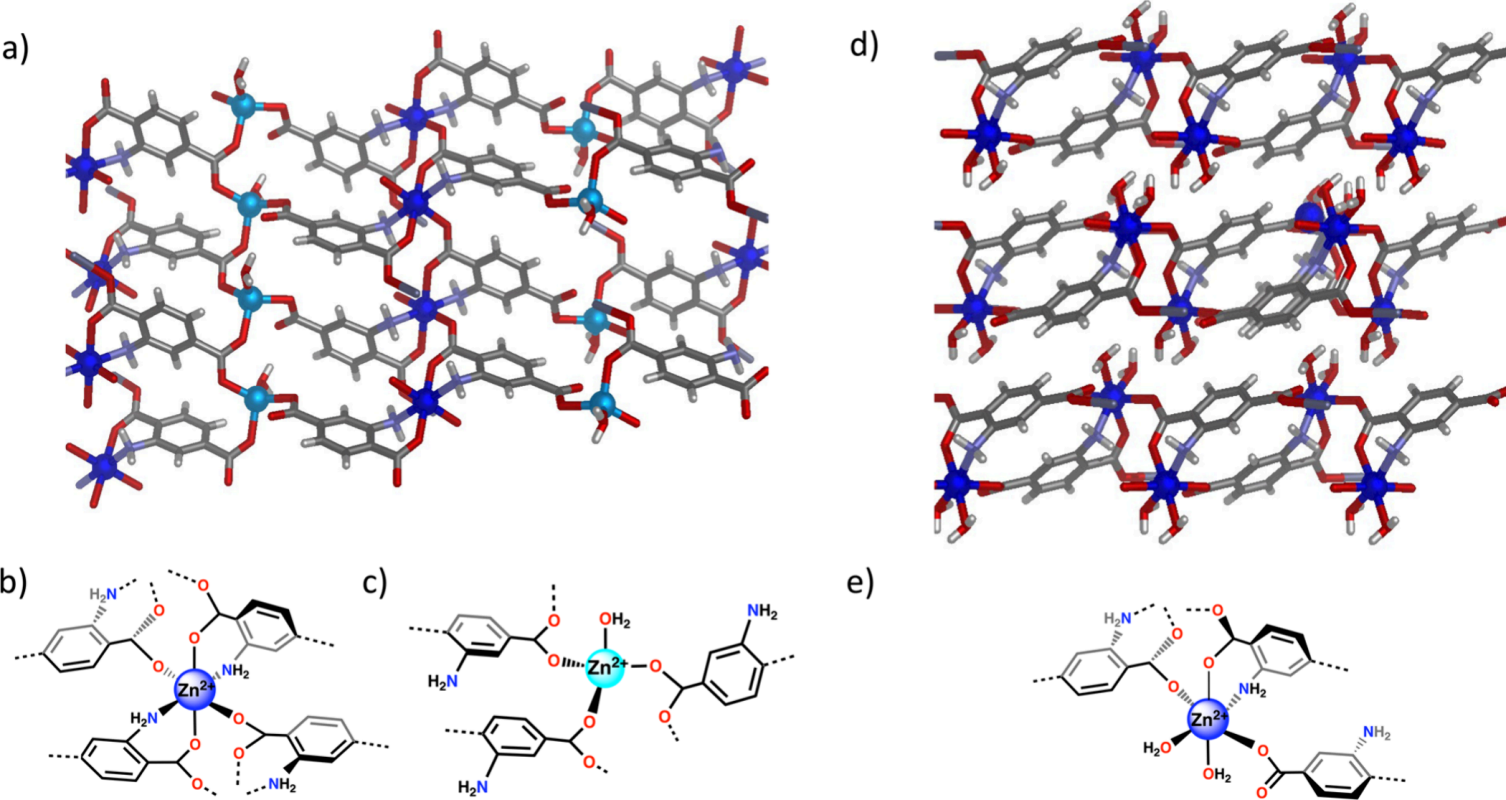
(a) Structure of BSA@ZnBDC-NH_2_ I generated when biomimetic crystallization is performed at pH < 5.3. BSA@ZnBDC-NH_2_ I incorporated alternating sequences of octahedrally coordinated zinc(II) ions (dark blue, (b)) and tetrahedral zinc(II) ions (light blue, (c)). (d) Structure of BSA@ZnBDC-NH_2_ II, a 2D layered MOF which incorporates zinc within a single octahedral environment (e). Figure generated from reported PXRD patterns (CCDC IDs 1943053 and 607821).^18,19^

Having identified that the amino functionality uniquely gave rise to high yields of the crystalline product via biomimetic crystallization, we set out to determine if the inclusion of this moiety within other carboxylic acid linkers provided a general route to the formation of crystalline materials capable of encapsulating proteins. A family of linkers were synthesized (BDC-NH_2_, BPDC-NH_2_, TPDC-NH_2_, and BTB-NH_2_) and characterized (Supporting Information Section S1). The products of their aqueous reactions with proteins and zinc(II) nitrate were then analyzed by PXRD, Fourier-transform infrared spectroscopy (FTIR), and scanning electron microscopy with energy-dispersive X-ray spectroscopy (SEM-EDX) (Section S3). PXRD studies of the product generated from 2-aminobenzenedicarboxylic acid (BDC-NH_2_) revealed that two distinct products were formed. The pH of the reaction mixture was shown to determine the final product, with the two distinct polymorphs identified termed ZnBDC-NH_2_ I and ZnBDC-NH_2_ II (Figures 2a and 2d). Polymorph I (BSA@ZnBDC-NH_2_ I)^18^ precipitated exclusively when the pH of the solution was below 5.3. When the pH of the reaction mixture was above 5.3, the structure of BSA@ZnBDC-NH_2_ tended toward polymorph II^19^ (Figure S41). The PXRD pattern of the BSA@ZnBDC-NH_2_ I, generated under weakly acidic conditions, was consistent with a previously reported MOF structure in which both the carboxylate and the amino groups directly coordinate with the zinc(II) metal centers.^18^ Close examination of the structure reveals two distinct zinc(II) coordination environments: one zinc(II) exists in a four-coordinate tetrahedral geometry where three binding sites are occupied by three dicarboxylic acid linkers and the fourth site is filled by a water molecule, and the second zinc(II) ion exhibits six-coordinate, octahedral geometry with four carboxylate oxygens, from four carboxylate groups and two amino groups bound directly to the metal center (Figures 2b and 2c). In this configuration all of the amino groups on the ligand are bound to metal sites and are therefore unavailable for interaction with guest molecules. Moreover, there is negligible void volume within this structure, which presents a limitation for substrate and product diffusion when considering the catalytic applications proposed for these materials (Figure S44).

Characterization of the second polymorph revealed a 2D layered MOF in which each zinc(II) ion is octahedrally coordinated to one amine group, three carboxylate groups, and two water molecules. The water ligands and the uncoordinated carbonyl moiety are directed into the interlayer space and able to form hydrogen bonds with complementary partners in the adjacent layer.^19^ This PXRD pattern was previously recorded^20^ when MOF46^21^ particles were stirred in water and the DMF solvent molecules, which traditionally cap the zinc paddlewheels in MOF46, were removed from the complex. Polymorph II may therefore interact with residues on the surface of encapsulated proteins via multiple hydrogen bonding opportunities as well as direct coordination to Lewis acidic zinc(II) ions, which may be generated by displacement of the two water ligands. Despite conserved 1:1 Zn:L stoichiometry in polymorphs I and II, two different structures can be cleanly formed when the pH of the reaction mixture is controlled.

**Figure 3:**
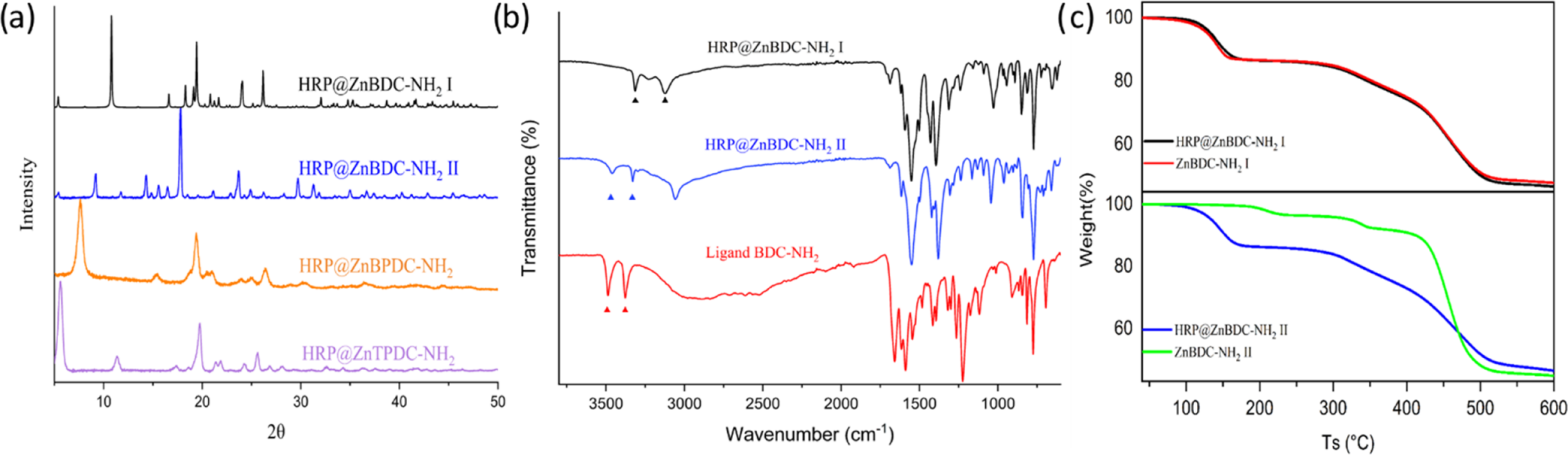
(a) PXRD pattern of HRP@ZnBDC-NH_2_ I (black), HRP@ZnBDC-NH_2_ II (blue), HRP@ZnBPDC-NH_2_ (orange), and HRP@ZnTPDC-NH_2_ (lilac)_._ (b) FTIR spectrum of HRP@ZnBDC-NH_2_ I (black) and II blue. Ligand spectrum shown in red and position of NH_2_ stretching frequencies marked with triangles. (c) TGA curves of HRP@ZnBDC-NH_2_ I (upper) and II (lower) in the presence and absence of HRP.

After the structures and synthetic conditions required to form BSA@ZnBDC-NH_2_ I and II were identified, BSA was replaced in the biomimetic crystallization procedure with the oxidative enzyme horseradish peroxidase (HRP). The range of carboxylic acid linkers was also expanded to include amino-functionalized linkers BPDC-NH_2_, TPDC-NH_2_, and BTB-NH_2_, which vary in length and the number of amino moieties. Increasing the ligand length from BDC (7.58 Å) to BPDC-NH_2_ (13.82 Å) and TPDC-NH_2_ (18.04 Å), by increasing the number of aromatic rings in the ligand backbone, is reported to generate isoreticular structures under traditional solvothermal syntheses conditions.^22^ Similarly, when the extended ligands were employed for biomimetic crystallization reactions, the PXRD spectra were consistent with the formation of isoreticular structures (Figure 3a). In contrast, reactions with the 3-fold symmetric ligand BTB-NH_2_ did not yield a successful outcome. Despite extensive attempts, the BTB-NH_2_ ligand could not be fully solubilized in water, even in the presence of excess sodium hydroxide, and therefore could not be considered for application in biomimetic crystallization reactions.

Examination of the FTIR spectra of the HRP@ZnBDC-NH_2_ polymorphs I and II (Figure 3b) and their deuterated analogues supported the presence of coordinated water molecules in both MOF networks (observed at 3123 and 3057 cm^–1^, respectively). The asymmetric and symmetric NH_2_ stretching frequencies for polymorphs I and II (Figure 3b, marked with triangles) supported different amine coordination environments. In the 1400–1600 cm^–1^ region of the IR spectra, multiple degenerate carboxylate and NH bending frequencies are observed, consistent with carboxylates coordinated in both mono- and bidentate orientations. The infrared spectra of HRP@MOFs with longer ligands share common features with the FTIR spectra of HRP@ZnBDC-NH_2_ I. No clear peaks corresponding to HRP could be observed in any of the IR spectra due to the large number of overlapping MOF peaks at frequencies where HRP peaks may be observed. Similarly, Raman spectra of HRP@ZnBDC-NH_2_ I and II also failed to provide direct evidence of HRP encapsulation due to the low loading content of the samples and potential signal degeneracy between the HRP peaks and those of the MOF (Figure S47).

**Figure 4:**
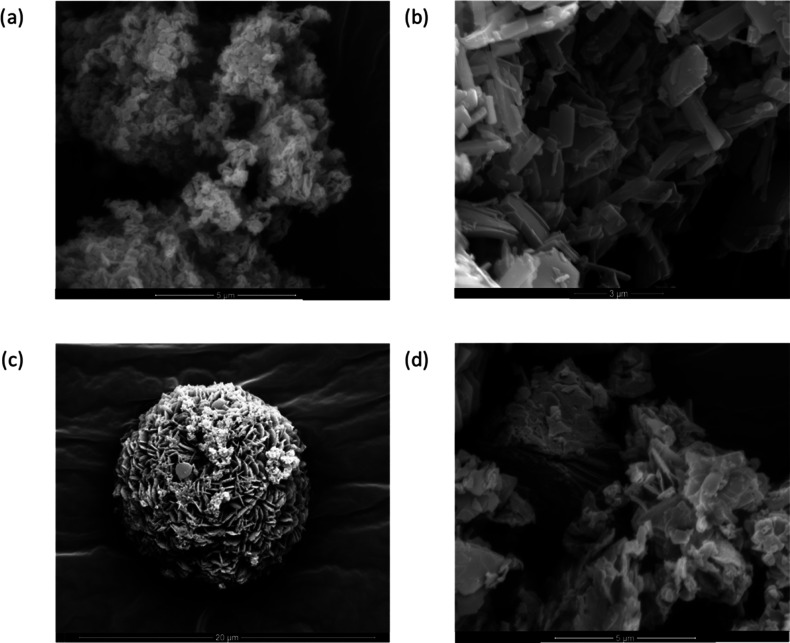
SEM images of (a) HRP@ZnBDC-NH_2_ I, (b) HRP@ZnBDC-NH_2_ II, (c) HRP@ZnBDC-NH_2_ II collected after 48 h of mixing (spherical structure with a diameter of 12 μm), and (d) ZnBDC-NH_2_ II synthesized in the absence of HRP.

The morphology of HRP@ZnBDC-NH_2_ I and II was analyzed next (Figures 4a and 4b). SEM images of HRP@ZnBDC-NH_2_ I showed large composites consisting of small clusters of plates with an average length of 300 nm. Crystals of HRP@ZnBDC-NH_2_ II were initially observed to be more block-like with average dimensions of 1 × 0.5 μm^2^; however, acquisition of SEM images following different experimental durations indicated once formed the crystals underwent self-assembly to generate hollow spherical structures (Figures 4c and S52). Once fully formed, the spheres have an average diameter of 12 μm and are hollow in the center. This hierarchical assembly of crystals into spheres has previously been reported.^20^ On the contrary, no aggregation was observed for HRP@ZnBDC-NH_2_ I particles even after extended experimental durations. SEM studies also showed that ZnBDC-NH_2_ I and II prepared in the absence of protein exhibited a different lamellar crystal form (Figures 3d and S51) to the structures observed with HRP.

HRP@ZnBPDC-NH_2_ and HRP@ZnTPDC-NH_2_ were also characterized by SEM, with the HRP@ZnBPDC-NH_2_ crystals observed to be roughly spherical, while HRP@ZnTPDC-NH_2_ presented as irregular plates lacking a uniform particle size (Figures S55 and S59). The hydrodynamic diameters of HRP@ZnBPDC-NH_2_ and HRP@ZnTPDC-NH_2_ were measured by dynamic light scattering (DLS); the particles of HRP@ZnBPDC-NH_2_ had an average hydrodynamic diameter of 355 nm, while the HRP@ZnTPDC-NH_2_ particles had a slightly smaller average hydrodynamic diameter of 200 nm (Table S11).

Support for the inclusion of HRP within HRP@MOFs was obtained through elemental and thermogravimetric analysis (TGA) of crystalline materials alongside analysis of the reaction supernatant after the MOF assembly. Since HRP is an iron enzyme, the percentage mass of iron in the crystals was measured by SEM-EDX and ICP-OES. All measurements confirmed low percentage weight inclusion of iron (between 0.1 and 0.5%), regardless of the sample or analysis technique employed.

The encapsulation efficiency (EE%) (eq S1) of HRP and the HRP loading content (LC%) (eq S2) in HRP@MOFs were calculated using the Bradford assay to quantify the amount of unreacted HRP in solution following MOF precipitation. An ideal encapsulation matrix would have a high EE%, reflecting almost complete incorporation of the enzyme, and a high LC%, whereby the ratio of the encapsulation matrix is minimized relative to the active enzyme. For MOFs generated from ZnBDC-NH_2_ little difference in the encapsulation efficiency of HRP was observed between HRP@ZnBDC-NH_2_ I and HRP@ZnBDC-NH_2_ II. For HRP@ZnBDC-NH_2_ I, the EE% and LC% were found to take up 55.6% and 1.5%, respectively, while the EE% and LC% for HRP@ZnBDC-NH_2_ II were calculated as 63.7% and 6.1%, respectively. In contrast, HRP@ZnBPDC-NH_2_ gave a higher encapsulation efficiency of HRP (86.1%), but with a low loading content (1.03%), and HRP@ZnTPDC-NH_2_ exhibited low EE% (24.5%) and LC% (0.06%). Thermogravimetric analysis provided further support for the inclusion of HRP in the HRP@ZnBDC-NH_2_ MOFs (Figure 3c). Decomposition of the ZnBDC-NH_2_ materials was divided into three distinct steps in the TGA spectrum which were attributed to loss of water, loss of ligand and enzyme when present, and finally decomposition of the framework. Little difference in mass loss was observed between the TGA curves of HRP@ZnBDC-NH_2_ I and ZnBDC-NH_2_ I with a difference in mass loss reported for the second step of only 1.5%. The small mass difference coincides with the low loading content (1.5%) of HRP in HRP@ZnBDC-NH_2_ I (Table S12). In contrast, a larger mass loss difference (5.7%) was observed in the second step when comparing HRP@ZnBDC-NH_2_ II and ZnBDC-NH_2_ II; this we attribute to decomposition of HRP, which is present at a higher loading content of 6.1% versus 1.5% observed for polymorph I. The difference in the weight loss between HRP@ZnBDC-NH_2_ II and ZnBDC-NH_2_ II roughly corresponds to the loading content of HRP in HRP@ZnBDC-NH_2_ II (Table S12). HRP incorporation within our MOFs compares favorably with previous reports for HRP incorporation within CuBDC (EE 21%),^6c^ ZnBDC (LC ∼ 1–2%),^16c^ and ZrBDC (LC 1.6%)^23^ MOFs.

Finally, the capacity of these MOFs to immobilize enzymes was supported by confocal microscopy studies employing FITC-BSA as a representative fluorescently tagged protein. FITC-BSA@ZnBDC-NH_2_ I, FITC-BSA@ZnBDC-NH_2_ II, FITC-BSA@ZnBPDC-NH_2_, and FITC-BSA@ZnTPDC-NH_2_ were synthesized by biomimetic mineralization by direct replacement of FITC-BSA in place of HRP (Figure S48) during sample preparation. All FITC-BSA@MOF samples were then analyzed by PXRD and were confirmed to have the expected crystalline structures before confocal imaging studies were undertaken. Each imaged sample demonstrated green fluorescence, supporting the incorporation of FITC-BSA within the MOF particles.

### Catalytic Activity of HRP Incorporating MOFs

Following isolation by precipitation, all HRP@MOF particles were able to be resuspended in aqueous solutions to generate stable colloidal mixtures with no significant precipitation observed over the course of 2 h. The catalytic properties of HRP@ZnBDC-NH_2_ I and II, HRP@ZnBPDC-NH_2_, and HRP@ZnTPDC-NH_2_ could thus reliably be studied using the widely employed solution phase *o*-phenylenediamine/2,3-diaminophenazine (OPD/DAP) colorimetric assay (Scheme S4).^24^

Table 1 contains the key experimental parameters related to the four new constructs as well as pertinent parameters for HRP. HRP@ZnBDC-NH_2_ I and II clearly perform best among the novel materials, while HRP@ZnBPDC-NH_2_ and HRP@ZnTPDC-NH_2_ perform significantly less well, demonstrating both poor substrate binding specificity (*K*_m_) and a 2 orders of magnitude difference in the rate of catalysis (*k*_cat_).

**Table 1:** Michaelis–Menton Parameters (*K*_m_, *V*_max_, *k*_cat_, and *k*_cat_/*K*_m_) Describing Oxidation of OPD to DAP in the Presence of H_2_O_2_ and Free HRP, HRP@ZnBDC-NH_2_ I, HRP@ZnBDC-NH_2_ II, HRP@ZnBPDC-NH_2_, or HRP@ZnTPDC-NH_2_^a^

catalyst	*K*_m_ (μM)	*V*_max_ (μM s^–1^)	*k*_cat_ (s^–1^)	*k*_cat_/*K*_m_ (s^–1^ μM^–1^)
free HRP	10.9 ± 0.4	(1.3 ± 0.065) × 10^–3^	(1.7 ± 0.077) × 10^–1^	(1.4 ± 0.13) × 10^–2^
HRP@ZnBDC-NH_2_ I	452.6 ± 20.9	(3.9 ± 0.17) × 10^–2^	(8.5 ± 0.37) × 10^–1^	(1.9 ± 0.18) × 10^–3^
HRP@ZnBDC-NH_2_ II	104.5 ± 44.2	(5.5 ± 0.51) × 10^–2^	1.2 ± 0.1	(1.1 ± 0.97) × 10^–2^
HRP@ZnBPDC-NH_2_	1086.2 ± 243.8	(6.1 ± 1.1) × 10^–4^	(7.8 ± 0.14) × 10^–2^	(7.2 ± 0.55) × 10^–5^
HRP@ZnTPDC-NH_2_	1134.9 ± 358.4	(8.3 ± 0.89) × 10^–5^	(4.9 ± 0.53) × 10^–2^	(4.3 ± 0.15) × 10^–5^

a For all reactions, the working concentration of HRP was standardized to 50 ng/mL.

Comparison of HRP@ZnBDC-NH_2_ I and II indicates that HRP@ZnBDC-NH_2_ II has both a higher substrate affinity and a higher rate of reaction (*V*_max_ and *k*_cat_) than HRP@ZnBDC-NH_2_ I and is therefore the best performing novel material among those investigated. These results can be rationalized when considering the structures of the HRP@ZnBDC-NH_2_ polymorphs. In particular, HRP@ZnBDC-NH_2_ I is a tightly packed structure with limited opportunities for substrate access and product egress. In contrast, the layered structure of HRP@ZnBDC-NH_2_ II will allow for the easier flow of substrates and products between the layers of the structure. In comparison with free HRP, the catalytic activity of all the HRP@MOFs is reduced except for HRP@ZnBDC-NH_2_ II; this we attribute to either diffusion-limited events that are well-known to impact this type of material or partial deactivation of the enzyme.^17^ Both BDC-NH_2_ polymorphs exhibit competitive *k*_cat_/*K*_m_ values compared with free HRP. We hypothesize that the best performing material, HRP@ZnBDC-NH_2_ II, benefits from the increased polarity of the microenvironment inside the MOF which may aid the diffusion rate of substrates into the MOF and activate the substrates for catalysis.^25^ Control studies confirmed that no catalytic activity was exhibited by the free MOF particles formed in the absence of HRP.

**Figure 5:**
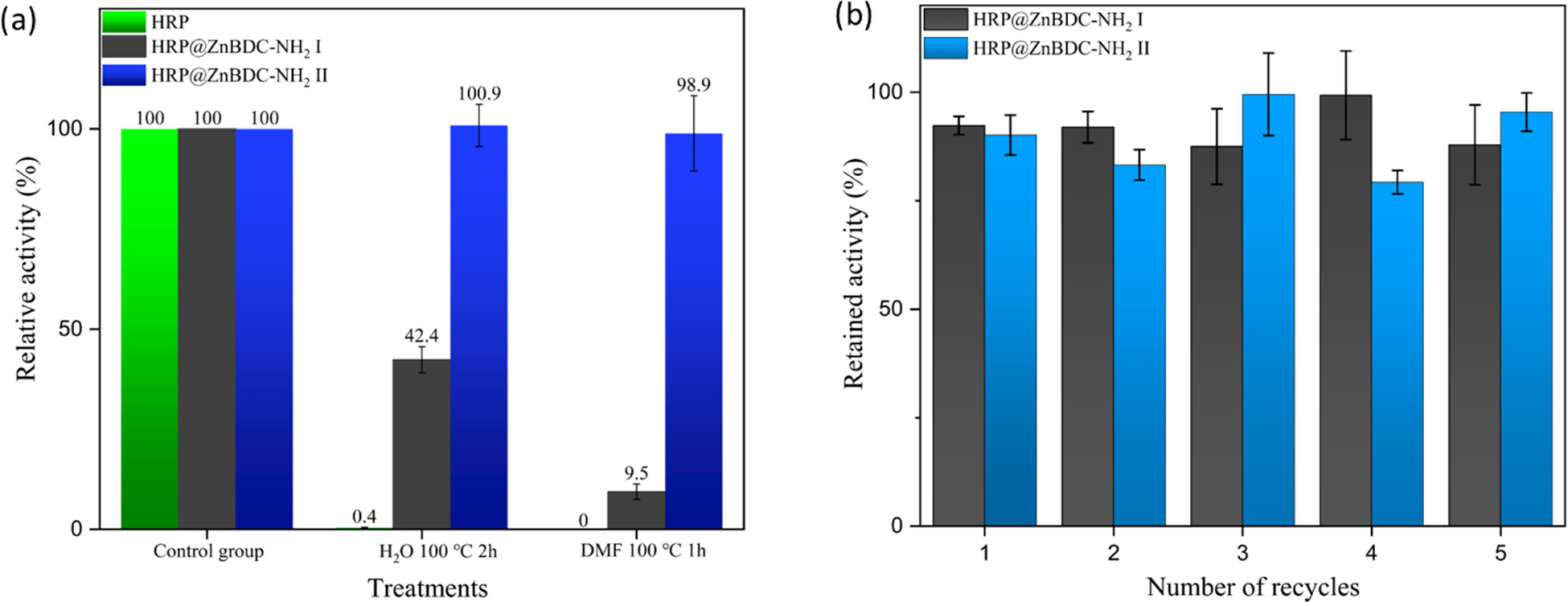
Benefits of the framework for (a) enzyme protection following heating in H_2_O at 100 °C for 2 h and DMF at 100 °C for 1 h and (b) enzyme recycling.

While the HRP@ZnBDC-NH_2_ MOFs did not outperform free HRP under ambient conditions, we were interested in exploring the protective potential of the frameworks and the benefit the framework might provide in terms of enzyme recyclability. Following treatment of HRP, HRP@ZnBDC-NH_2_ I, and HRP@ZnBDC-NH_2_ II with boiling water for 2 h or DMF at 100 °C for 1 h, HRP@ZnBDC-NH_2_ II maintained almost all of its catalytic activity, while HRP@ZnBDC-NH_2_ I retained 42.4% and 9.5% activity, respectively. In contrast, free HRP was almost completely deactivated under these conditions (Figure 5a). PXRD spectra collected following exposure to thermal treatment in water and DMF indicated that both polymorphs of HRP@ZnBDC-NH_2_ maintained their crystal structure after treatment, with only a slight decrease in the peak intensity being observed for HRP@ZnBDC-NH_2_ II following treatment with DMF. This further supports the outstanding thermal stability of ZnBDC-NH_2_ I and II and the potential of this class of material to protect enzymes from deactivation in hot aqueous or organic solvents (Figure S66).

In addition to heat deactivation, the ease of recycling is an issue when considering the application of enzymes within industrial settings. If enzymes are not immobilized during the reaction, separation of the substrate and product following the reaction is difficult and presents challenges for reuse of the enzyme. The reusability of HRP@ZnBDC-NH_2_ I and II was demonstrated by recycling and repeating the colorimetric assay five times. During the course of these reactions, both HRP@ZnBDC-NH_2_ I and II maintained a high relative activity above 80% (Figure 5b). Minor losses in activity are attributed to the inevitable loss of HRP@ZnBDC-NH_2_ during centrifugation and the separation of the particles from the supernatant during each recycling step. Our results align well with literature reports of other enzyme@MOFs which typically report reasonable^16d^ but often reduced kinetics parameters upon enzyme encapsulation,^23^ with the major advantage of immobilization being increased enzyme stability.

## Conclusion

In summary, amino group modification at the ortho position of terephthalic acid and its derivatives was shown to promote the rate of biomimetic mineralization with zinc(II) and HRP and BSA proteins. In contrast to carboxylate ligands lacking amino groups, amino-functionalized ligands gave rise to well-defined crystalline products, which were characterized by PXRD, SEM, TGA, and FTIR spectrometry. This study represents the first systematic investigation of organic linkers for use in biomimetic crystallization, and the new family of HRP@MOFs including the extended BPDC-NH_2_ and TPDC-NH_2_ linkers demonstrates for the first time that extended organic ligands can be used in biomimetic mineralization reactions and demonstrate isoreticular behavior as exhibited in classical solvothermal syntheses. We also identified during the course of our studies that pH is a critical determinant of the biomineralization reaction, demonstrating that two distinct phases are formed with BDC-NH_2_ linkers. Although our HRP@ZnBDC-NH_2_ MOFs exhibited lower activity when compared to free HRP, the metal–organic frameworks do provide benefits to the encapsulated enzymes, increasing their stability under conditions which denature the native enzyme as well as enabling recycling of the HRP. In line with previous reports of MOF biomimetic crystallization,^6b^ we expect our approach to be broadly applicable for encapsulation of a range of biomolecules. Future studies will look to further expand the range of ligands that give rise to well-defined crystalline MOFs with suitable properties to protect industrially viable enzymes and enable their recycling. These studies will present new exciting opportunities for the transformation of highly efficient and selective natural catalysts into industrial processes.

## Supporting Information Available

^1^H NMR, ^13^C NMR, and mass spectra of organic ligands, PXRD patterns, confocal microscopy images, SEM and EDX maps, Raman and FTIR spectra, TGA curves and kinetics data (PDF)
