# Supplementary material for: Amino Functionality Enables Aqueous Synthesis of Carboxylic Acid-Based MOFs at Room Temperature by Biomimetic Crystallization
Source: Inorg Chem. 2024 May 14;63(21):9801–8. doi: 10.1021/acs.inorgchem.4c00245 (PMC11134488; doi:10.1021/acs.inorgchem.4c00245)
Supplement: Supplementary file 1 — ic4c00245_si_001.pdf [file ic4c00245_si_001.pdf]

Supporting Information for

Amino Functionality Enables Aqueous Synthesis of Carboxylic Acid-Based MOFs at Room Temperature by Biomimetic Crystallisation

Xiangyu Wang, Samarth P. Singh, Tongtong Zhang, Rebecca Andrews, Maria Giovanna Lizio, George F. S. Whitehead and Imogen A. Riddell*

Department of Chemistry, University of Manchester, Manchester, M13 9PL, United Kingdom

*Email: imogen.riddell@manchester.ac.uk

Table of Contents

S1 Ligand Synthesis and Characterisation ... 3

S1.1 Materials and Methods ... 3

S1.2 Synthesis of 3,3'-diamino-[1,1'-biphenyl]-4,4'-dicarboxylic acid (BPDC-NH_2) ... 3

S1.3 Synthesis of 3,3''-diamino-5'-(3-amino-4-carboxyphenyl)-[1,1':3',1''-terphenyl]-4,4''-dicarboxylic acid (TPDC-NH_2) ... 13

S1.4 Synthesis of 3,3''-diamino-5'-(3-amino-4-carboxyphenyl)-[1,1':3',1''-terphenyl]-4,4''-dicarboxylic acid (BTB-NH_2) ... 19

S2 Synthesis of Protein@MOFs by biomimetic mineralisation. ... 29

S2.1 BSA@ZnBDC-R (where R= -H, -OH, -CH_3, NH_2, Br) ... 29

S2.2 ZnBDC-NH_2 I and HRP@ZnBDC-NH_2 I ... 29

S2.3 ZnBDC-NH_2 II and HRP@ZnBDC-NH_2 II ... 29

S2.4 HRP@ZnBPDC-NH_2 and ZnBPDC-NH_2 ... 29

S2.5 HRP@ZnTPDC-NH_2 and ZnTPDC-NH_2 ... 30

S2.6 Attempted synthesis of HRP@ZnBTB-NH_2 ... 30

S3 Characterisation of Protein@MOFs ... 30

S3.1 Characterisation methods ... 30

S3.2 Protein Quantification ... 31

S3.3 Evaluation of BSA@ZnBDC-R Formation (where R= -H, -OH, -CH_3, NH_2, Br) ... 32

S3.4 Characterisation of protein@ZnBDC-NH_2 ... 33

S3.5 Characterisation of $ZnBPDC-NH_2$ and $HRP@ZnBPDC-NH_2$ 42
S3.6 Characterisation of $ZnTPDC-NH_2$ and $HRP@ZnTPDC-NH_2$ 45
S3.7 PXRD Pattern Recorded for $HRP@ZnBTB-NH_2$ 48
S3.8 Comparison of Key Protein@MOF Parameters 48
S4 Kinetic Analysis *49*
S4.1 General Kinetics Protocol 49
S4.2 Kinetic Data for HRP@MOF Samples 50
S5 References *52*

S1 Ligand Synthesis and Characterisation

S1.1 Materials and Methods

Reagents purchased from commercial sources: 2-aminoterephthalic acid, Coomassie protein assay reagent, albumin standard, and zinc nitrate hexahydrate were purchased from Thermo Scientific. Methyl-2-amino-4-bromobenzoate, benzene-1,4-diboronic acid, and bis(pinacolato)diboron were purchased from Apollo Scientific. The o-phenylenediamine was purchased from Alfa Aesar. Hydrogen peroxide 30%, caesium fluoride, sodium sulfate and tetrahydrofuran were purchased from Fisher Scientific. Potassium acetate, sodium chloride and terephthalic acid were purchased from Acros Organics. The [1,1'-bis(diphenylphosphino)ferrocene] dichloropalladium (II), 2-hydroxyterephthalic acid and 2-methylterephthalic acid were purchased from Fluorochem. The 1,4-dioxane, methanol, ethanol, dichloromethane, ethyl acetate, hexane, 2-bromoterephthalic acid were purchased from Sigma-Aldrich. Deuterated NMR solvents were provided by Cambridge Isotope Laboratories Inc., Apollo Scientific, and Sigma-Aldrich. The ^{1}H and ^{13}C NMR spectra were acquired using a 500 MHz B500 Bruker Advance II spectrometer. Chemical shifts were reported in parts per million (ppm) and coupling constants (*J*) were expressed in hertz (Hz). Standard multiplicity abbreviations were used as follows: 's' for singlet, 'd' for doublet, 't' for triplet, 'dd' for doublet of doublets, and 'm' for multiplet. High-resolution mass spectra were generated with a Thermo Orbitrap Exactive Plus Extended Mass Range mass spectrometer. Elemental analyses were performed by the microanalytical services of The University of Manchester with a CFlash 2000 elemental analyser for the analyses of carbon, hydrogen and nitrogen.

S1.2 Synthesis of 3,3'-diamino-[1,1'-biphenyl]-4,4'-dicarboxylic acid (BPDC-NH2)

3,3'-Diamino-[1,1'-biphenyl]-4,4'-dicarboxylic acid was synthesised following a previously reported three-step procedure. [1]

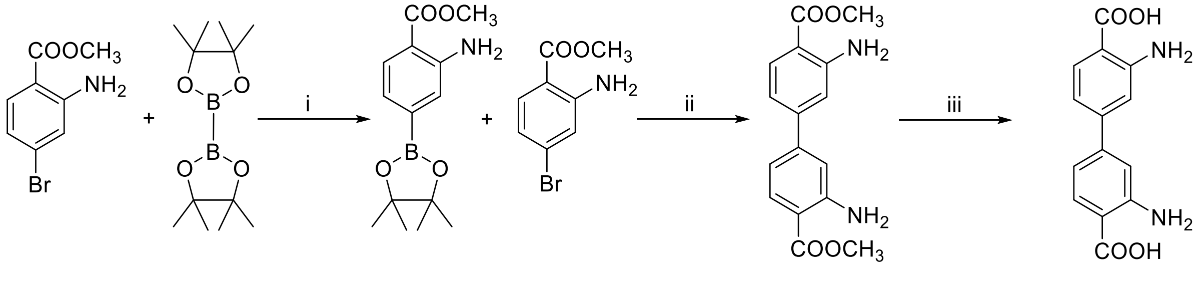

A1 A2 A3 **BPDC-NH$_2$**

Scheme S1: Synthesis of **BPDC-NH$_2$**. Reagents and conditions: (i) CH_3COOK, Pd(dppf)Cl_2, dioxane, 100 °C (reflux), 16 h, 52%; (ii) CsF, Pd(dppf)Cl_2, *p*-dioxane/H_20 (1:1), 90 °C (reflux), 24 h, 48%; (iii) NaOH, THF, 50 °C (reflux), 48 h, 92%.

3-Amino-4-methoxycarbonyl-phenylboronic acid pinacol ester (A2):

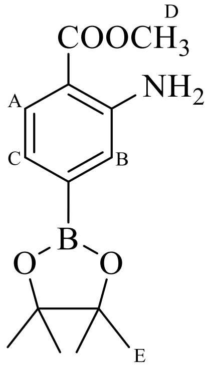

A2

Methyl-2-amino-4-bromobenzoate (**A1**) (1.08 g, 5.0 mmol), bis(pinacolato)diboron (1.27g, 5.0 mmol) and potassium acetate (1.47 g, 15 mmol) were added to dioxane (20 mL). The mixture was purged with nitrogen for 30 min. Pd(dppf)Cl_2 (200 mg, 0.27 mmol) was then added to the mixture. The reaction was stirred at 100 °C for 16 hours under nitrogen. Once cooled to room temperature, the reaction was diluted with CH_2Cl_2 (50 mL). The organic phase was then washed with H_2O (2 × 400 mL) and saturated NaCl solutions (200 mL). After drying over Na_2SO_4, the organic solvent was removed in a vacuum. The crude product was further purified via flash column chromatography (hexane/ethyl acetate 10:1) to yield a white solid **A2** (1.21 g, 86%).**^{1}H NMR** (500 MHz, 298 K, $CDCl_3$) δ = 7.85 (d, J = 7.9 Hz, 1H, H^A), 7.21 (d, J = 1.1 Hz, 1H, H^B), 7.11 (dd, J = 7.9, 1.1 Hz, 1H, H^C), 3.88 (s, 3H, H^D), 1.34 (s, 12H, H^E). **^{13}C NMR** (126 MHz, 298 K, $CDCl_3$) δ = 168.64, 148.96, 130.38, 123.80, 122.45, 113.20, 84.28, 51.77, 24.99. **HRMS** (ESI^+): Calcd. for $C_{14}H_{20}B_NO_4H^+$: 275.1561, found 278.1556 $[M+H]^+$.

Data matches that previously reported. [1]

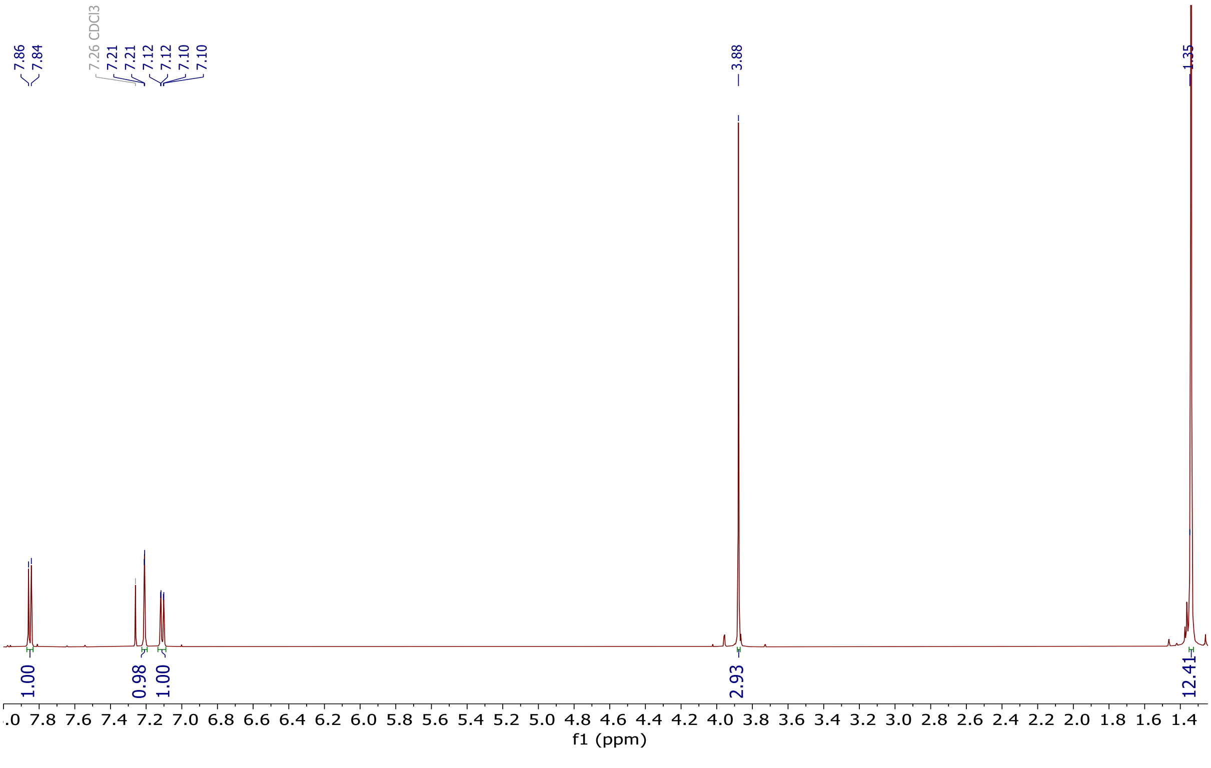

Figure S1: ^{1}H NMR (500 MHz, $CDCl_3$, 298 K) spectrum of **A2** between 1.2 and 8.0 ppm.

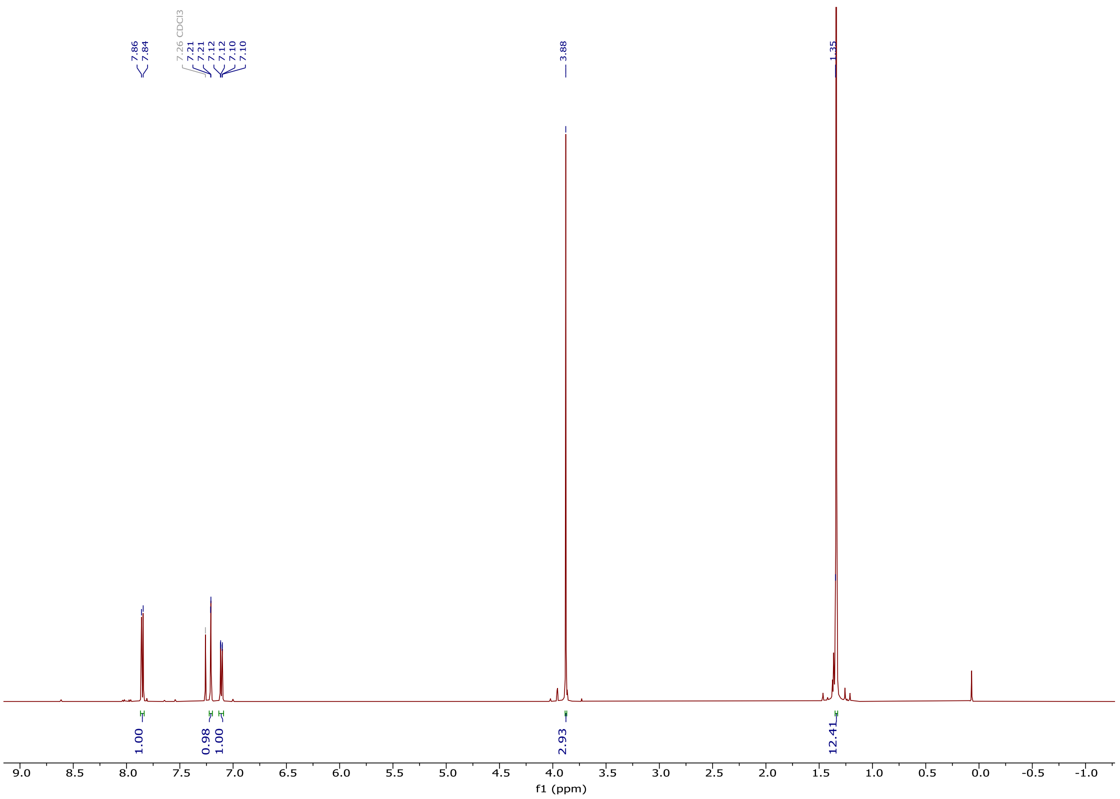

Figure S2: ^{1}H NMR (500 MHz, $CDCl_3$, 298 K) spectrum of **A2**.

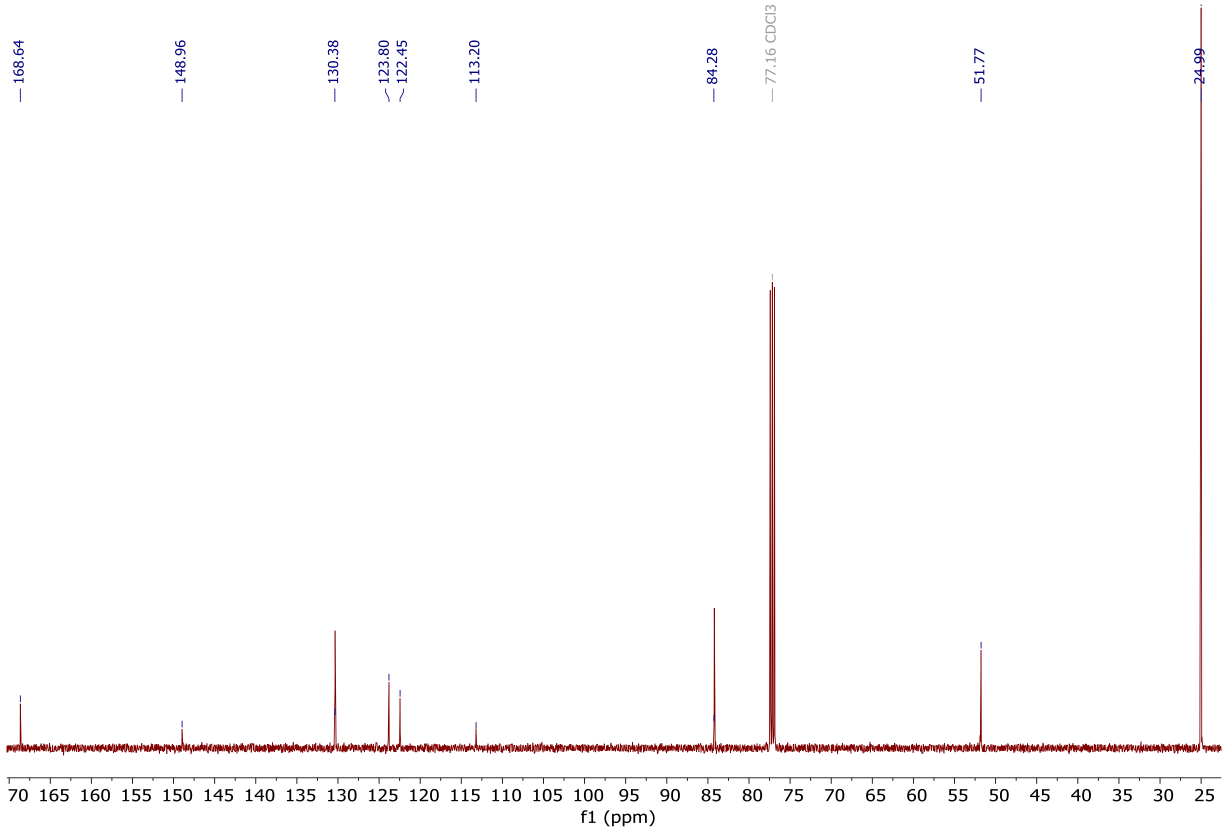

Figure S3: ^{13}C NMR (126 MHz, $CDCl_3$, 298 K) spectrum of **A2** between 170 and 25 ppm.

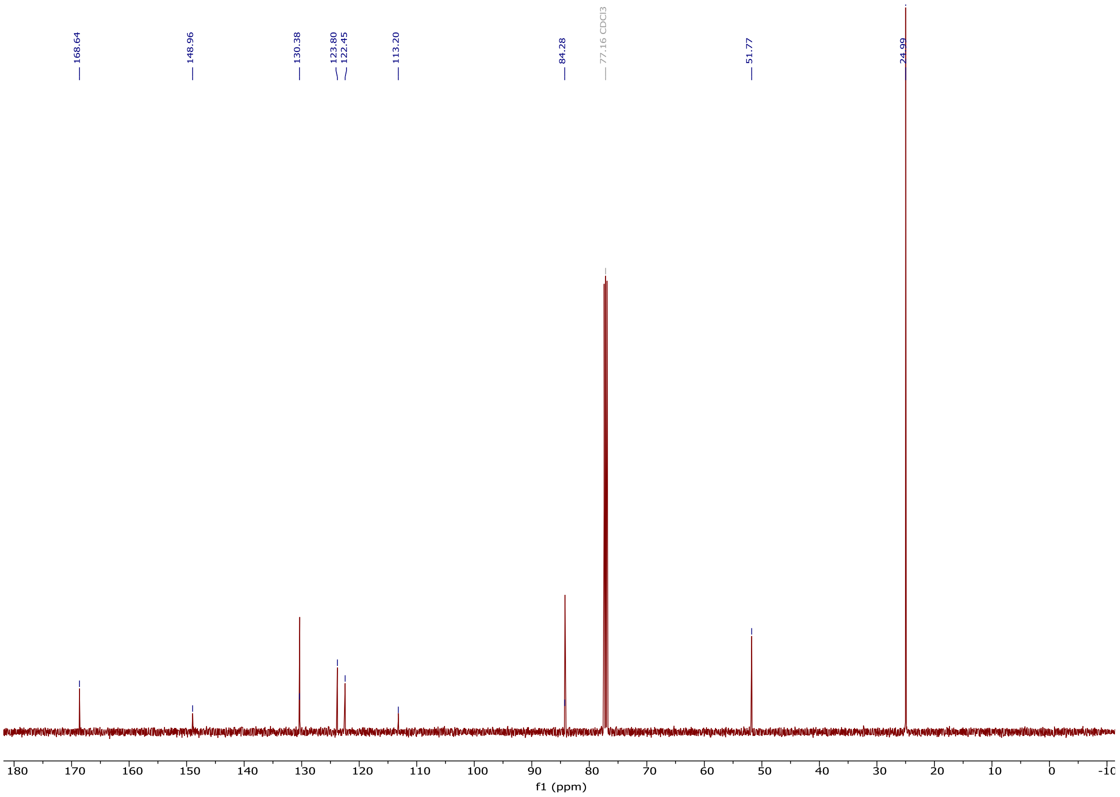

Figure S4: ^{13}C NMR (126 MHz, $CDCl_3$, 298 K) spectrum of **A2**.

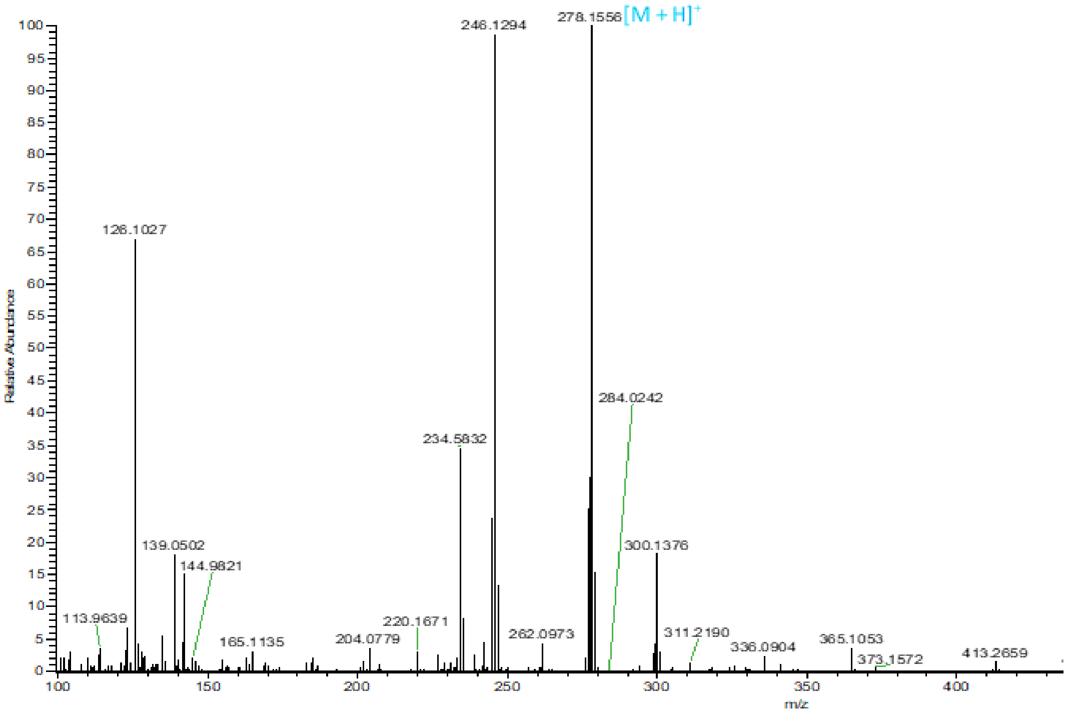

Figure S5: High resolution mass spectrum (ESI^+) for **A2**: 278.56 $[M+H]^+$.

Dimethyl 3,3'-diamino-1,1'-biphenyl-4,4'-dicarboxylate (A3):

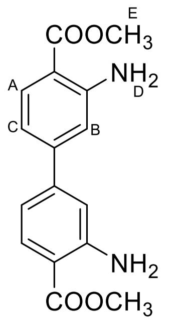

A3

A2 (1.00 g, 3.8 mmol), methyl 2-amino-4-bromobenzoate (0.98 g, 4.56 mmol) and caesium fluoride (1.75 g, 11.4 mmol) were added to 1: 1 mixture of dioxane/H_2O. The reaction mixture was then degassed by freeze pump thaw cycles (3×) followed by the addition of Pd(dppf)Cl_2 (150 mg, 0.21 mmol). The reaction was then stirred at 90 °C under nitrogen for 24 hours. Once cooled to room temperature, the reaction mixture was diluted with CH_2Cl_2 (50 mL). The organic solution was washed with H_2O (2 × 500 mL) and saturated NaCl solution (200 mL). The organic phase was dried over Na_2SO_4 and solvents were removed under vacuum. The crude product was further purified via flash column chromatography (CH_2Cl_2/ethyl acetate 15:1 to 10:1) to yield an off-white solid **A3** (803 mg, 71.2%). **^{1}H NMR** (500 MHz, 298 K, DMSO-*d*$_6$) δ = 7.77 (d, *J* = 8.4 Hz, 1H, H^A), 7.03 (d, *J* = 1.8 Hz, 1H, H^B), 6.77 (dd, *J* = 1.8 Hz, 1H, H^C), 6.75 (s, 2H, H^D), 3.80 (s, 3H, H^E). **^{13}C NMR** (126 MHz, 298 K, DMSO-*d*$_6$) δ = 167.59, 151.51, 144.81, 131.34, 114.35, 113.46, 51.45. **HRMS** (ESI$^+$): Calcd. for $C_{16}H_{16}N_2O_4H^+$: 301.1183, found 300.1172 $[M+H]^+$.

Data matches that previously reported. [1]

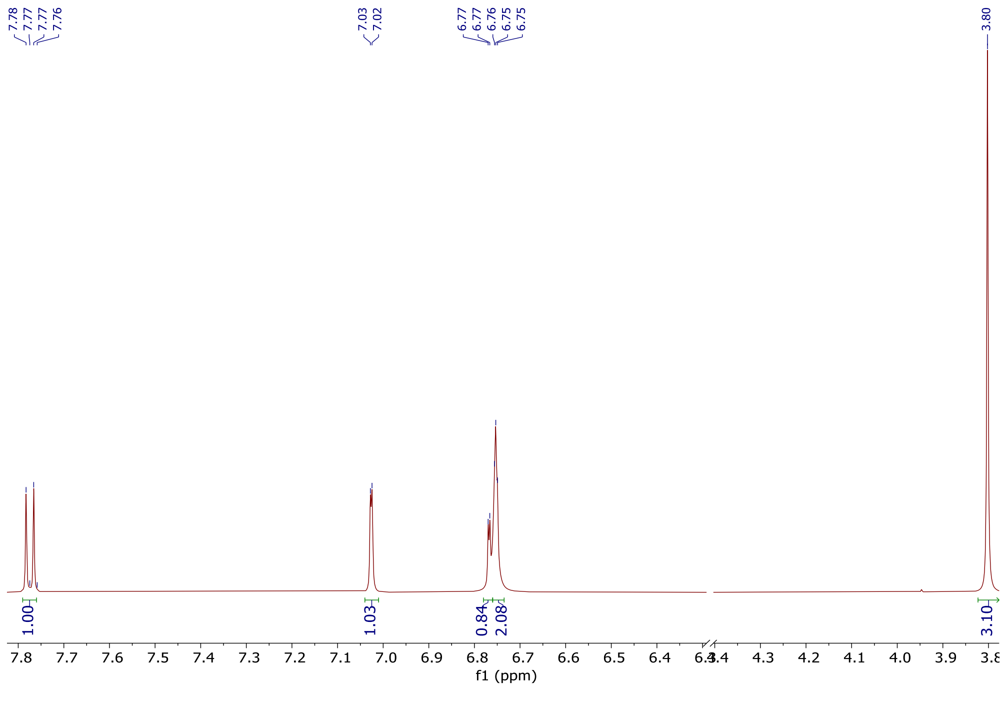

Figure S6: ^{1}H NMR (500 MHz, DMSO-*d*$_6$, 298 K) spectrum of **A3** between 3.8 and 7.8 ppm.

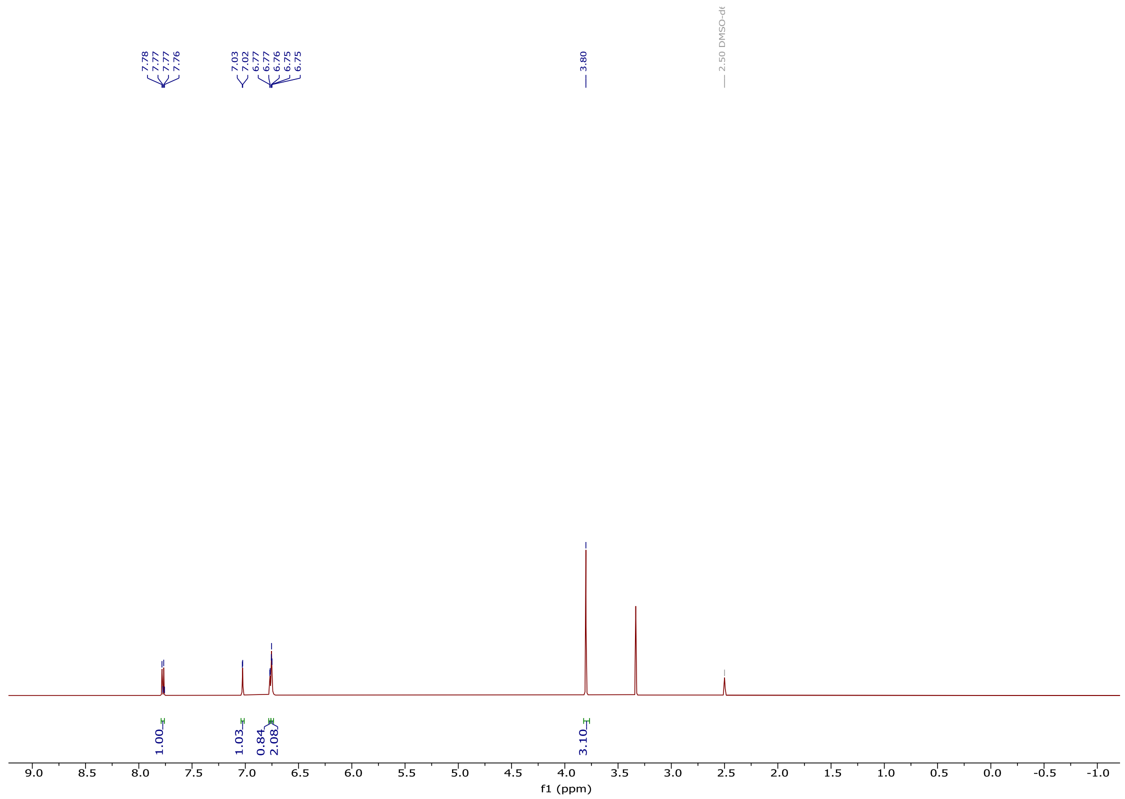

Figure S7: ^{1}H NMR (500 MHz, DMSO-d_6, 298 K) spectrum **A3**.

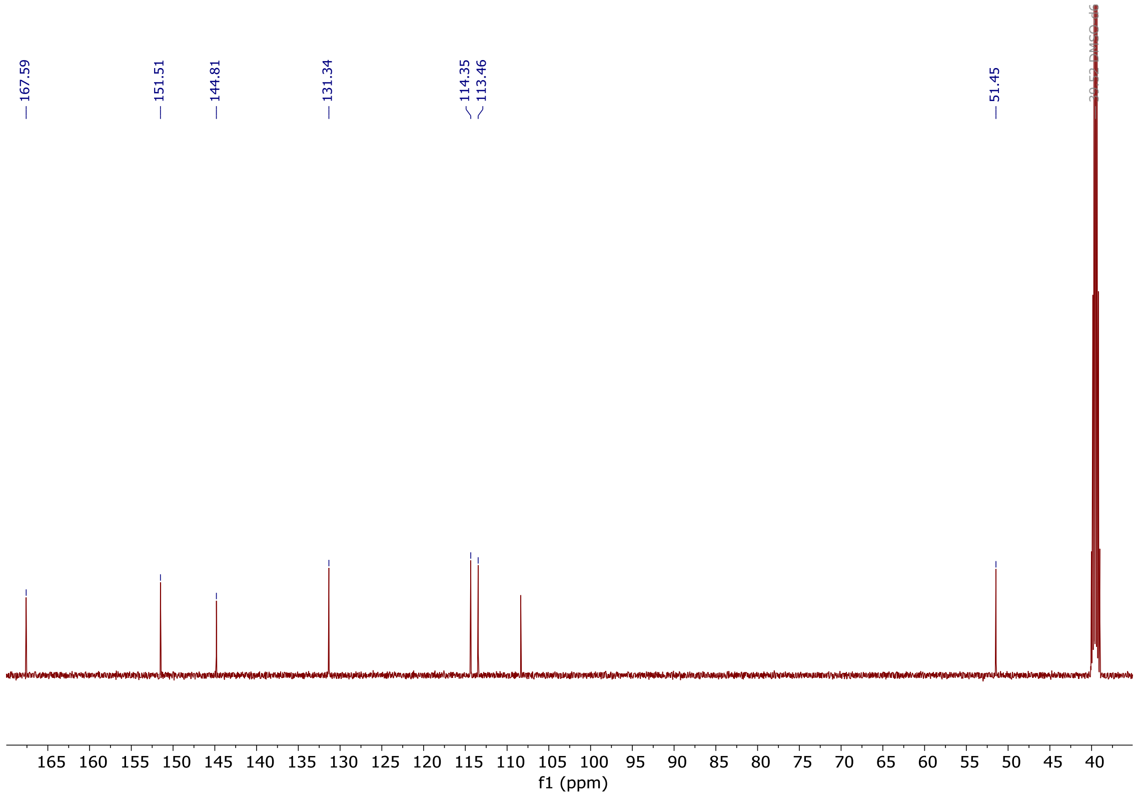

Figure S8: ^{13}C NMR (126 MHz, DMSO-d_6, 298 K) spectrum of **A3** between 35 and 170 ppm.

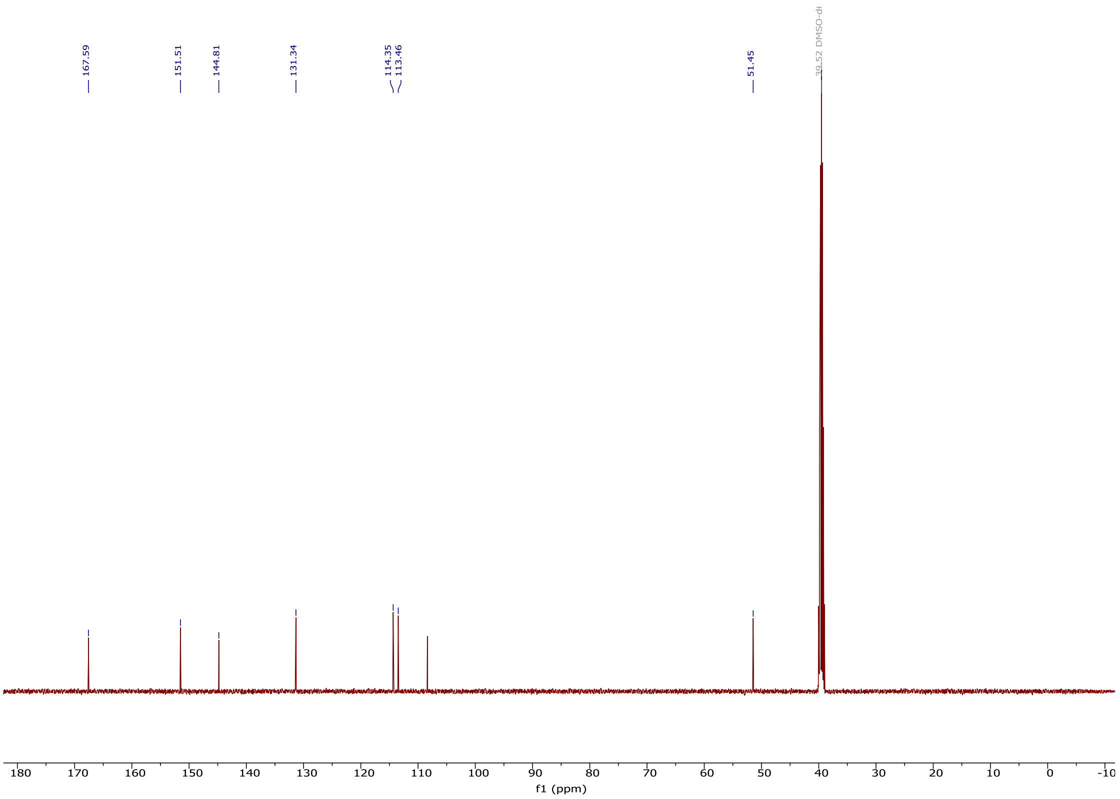

Figure S9: ^{13}C NMR (126 MHz, DMSO-d_6, 298 K) spectrum of **A3**.

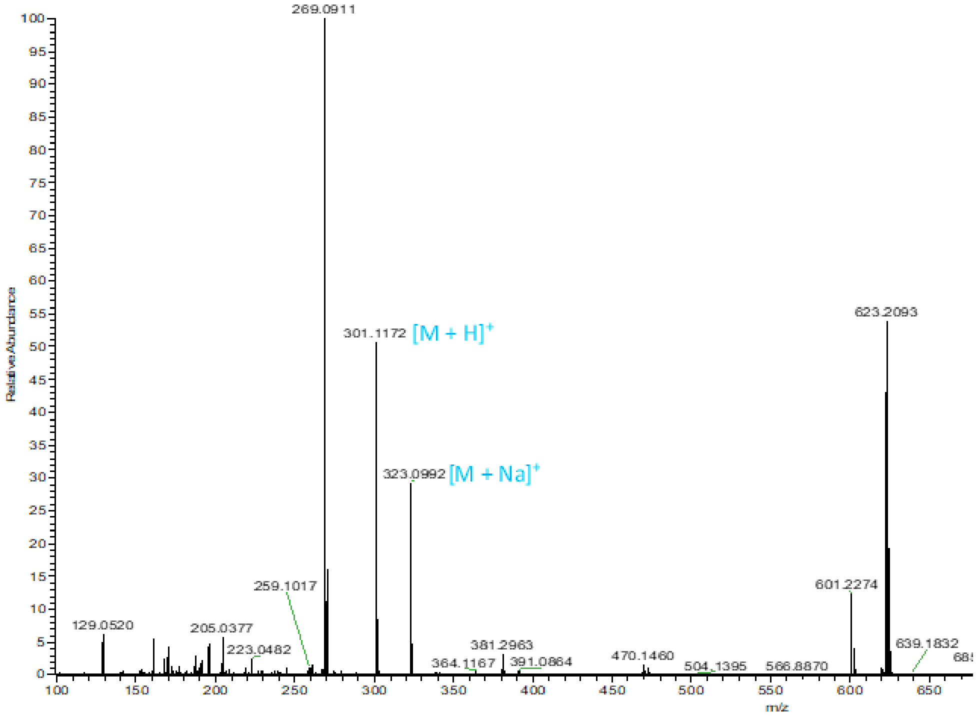

Figure S10: High resolution mass spectrum (ESI$^+$) of **A3**: 301.1172 $[M+H]^+$.

3, 3'-Diamino-1, 1'-biphenyl-4,4'-dicarboxylic acid (BPDC-NH_2):

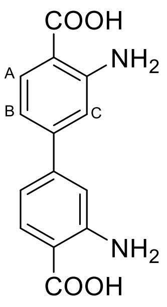

BPDC-NH_2

A3 (0.700 g, 2.33 mmol) was dissolved in 1:1 THF/MeOH (40 mL). To the mixture was added NaOH (1.1 g, 27 mmol) dissolved in water (20 mL). The reaction was heated to 50 ºC and stirred under reflux for 48 hours. Once cooled to room temperature, the organic solvent was removed in vaccuo. 1 M HCl solution was slowly added to the aqueous residue until the pH of the solution reached 5 to obtain a yellow paste. The paste was then filtered and washed with H_2O (3 × 100 mL) followed by washing with methanol (2 × 20 mL). The solid was then dried overnight and yielded a yellow solid **BPDC-NH_2** (590 mg, 95%).**^{1}H NMR** (500 MHz, 298 K, DMSO-d_6) δ = 7.76 (d, J = 8.3 Hz, 1H, H^A), 6.98 (d, J = 1.8 Hz, 1H, H^B), 6.73 (dd, J = 8.4, 1.8 Hz, 1H, H^C). **^{13}C NMR** (126 MHz, 298 K, DMSO-d_6) δ = 169.35, 151.70, 144.77, 131.87, 114.17, 113.33, 109.20. **HRMS** (ESI^+): Calcd. for $C_{16}H_{12}N_2O_4H^+$: 273.0870, found 273.857 $[M+H]^+$.

Data matches that previously reported. [1]

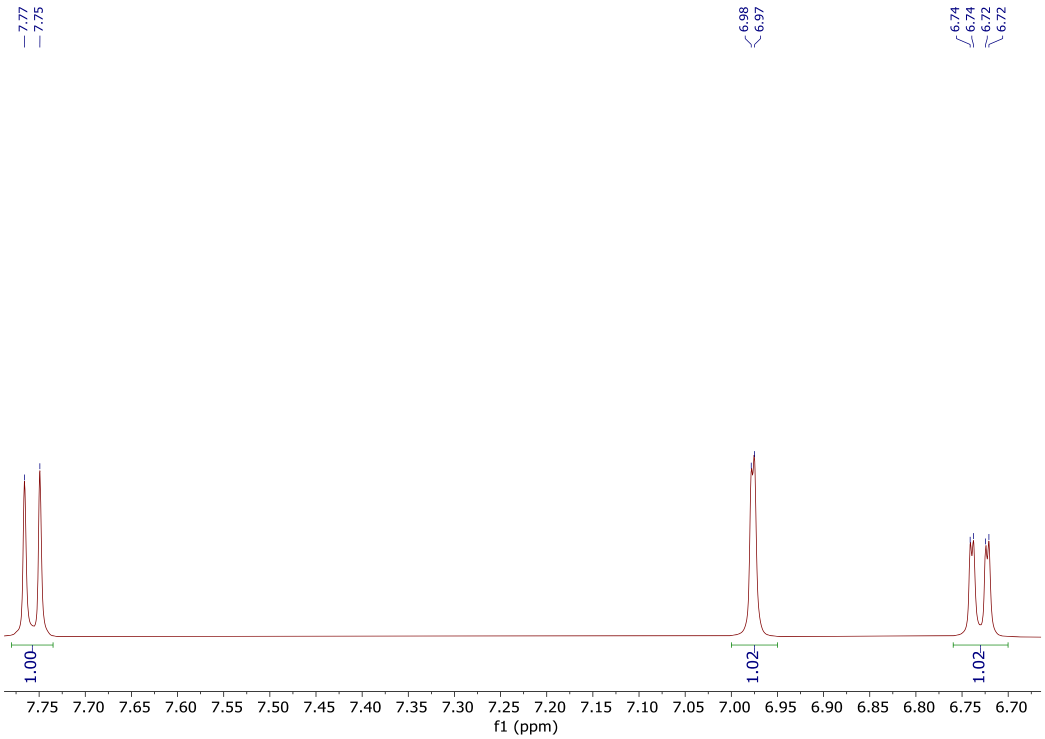

Figure S11: ^{1}H NMR (500 MHz, DMSO-d_6, 298 K) spectrum of **BPDC-NH_2** between 6.65 and 7.8 ppm.

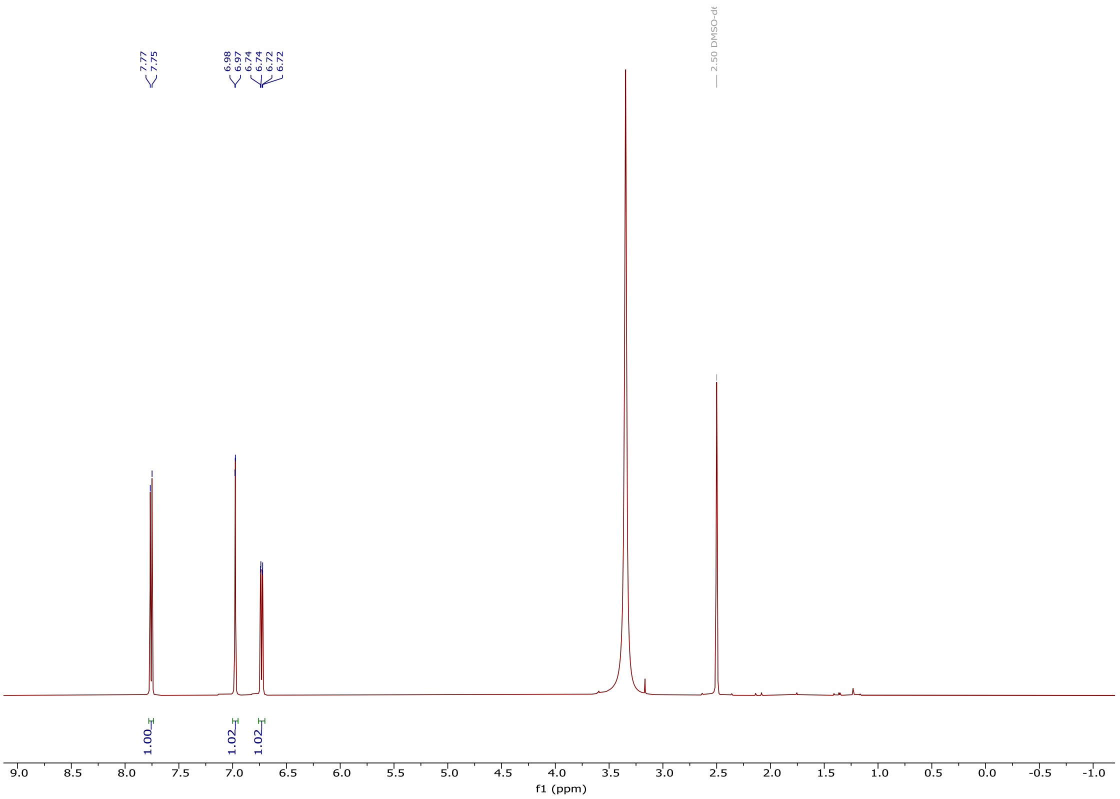

Figure S12: ^{1}H NMR (500 MHz, DMSO-d_6, 298 K) spectrum of **BPDC-NH$_2$**.

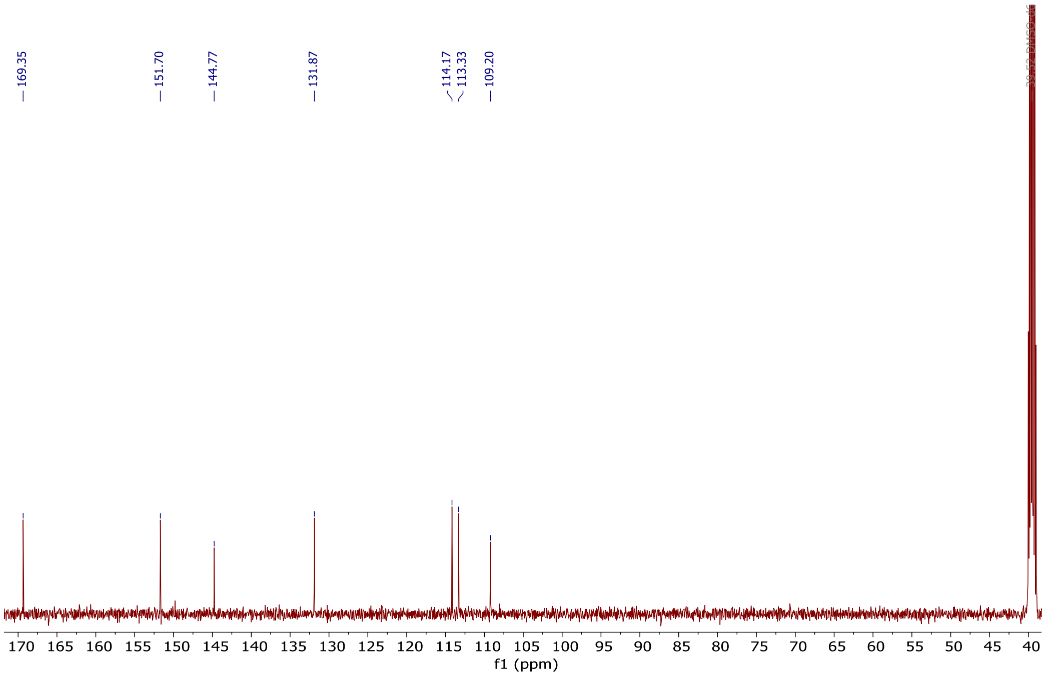

Figure S13: ^{13}C NMR Spectra of **BPDC-NH$_2$**: (126 MHz, DMSO-d_6, 298 K) between 40 and 170 ppm.

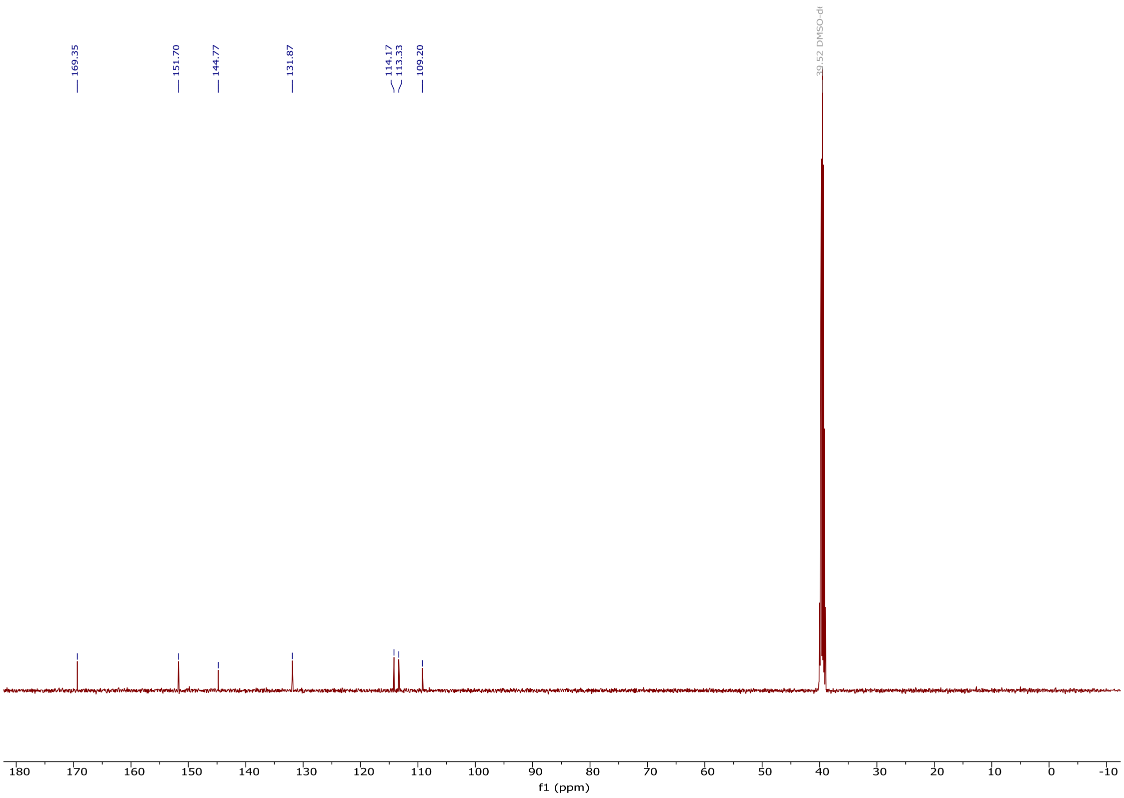

Figure S14: ^{13}C NMR Spectra of **BPDC-NH$_2$**: (126 MHz, DMSO-d_6, 298 K).

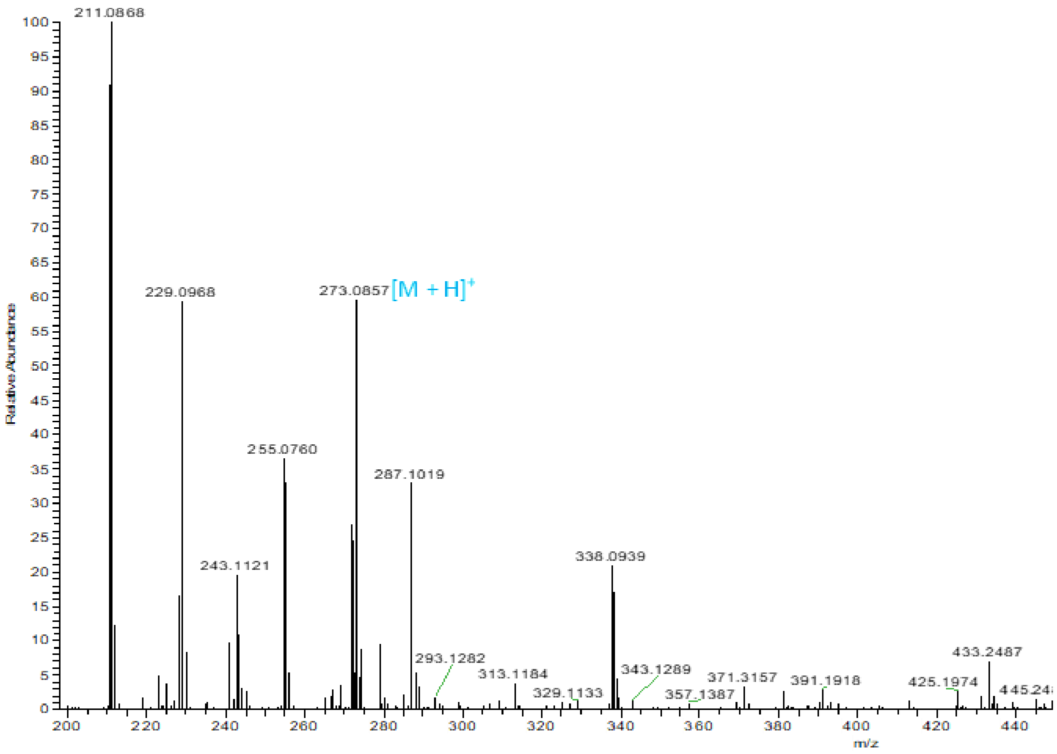

Figure S15: High resolution mass spectrum (ESI$^+$) for **BPDC-NH$_2$**: 273.0857 $[M+H]^+$.

S1.3 Synthesis of 3,3''-diamino-5'-(3-amino-4-carboxyphenyl)-[1,1':3',1''-terphenyl]-4,4''-dicarboxylic acid (TPDC-NH_2)

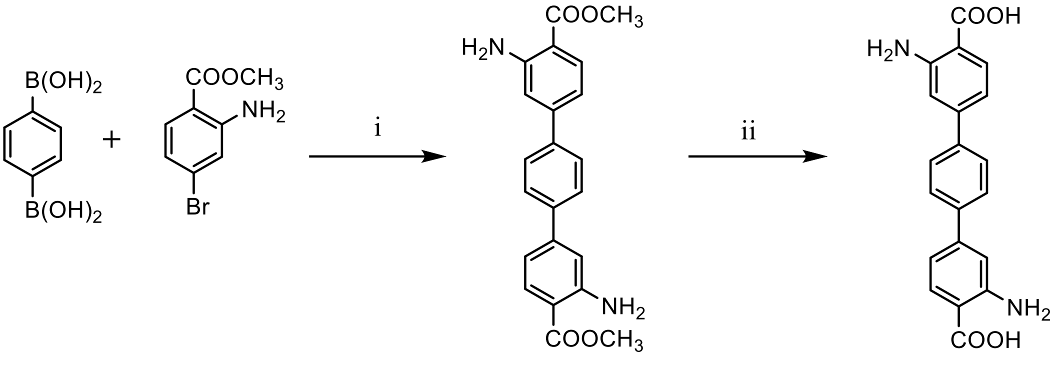

B1 **B2** **TPDC-NH_2**

Scheme S2: Synthesis of ligand **TPDC-NH_2**, reagents and conditions: (i) Pd(dppf)Cl_2, CsF, dioxane : H_2O 1:1, 100 °C (reflux), 24 h; (ii) Sodium hydroxide, MeOH/THF/H_2O, (reflux).

Dimethyl 3, 3''-diamino-1,1':4',1''-terphenyl-4,4''-dicarboxylate (B2):

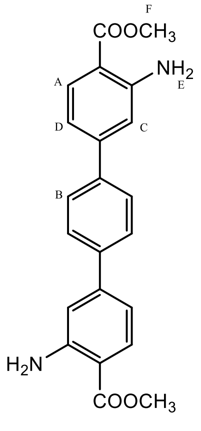

B2

Methyl-2-amino-4-bromobenzoate (B1) (1.15 g, 5 mmol), 1,4-phenylenediboronic acid (331.5 mg, 2 mmol) and caesium fluoride (1.822 g, 12 mmol) were dissolved into a 1:1 mixture solution of dioxane/H_2O (60 mL) with stirring. The reaction solution was purged with nitrogen for 30 minutes. Pd(dppf)Cl_2 was dissolved into the mixed solution under a nitrogen environment, then the reaction mixture was stirred and refluxed at 100 °C for 24 hours. The reaction was diluted with dichloromethane (100 mL). The organic solution was washed with DI water (200 mL) twice and then with saturated NaCl solution (200 mL). After drying with anhydrous sodium sulfate, the organic solvent was removed using a rotary evaporator. Yellow crystals of (**B2**) (250 mg, 14%) were isolated after silica gel column chromatography (CH_2Cl_2/ EtOAc 40:1 to 10:1). **^{1}H NMR** (500 MHz, 298 K, DMSO-d_6) δ = 7.79 (d, J=8.4 Hz, 2H, H^A), 7.73 (s, 4H, H^B), 7.14 (d, J=1.6 Hz, 2H, H^C), 6.89 (dd, J=8.4 Hz,1.6 Hz, 2H, H^D), 6.76(s, 4H, H^E), 3.88 (s, 6H, H^F). **^{13}C NMR** (126 MHz, 298 K, DMSO-d_6), δ = 167.63, 151.62, 144.63, 139.07,131.42, 127.16, 114.09, 113.48, 108.03, 51.44. **HRMS** (ESI$^-$): Calculated for $C_{22}H_{19}N_2O_4^-$: 375.41, found 375.1361 $[M-H]^-$.

Data matches that previously reported.[1]

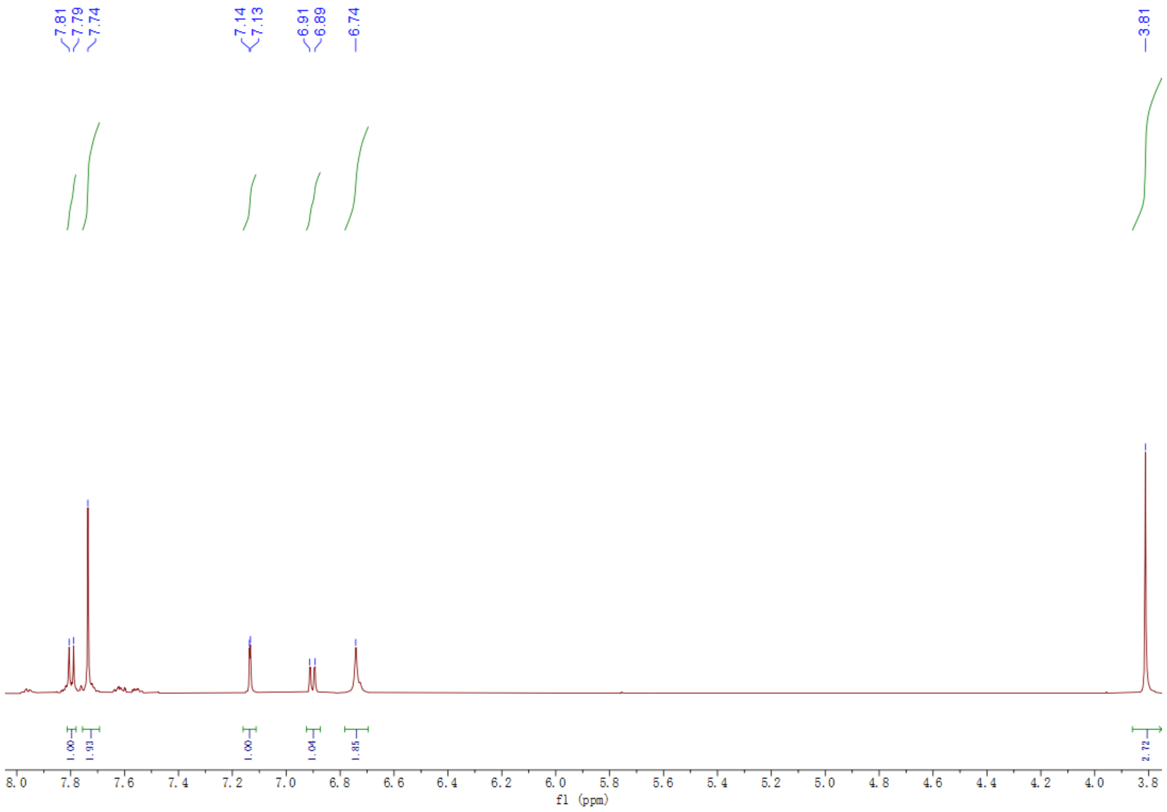

Figure S16: ^{1}H NMR (500 MHz, DMSO-d_6, 298 K) spectra of **B2** between 3.8 and 8.0 ppm.

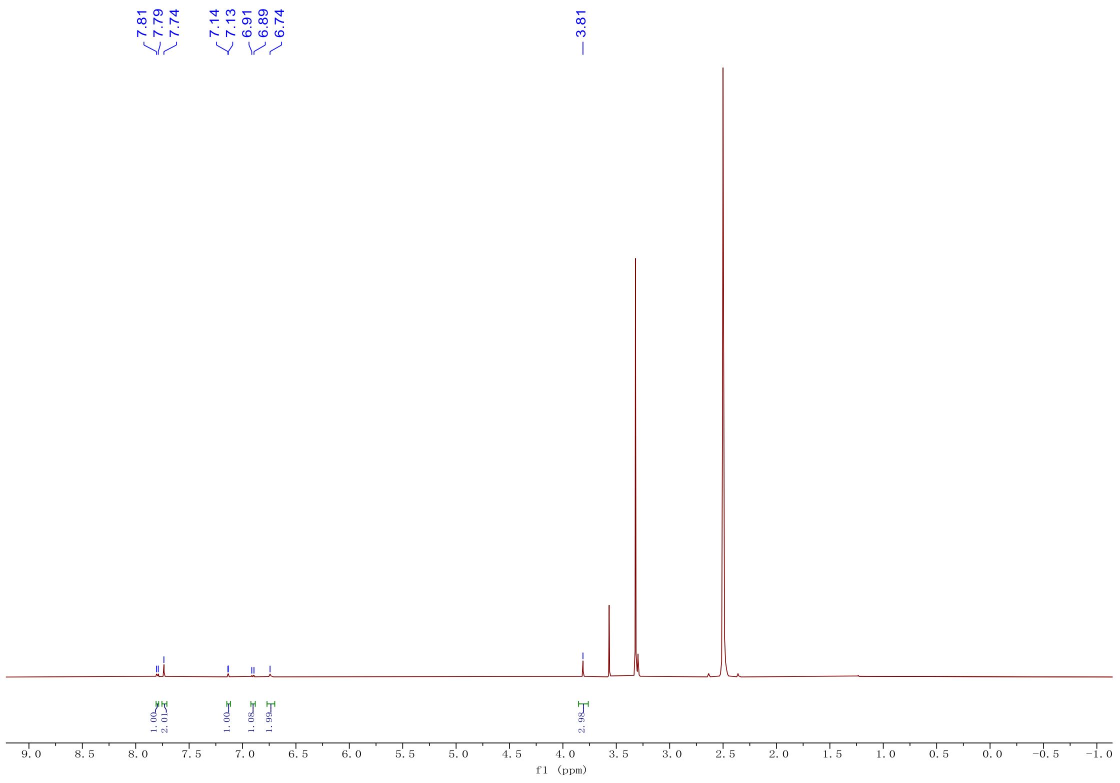

Figure S17: ^{1}H NMR (500 MHz, DMSO-d_6, 298 K) spectra of **B2**.

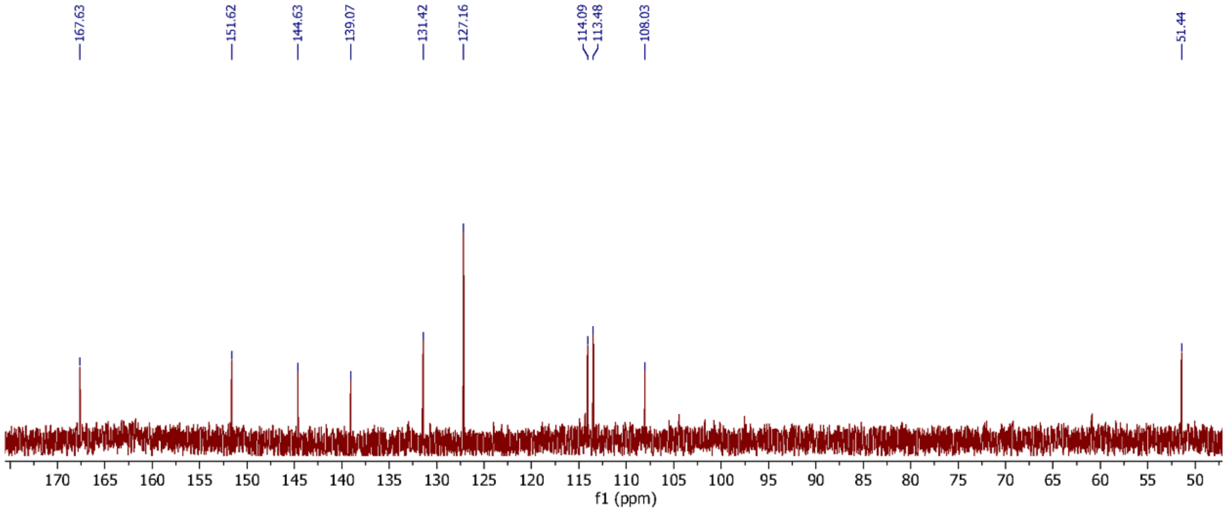

Figure S18: ^{13}C NMR (126 MHz, DMSO-d_6, 298 K) spectra of **B2** between 50 and 175 ppm.

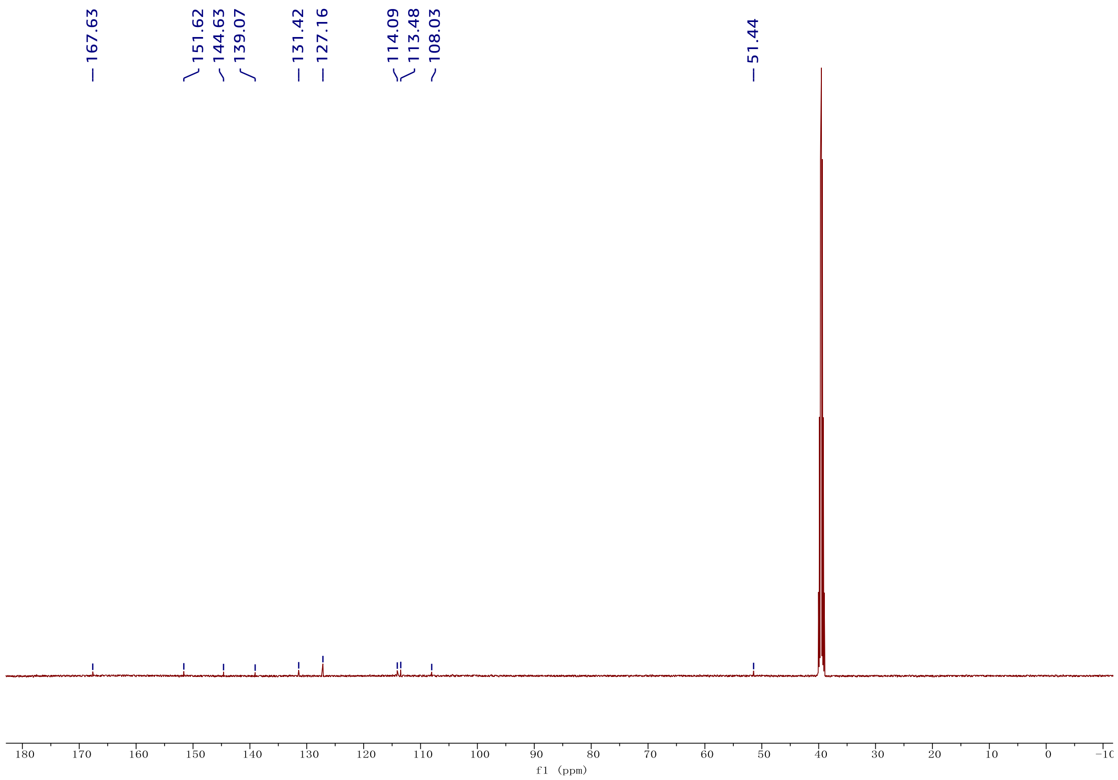

Figure S19: ^{13}C NMR (126 MHz, DMSO-d_6, 298 K) spectra of **B2**.

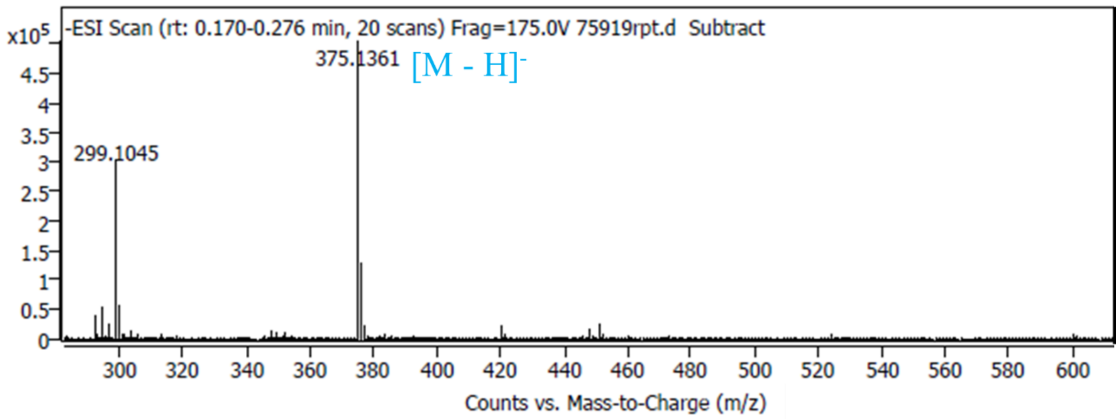

Figure S20: Mass spectrum (ESI-) for **B2**, found 375.1361 [M-H]$^-$.

3,3''-Diamino-5'-(3-amino-4-carboxyphenyl)-[1,1':3',1''-terphenyl]-4,4''-dicarboxylic acid (TPDC-NH$_2$):

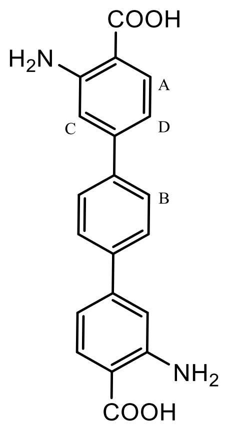

TPDC-NH$_2$

Dimethyl 3, 3''-diamino-1,1':4',1''-terphenyl-4,4''-dicarboxylate (200 mg, 0.53 mmol) was fully dissolved into a 1:1 THF/MeOH solution (30 mL). Next 15.0 mL sodium hydroxide solution (1 M) was added to the mixture. The reaction mixture was heated under reflux with stirring for 24 hours. The organic solvent was removed by a rotary evaporator after the mixture was cooled to room temperature. The pH of the aqueous solution was adjusted to below 5 by dropwise addition of 1 M HCl solution, yielding a yellow precipitate. After centrifugation, the precipitate was washed with DI water twice and lyophilised yielding a yellow powder-like solid of **TPDC-NH$_2$**. (140 mg, 76%). **^{1}H NMR (**500 MHz, 298 K, DMSO-*d$_6$*), δ = 7.78 (d, *J*=8.4 Hz, 2H, H^A), 7.72 (s, 4H, H^B), 7.09 (d, *J*=1.5 Hz, 2H, H^C), 6.87 (dd, *J*=8.4 Hz, 1.5Hz, 2H, H^D).**^{13}C NMR** (126 MHz, 298 K, DMSO-*d$_6$*) δ = 169.36, 151.71, 144.44, 139.17, 131.99, 127.14, 113.98, 113.37, 109.00. **HRMS** (ESI$^-$): Calculated for $C_{20}H_{15}N_2O_4^-$: 347.11, found 347.10 [M-H]$^-$.

Data matches that previously reported.[1]

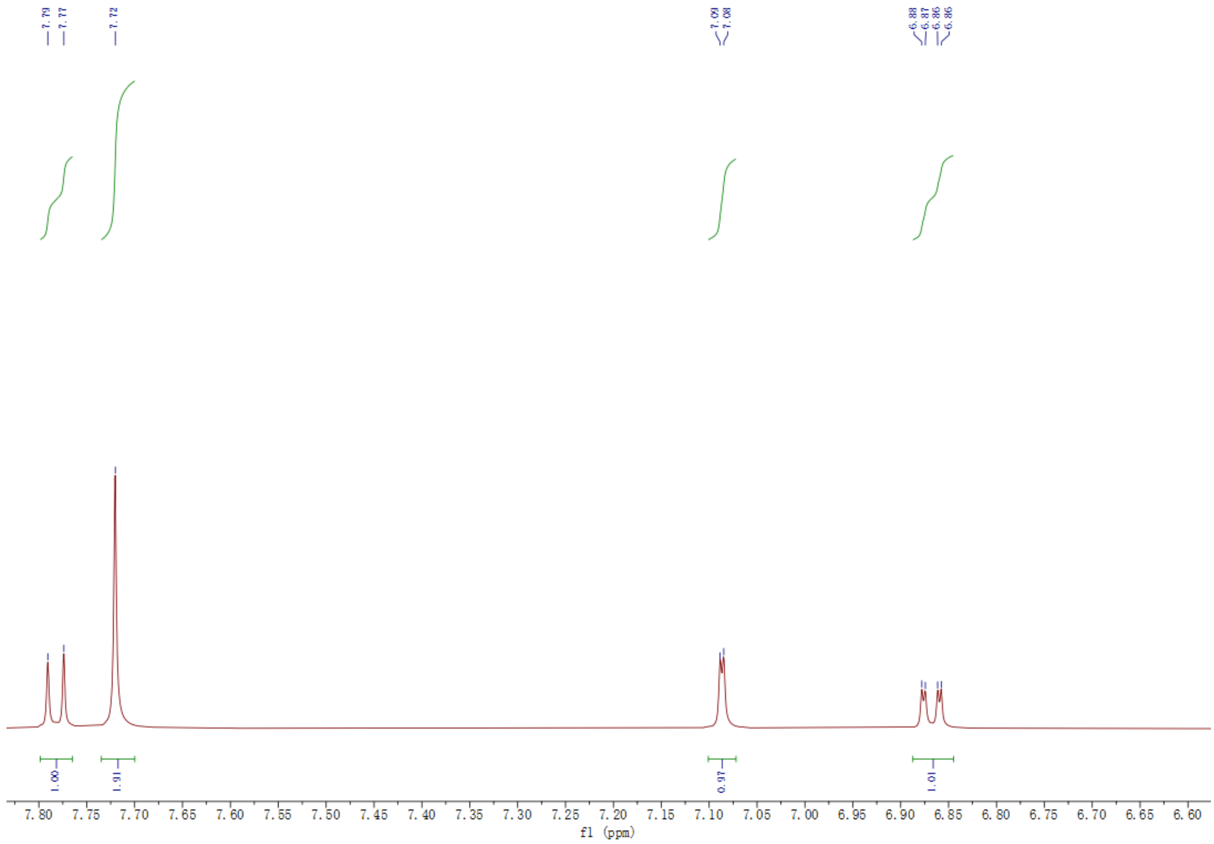

Figure S21: ^{1}H NMR (500 MHz, DMSO-d_6, 298 K) spectrum of **TPDC-NH$_2$** between 6.6 and 7.85 ppm.

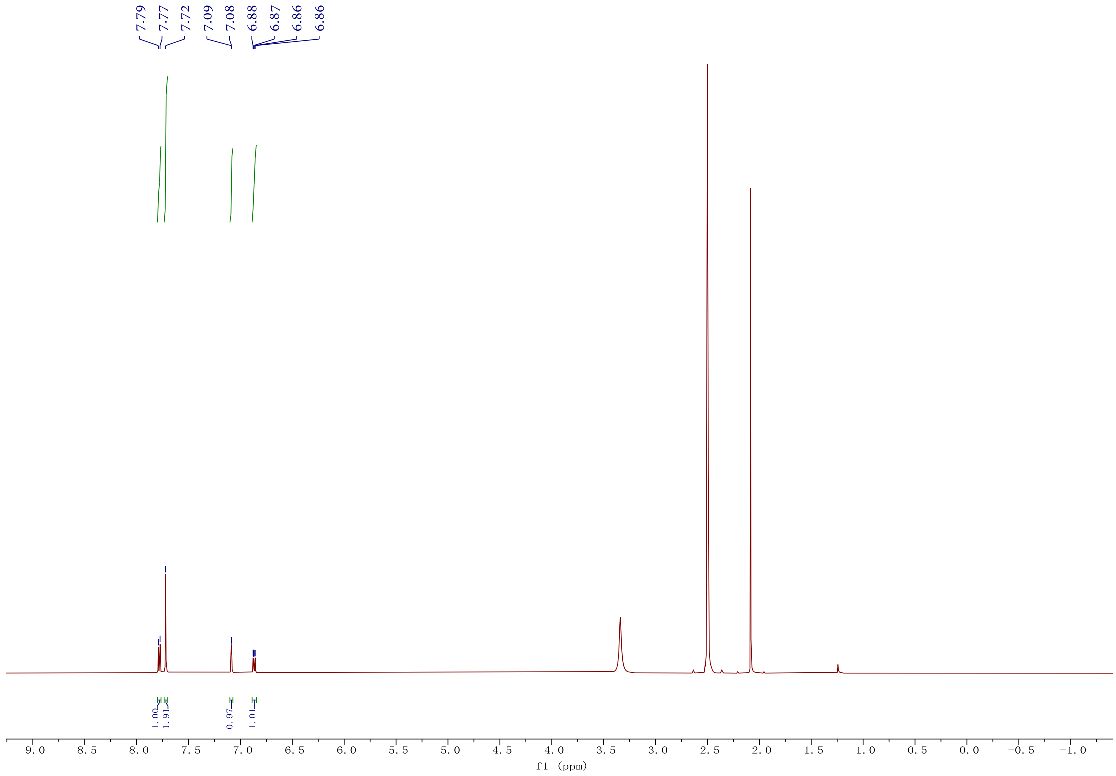

Figure S22: ^{1}H NMR (500 MHz, DMSO-d_6, 298 K) spectrum of **TPDC-NH$_2$**.

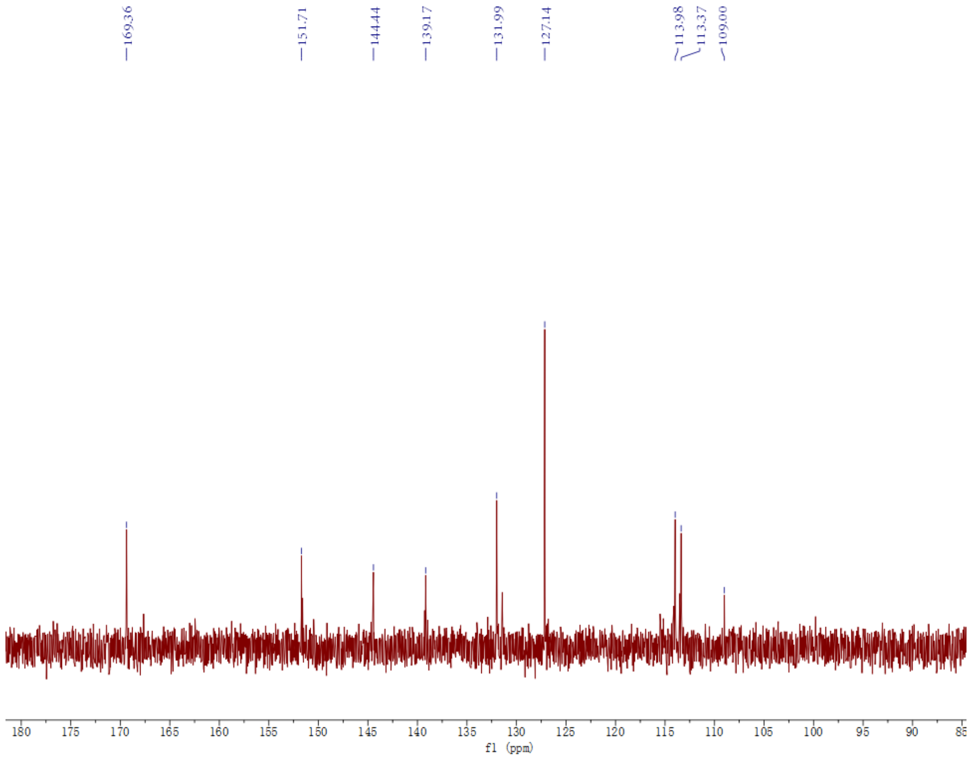

Figure S23: ^{13}C NMR (126 MHz, DMSO-d_6, 298 K) spectrum of **TPDC-NH$_2$** between 85 and 180 ppm.

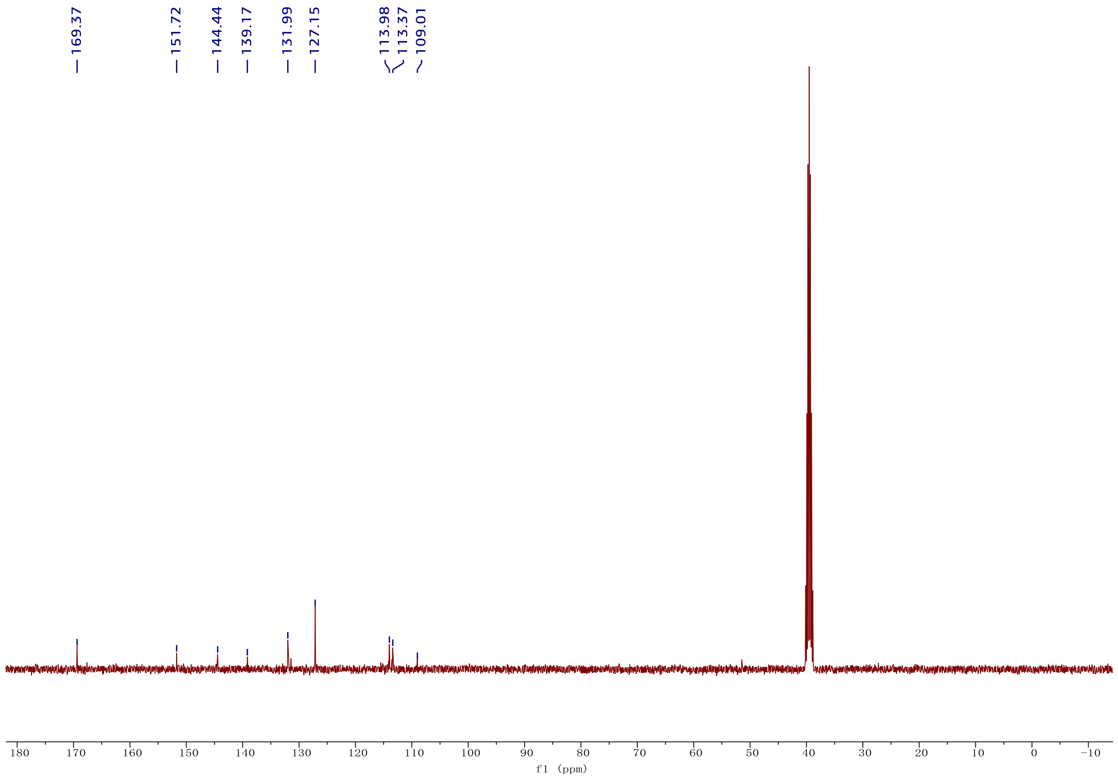

Figure S24: ^{13}C NMR (126 MHz, DMSO-d_6, 298 K) spectrum of **TPDC-NH$_2$**.

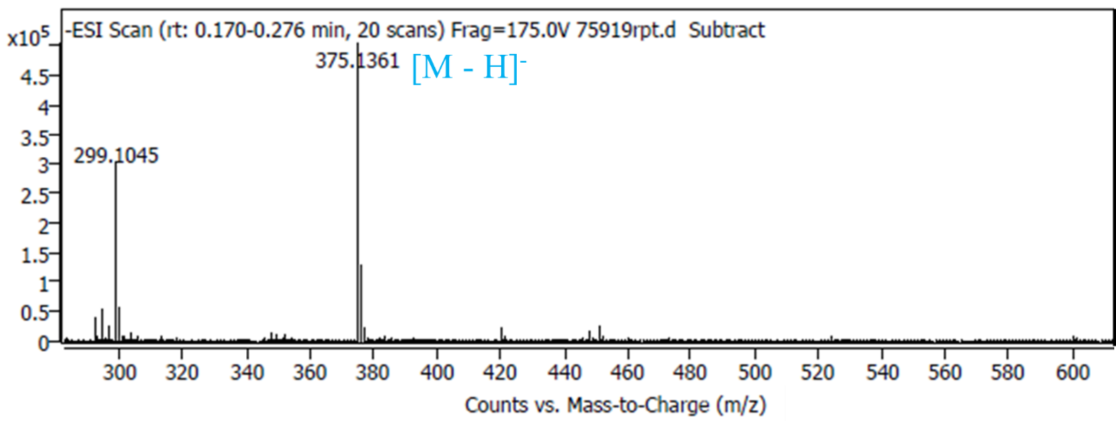

Figure S25: Mass Spectrum (ESI-) for **TPDC-NH$_2$**, 347.10 [M-H]$^-$.

S1.4 Synthesis of 3,3''-diamino-5'-(3-amino-4-carboxyphenyl)-[1,1':3',1''-terphenyl]-4,4''-dicarboxylic acid (BTB-NH$_2$)

Synthetic route to 3,3''-diamino-5'-(3-amino-4-carboxyphenyl)-[1,1':3',1''-terphenyl]-4,4''-dicarboxylic acid following previously reported[2] three-step procedure.

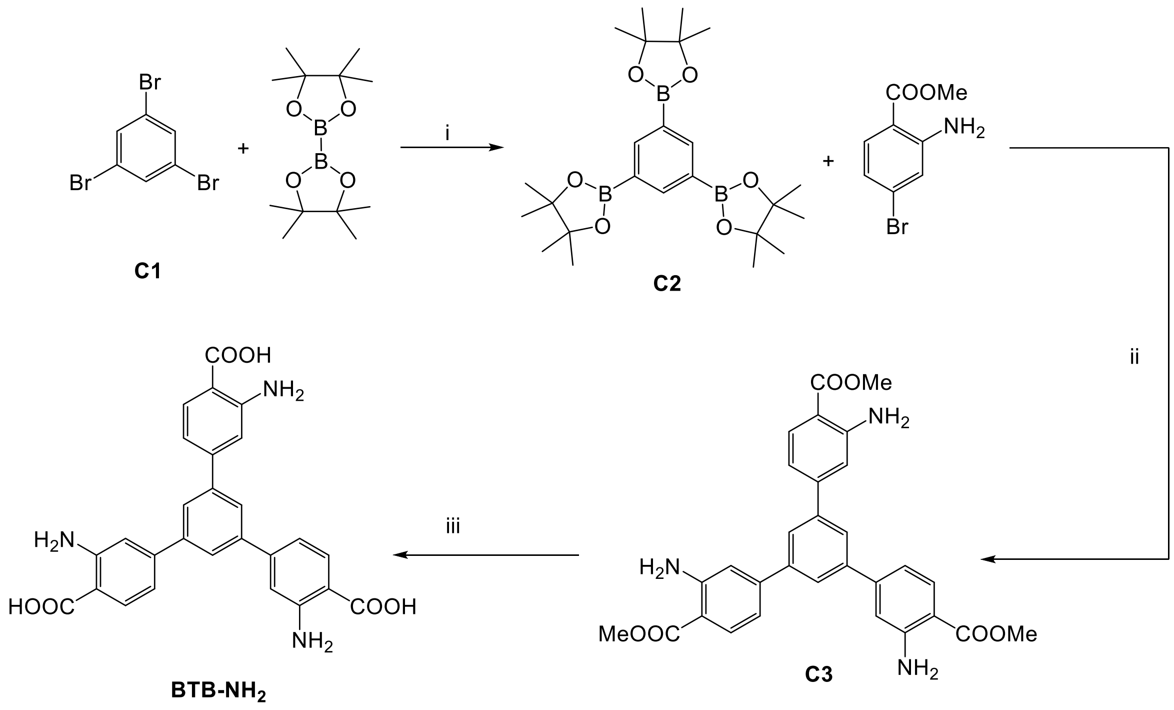

Scheme S3: Synthesis of ligand **BTB-NH$_2$**. Reagents and conditions: (i) CH_3COOK, Pd(dppf)Cl_2, DMF, 90 °C, 24 h, 52%; (ii) CsF, Pd(dppf)Cl_2, *p*-dioxane/$H_2$0 (1:1), 90 °C, 24 h, 48%; (iii) NaOH, THF, 50 °C, 48 h, 92%.

1,3,5-tris(4,4,5,5-tetramethyl-1,3,2-dioxaborolan-2-yl) benzene (C2):

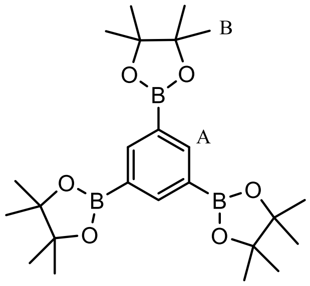

C2

Anhydrous DMF (10 mL) was purged with N_2 and then was transferred via a syringe into a round bottom flask with 1,3,5 – tribromobenzene (1.00 g, 3.17 mmol) and bis(pinacolato)diboron (2.54 g, 9.53 mmol). Potassium acetate (1.87 g, 19.0 mmol) and Pd(dppf)Cl_2 (0.087 g, 0.12 mmol) were then quickly added into the flask. The mixture was stirred and heated at 90 ºC for 24 hours. The flask was later cooled down to room temperature, DI water (120 mL) was added. Black precipitate in the solution was collected via filtration and washed with deionized water three times. The black solid was further purified by flash column chromatography using hexane/EtOAc 5:1 to 3:1 and yielded **C2** as an off-white solid. (1.330 g 92%) ^{1}**H NMR** (500 MHz, 298 K, $CDCl_3$) δ = 8.36 (s, 1H, H^A), 1.33 (s, 12H, H^B); ^{13}**C NMR** (126 MHz, 298 K, $CDCl_3$) δ = 144.25, 83.86, 25.03; **HRMS** (ESI$^+$): Calcd. for $C_{24}H_{39}B_3O_6H^+$: 457.3111, found 457.3109 $[M+H]^+$.

Data matches that previously reported. [2]

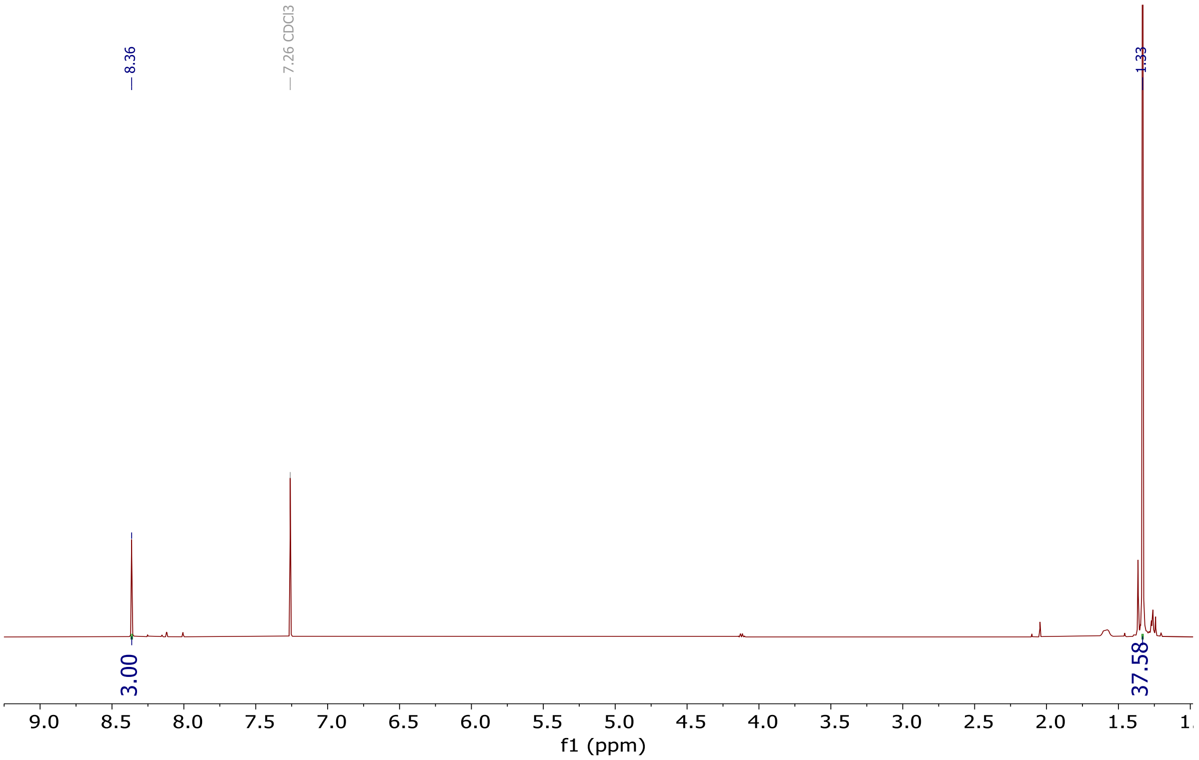

Figure S26: ^{1}H NMR (500 MHz, $CDCl_3$, 298 K) spectrum of **C2** between 1.0 and 9.0 ppm.

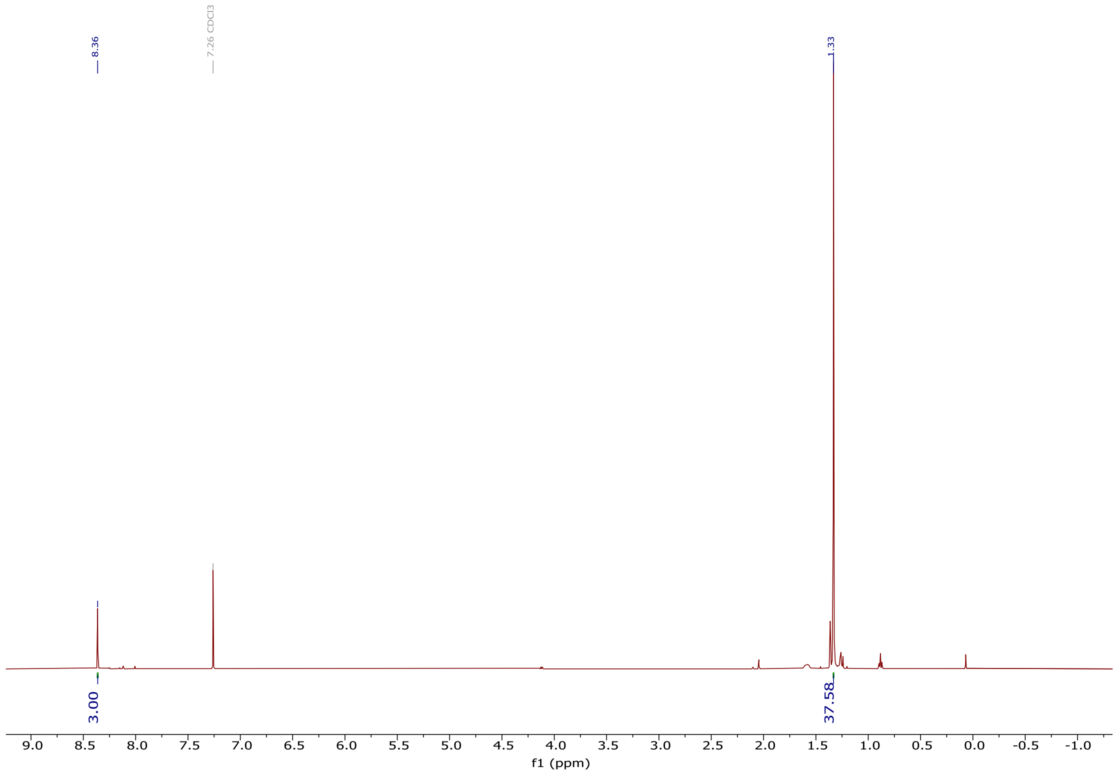

Figure S27: ^{1}H NMR (500 MHz, $CDCl_3$, 298 K) spectrum of **C2**.

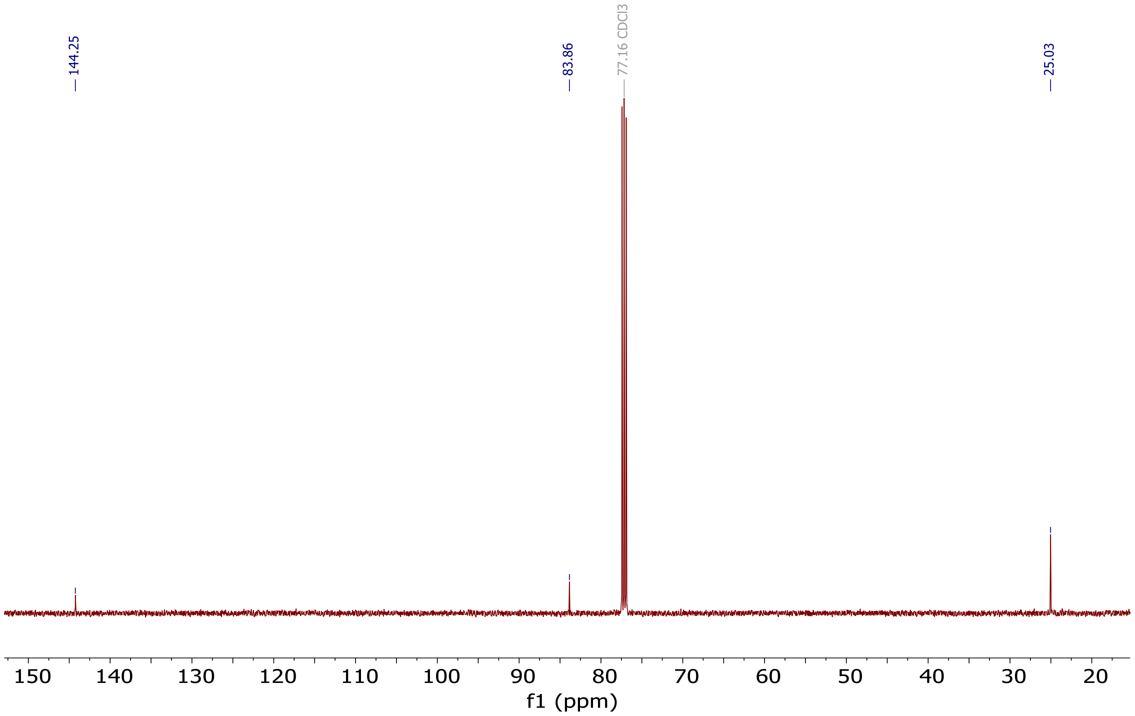

Figure S28: ^{13}C NMR (126 MHz, $CDCl_3$, 298 K) spectrum of **C2** between 20 and 150 ppm.

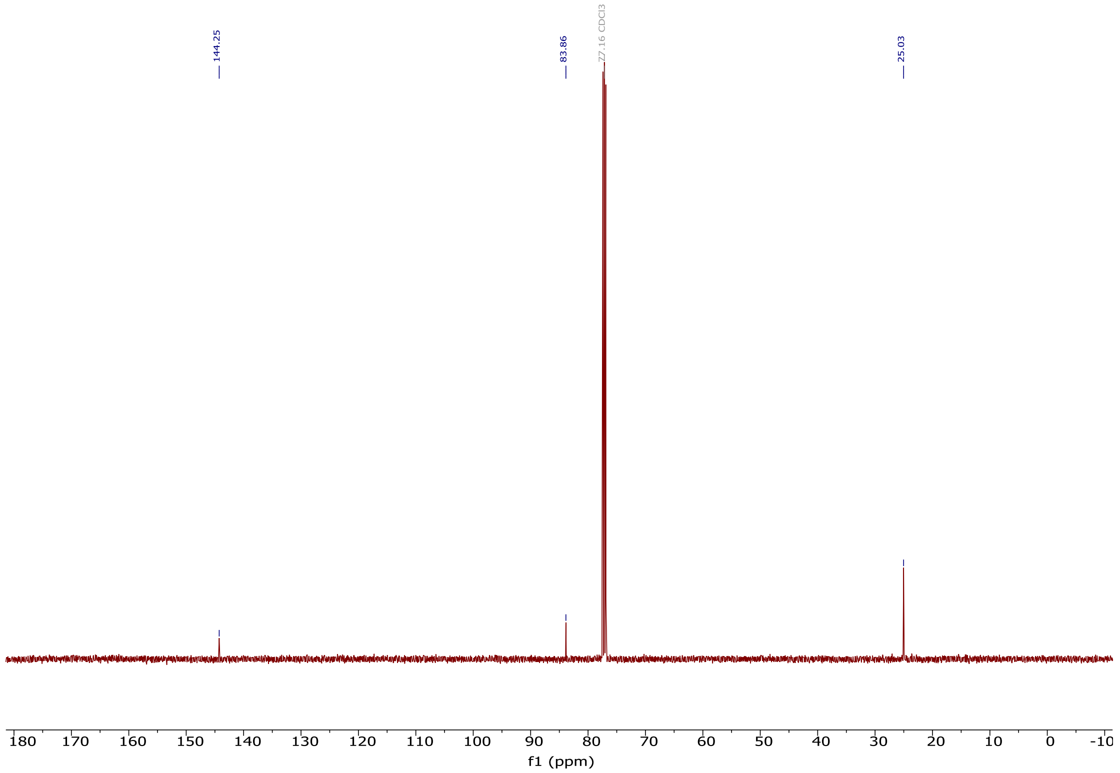

Figure S29: ^{13}C NMR (126 MHz, $CDCl_3$, 298 K) spectrum of **C2**.

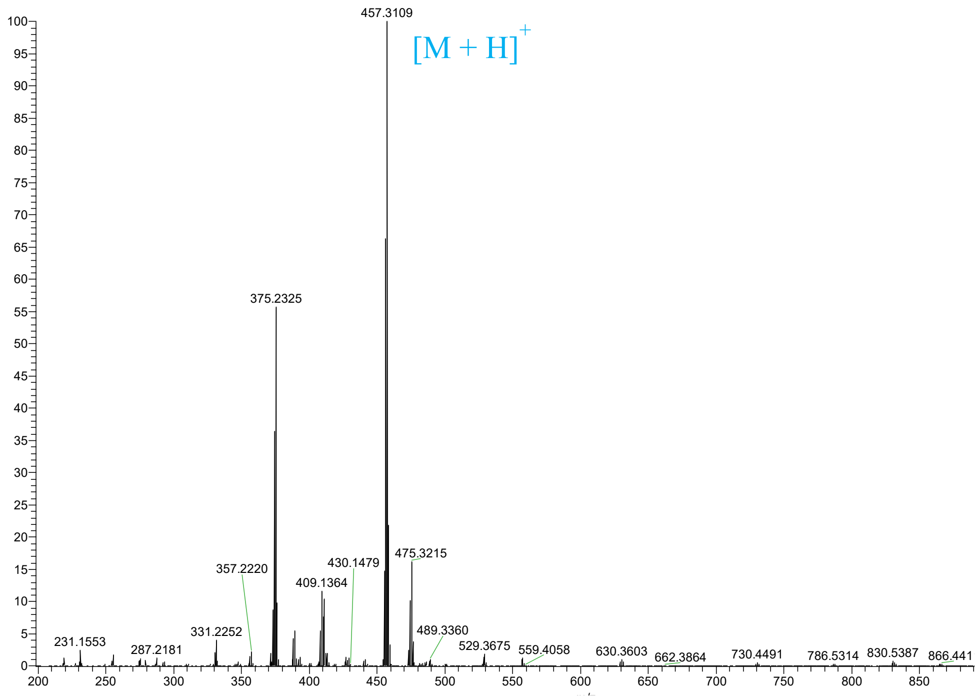

Figure S30: High resolution mass spectrum for **C2**: 457.3109 $[M+H]^+$.

Dimethyl 3,3''-diamino-5'-(3-amino-4-(methoxycarbonyl) phenyl)-[1,1':3',1''-terphenyl]-4,4''-dicarboxylate (C3):

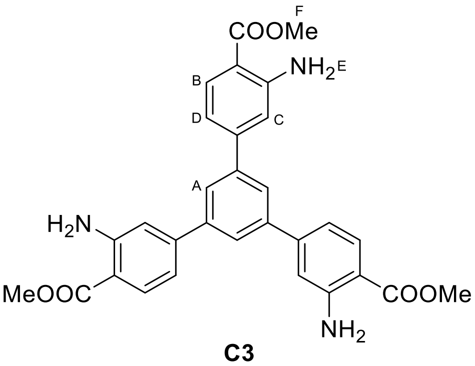

C3

C2 (0.79 g, 1.7 mmol) and methyl 2-amino-4-bromobenzoate (1.35 g, 5.88 mmol) were dissolved in 48 mL mixed solvent of *p*-dioxane/H_2O (1:1 v/v). The mixture was then deoxygenated by three freeze-pump-thaw cycles and protected under a N_2 atmosphere. CsF (2.40 g, 15.7 mmol) and Pd(dppf)Cl_2 (0.095 g, 0.13 mmol) were quickly added to the reaction mixture, the suspension was then heated and stirred at 90°C for 24 hours. After cooling the flask to room temperature, 150 mL of 20% NH_4Cl solution was added to the suspension. The suspension was later extracted three times with 3 × 50 mL EtOAc using a 250 mL separatory funnel. Combined organic layers were washed with saturated brine and dried with Na_2SO_4 followed by filtration over celite. The crude product was obtained after removing all the solvent under vacuum, and further purified by flash column chromatography using CH_2Cl_2/EtOAc (15:1) yielding **C3** as an off-white solid (457 mg, 48% yield). **^{1}H NMR** (500 MHz, 298 K, DMSO-d_6) δ 7.85 (s, 3H, H^A), 7.83 (d, J = 8.4 Hz, 3H, H^B), 7.24 (d, J = 1.9 Hz, 3H, H^C), 7.00 (dd, J = 8.4, 1.8 Hz, 3H, H^D), 6.74 (s, 6H, H^E),3.82 (s,9H, H^F); **^{13}C NMR** (126 MHz, 298 K, DMSO-d^6) δ = 167.66, 151.64, 144.89, 140.89, 131.47, 124.79, 114.62, 113.82, 108.28, 67.03 (THF impurity), 51.50; **HRMS** (ESI$^+$): Calcd. For $C_{30}H_{27}N_3O_6Cl$: 560.1594, found 560.1577 [M+Cl].

Data matches that previously reported. [2]

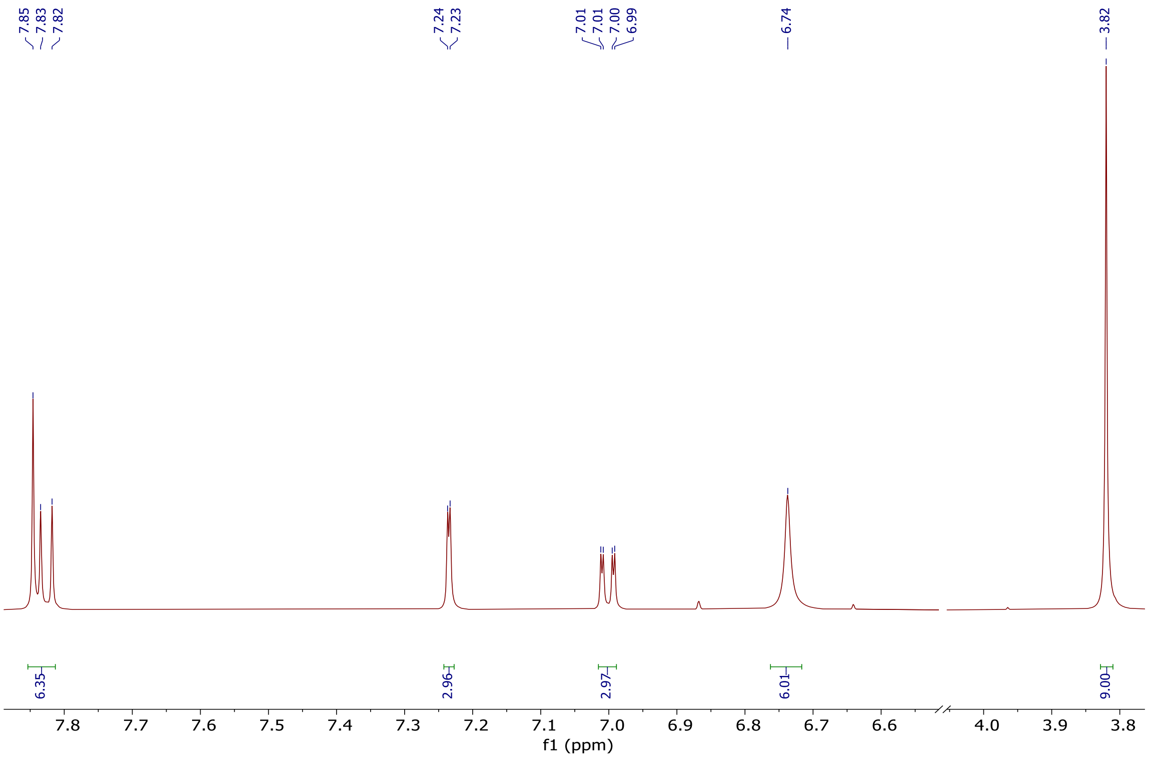

Figure S31: ^{1}H NMR (500 MHz, DMSO-d_6, 298 K) spectrum of **C3** between 3.8 and 7.9 ppm.

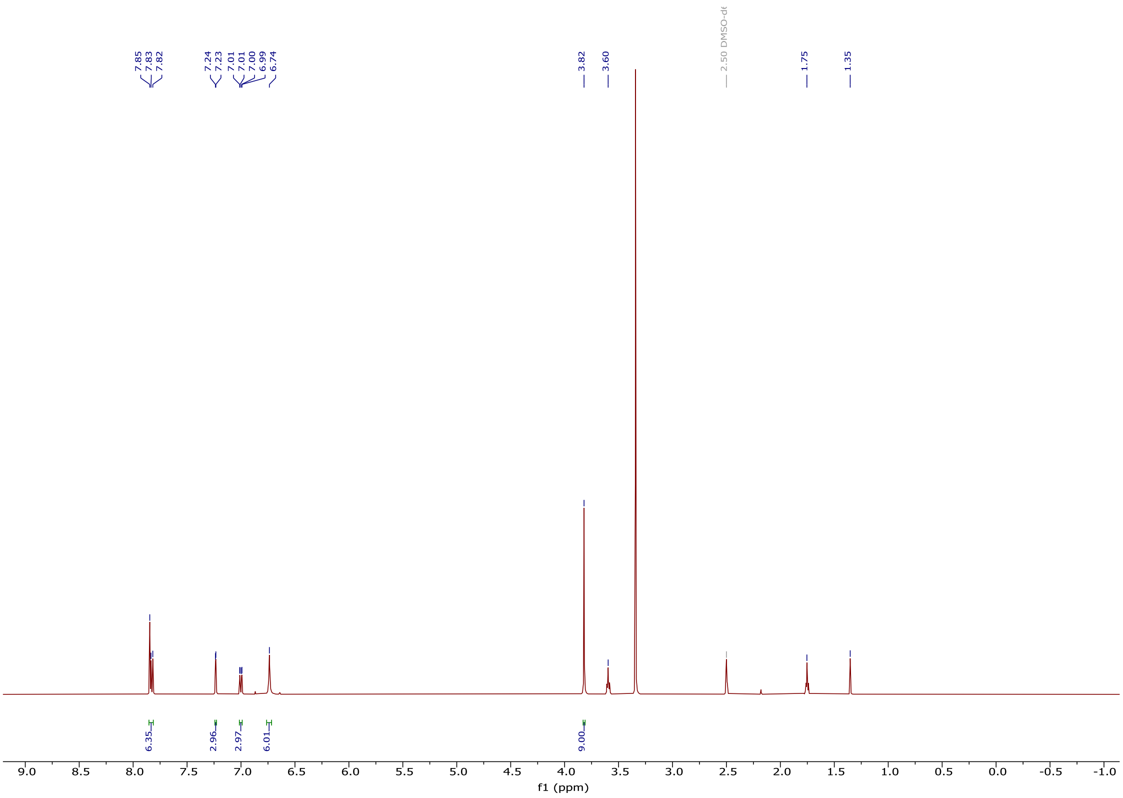

Figure S32: ^{1}H NMR (500 MHz, DMSO-*d*$_6$, 298 K) spectrum of **C3**.

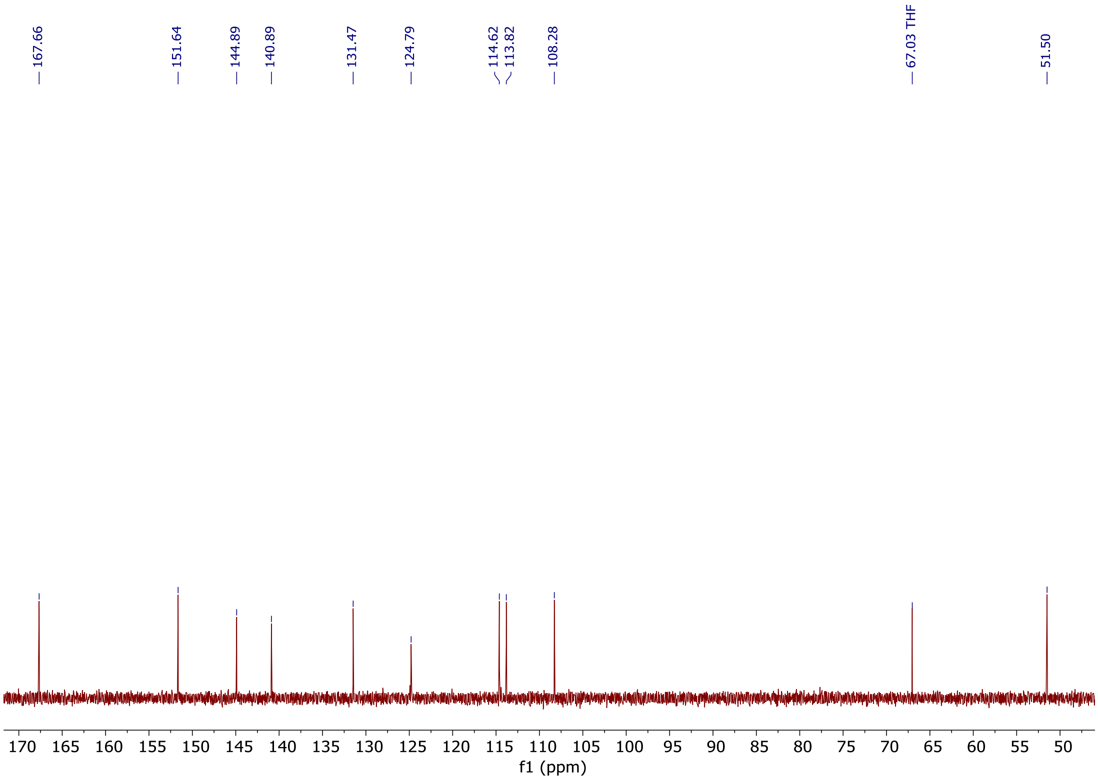

Figure S33: ^{13}C NMR (126 MHz, DMSO-*d*$_6$, 298 K) spectrum of **C3** between 50 and 170 ppm.

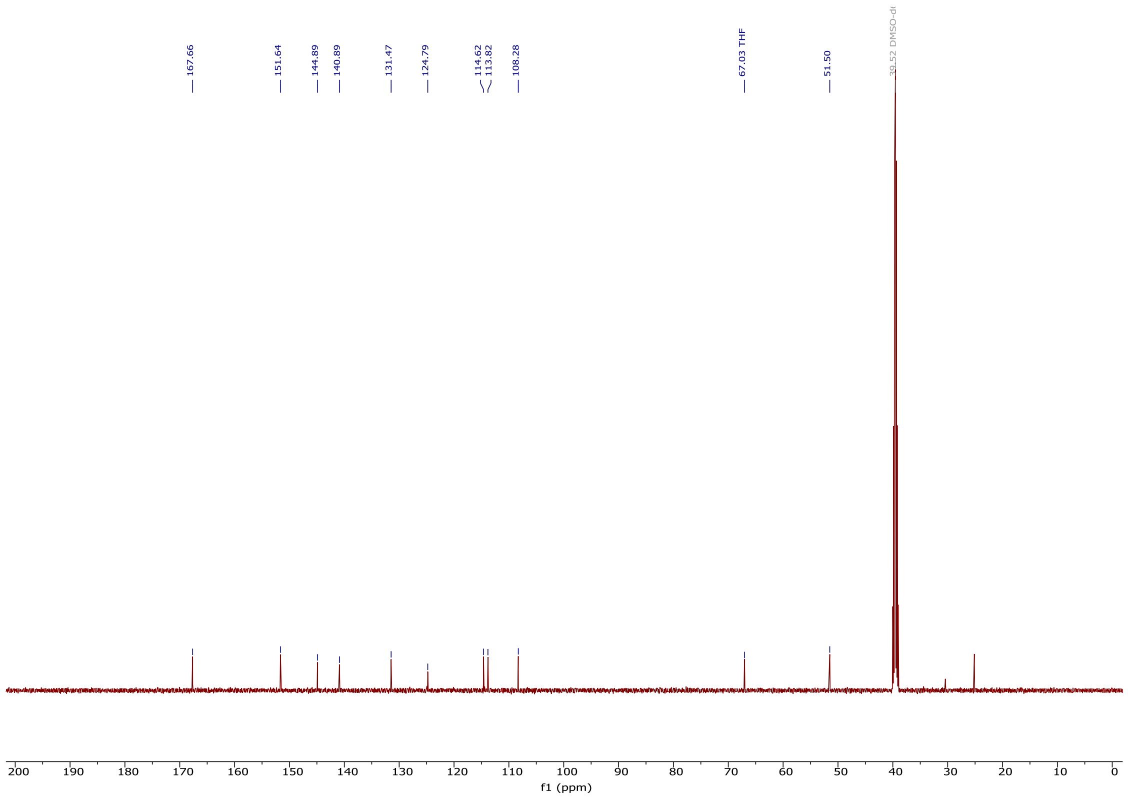

Figure S34: ^{13}C NMR (126 MHz, DMSO-d_6, 298 K) spectrum of **C3**.

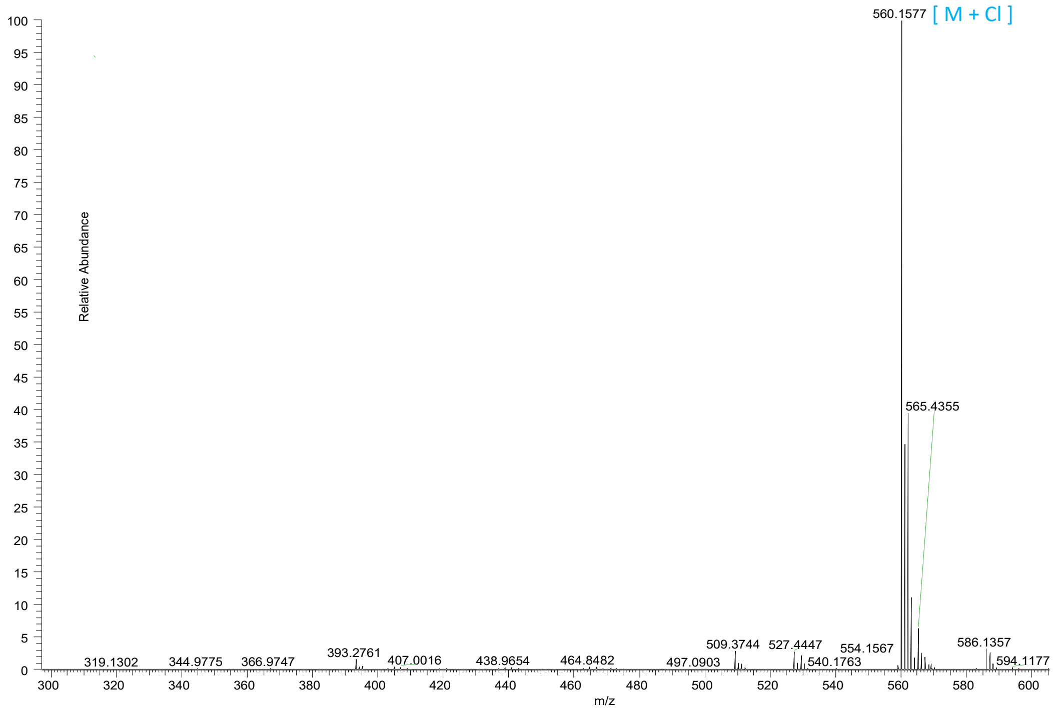

Figure S35: High-resolution mass spectrum for **C3:** 560.1577 [M+Cl]$^-$.

3,3''-Diamino-5'-(3-amino-4-carboxyphenyl)-[1,1':3',1''-terphenyl]-4,4''-dicarboxylic acid (BTB-NH₂):

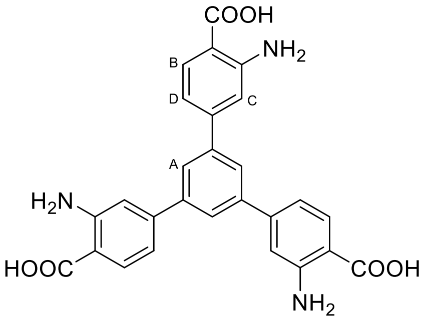

BTB-NH$_2$

BTB-NH$_2$ (0.443 mg, 0.840 mmol) was dissolved in 27 mL THF, and 0.5 M NaOH aqueous solution (27 mL, 13.5 mmol) was added. The suspension was stirred and heated at 50 ºC for 48 hours. After carefully removing THF and MeOH by rotatory evaporation, the aqueous solution was acidified with concentrated HCl to pH < 4. The yellow paste was collected by filtration, washed with DI water and dried under vacuum to obtain **BTB-NH$_2$** as a yellow solid (88% yield). **^{1}H NMR** (500 MHz, 298 K, DMSO-*d$_6$*) δ = 7.90 (s, 3H, H^A), 7.88 (d, J=8,3 Hz, 3H, H^B), 7.27 (d, *J* = 1.9 Hz, 3H, H^C), 7.05 (dd, *J* = 8.3, 1.8 Hz, 3H, H^D); **^{13}C NMR** (126 MHz, 298 K, DMSO-*d$_6$*) δ = 169.34, 151.57, 144.70, 141.00, 132.03, 124.69, 114.56, 113.81, 109.38. **HRMS** (ESI$^+$): Calcd. For $C_{27}H_{20}N_3O_6$:482.1358, found 482.1336 [M-H]$^-$.

Data matches that previously reported.[2]

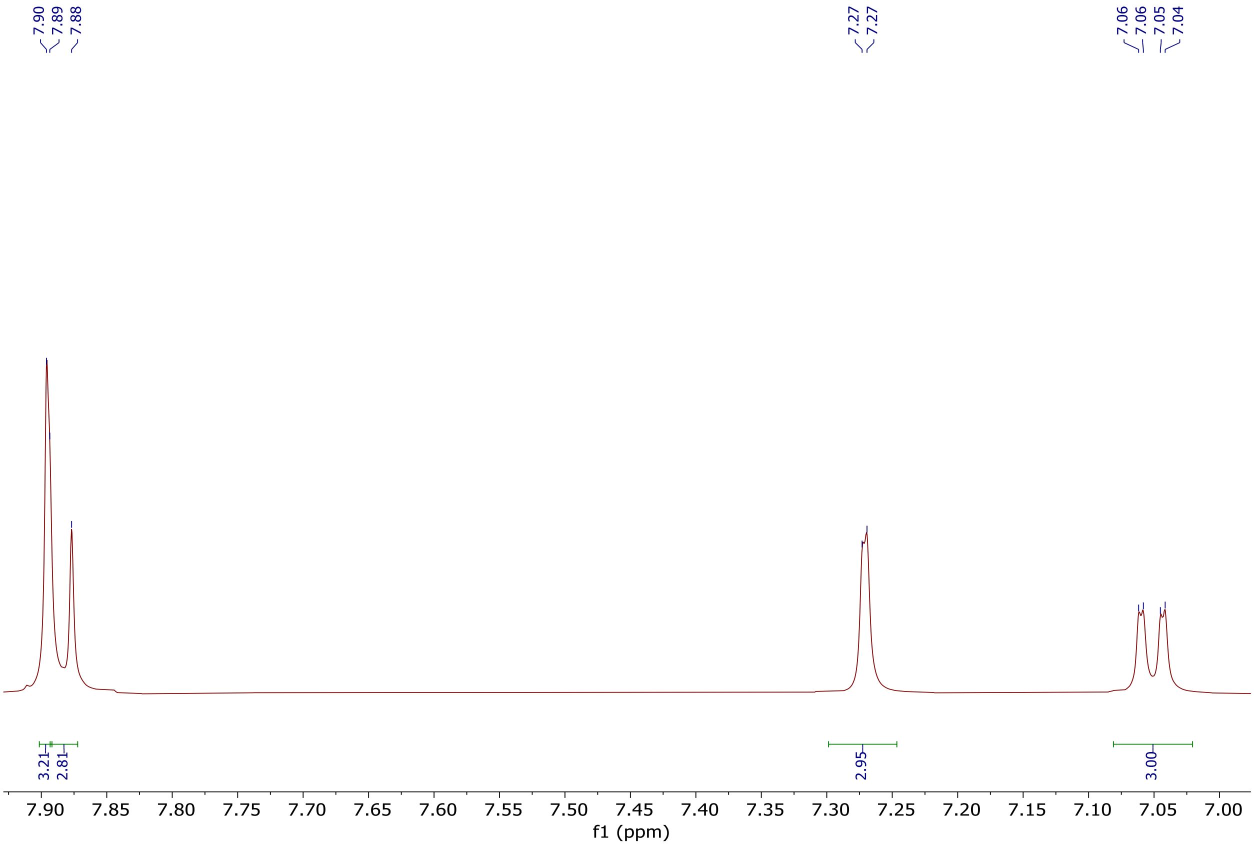

Figure S36: ^{1}H NMR (500 MHz, DMSO-*d$_6$*, 298 K) spectrum of **BTB-NH$_2$** between 7.0 and 7.95 ppm.

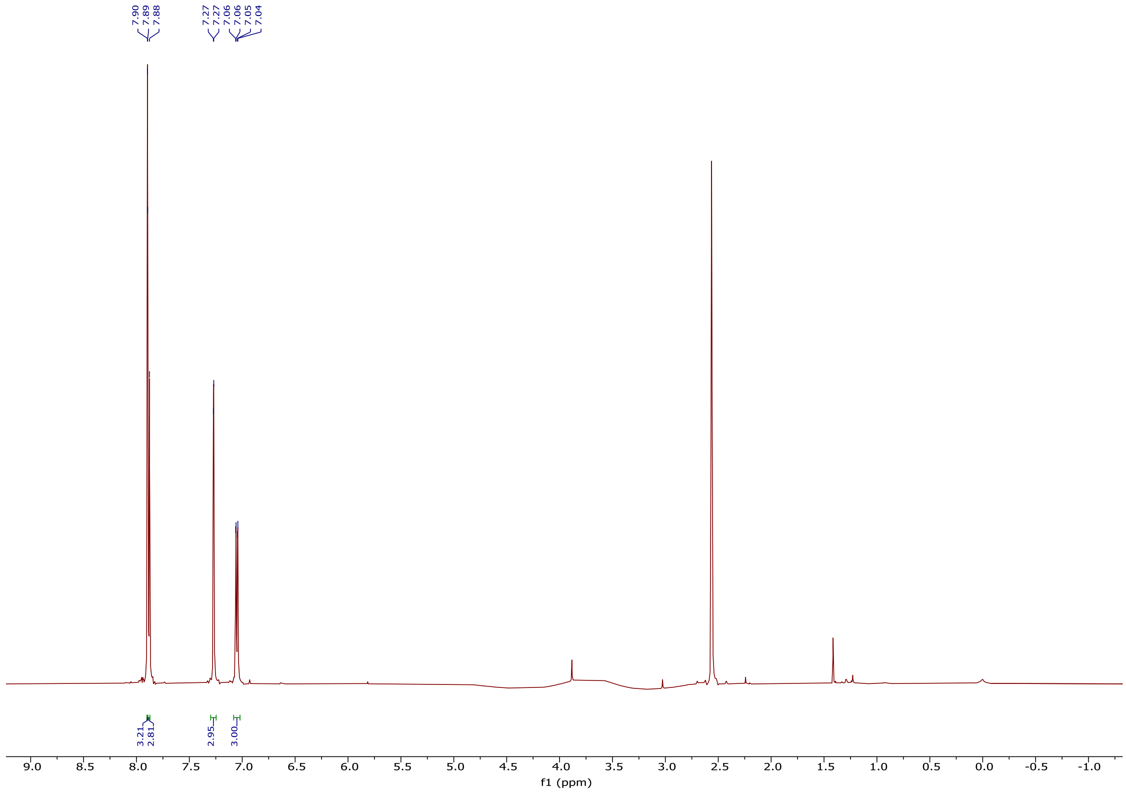

Figure S37: ^{1}H NMR (500 MHz, DMSO-d_6, 298 K) spectrum of **BTB-NH₂**.

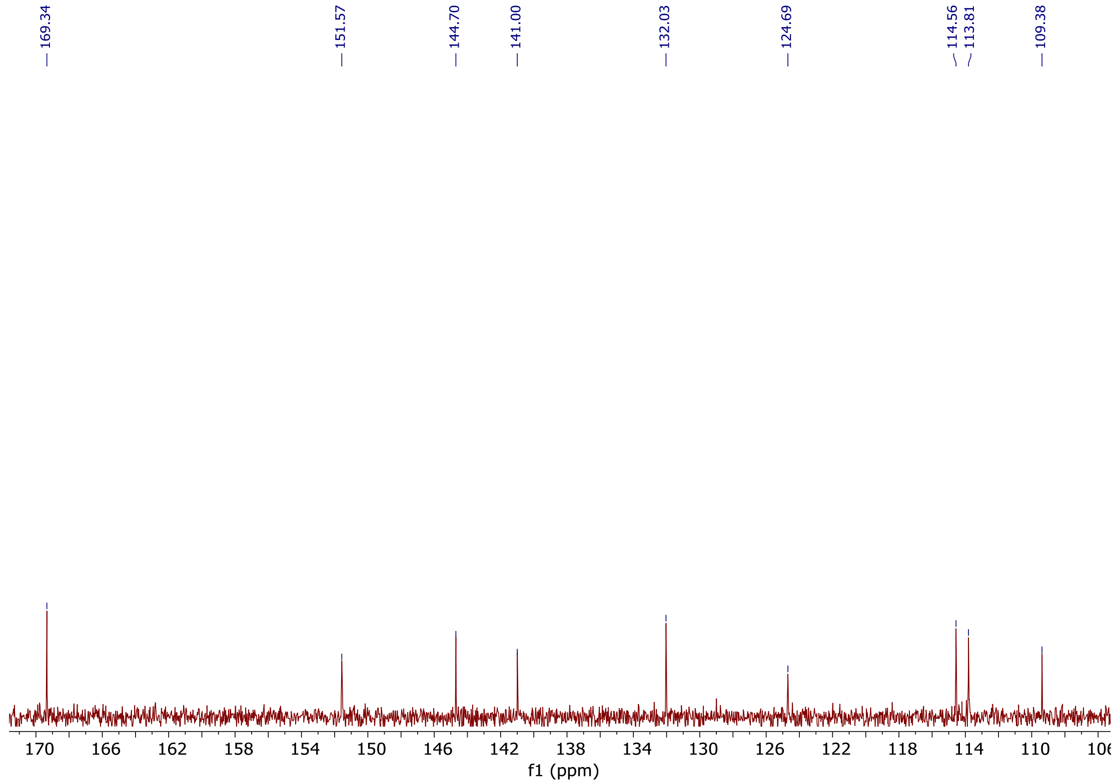

Figure S38: ^{13}C NMR (126 MHz, DMSO-d_6, 298 K) spectrum of **BTB-NH$_2$** between 106 and 170 ppm.

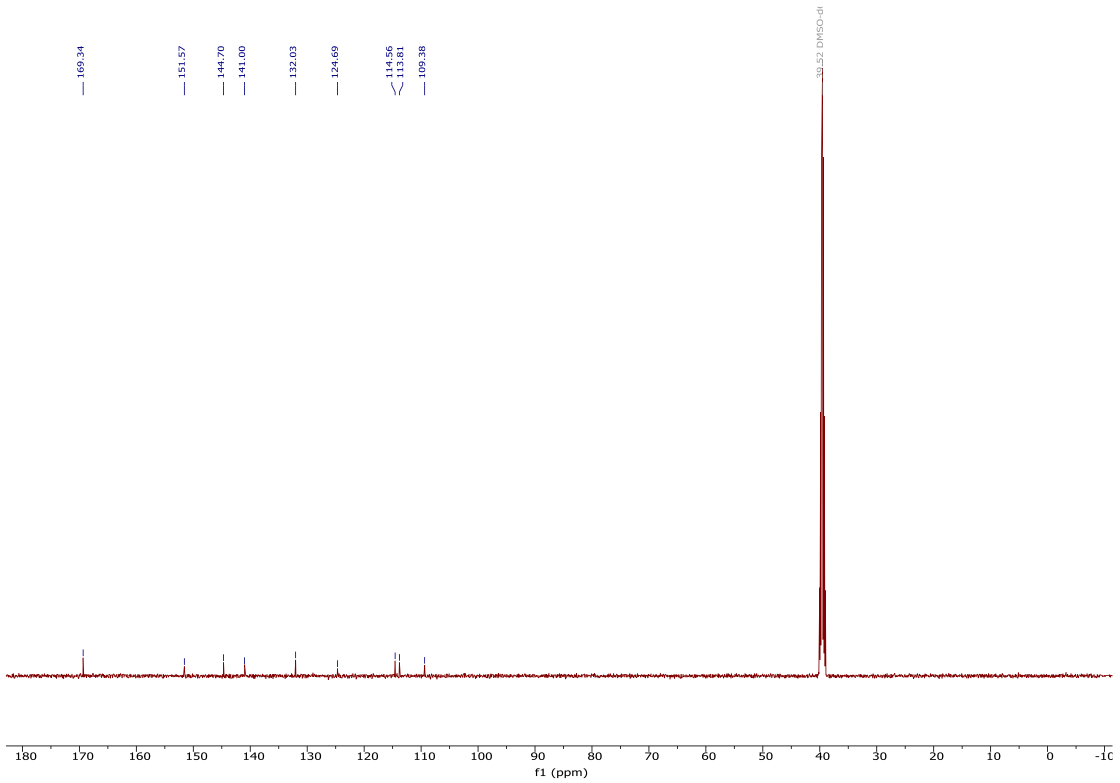

Figure S39: ^{13}C NMR (126 MHz, DMSO-d_6, 298 K) spectrum of **BTB-NH$_2$**.

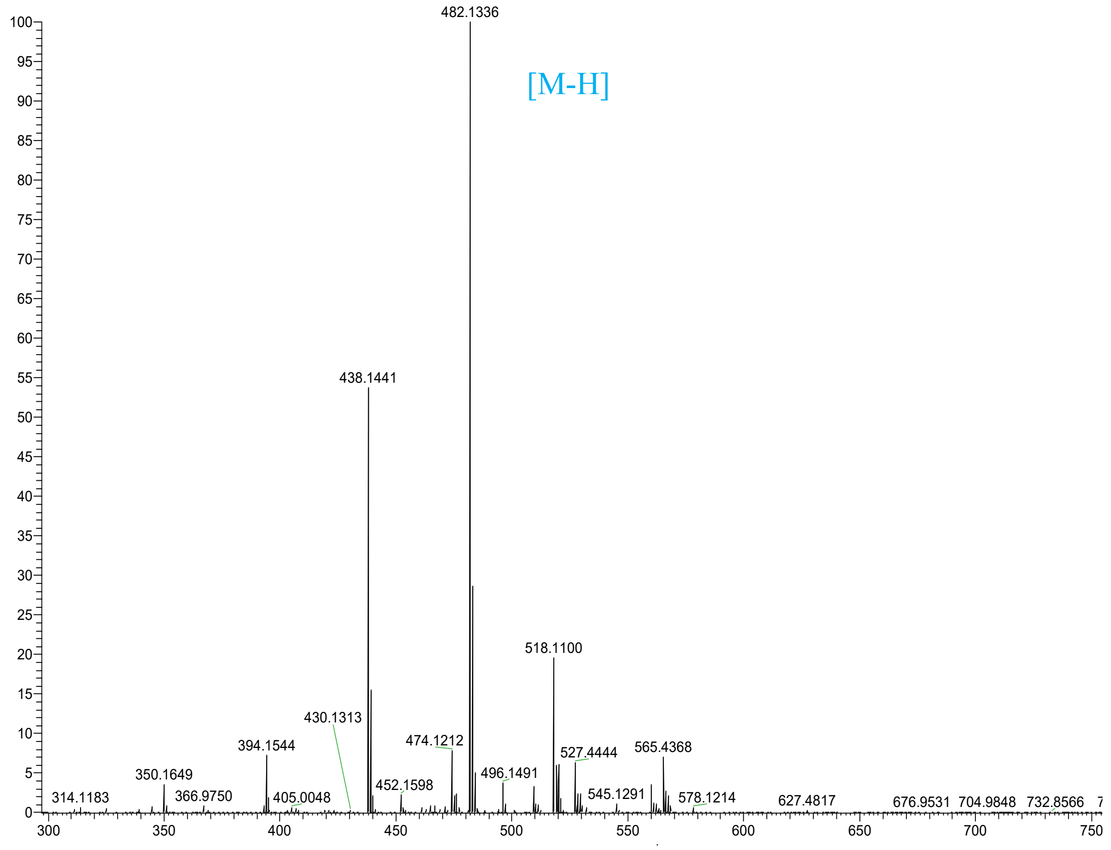

Figure S40: High-resolution mass spectrum for **BTB-NH$_2$**: 480.1336 [M-H]$^-$.

S2 Synthesis of Protein@MOFs by biomimetic mineralisation.

S2.1 BSA@ZnBDC-R (where R= -H, -OH, $-CH_3$, NH_2, Br)

Terephthalic acid (0.25 mmol) or other derivative ligands were deprotonated using sodium hydroxide solution (1M) and transferred into 10.0 mL DI water. The pH of the solution was adjusted to the pH 7.0 with NaOH (1 M) and HCl (1 M). BSA (5.0 mg) was added directly to the ligand solution. Separately, a zinc(II)nitrate solution was prepared by dissolving zinc(II)nitrate hexahydrate(0.25 mmol) in 10.0 mL DI water. The ligand solution and Zn(II) solution were then mixed with continuous stirring for 48 hours. Any precipitate which formed was collected by centrifugation and washed with DI water (2 × 10.0 mL) and with methanol (1 × 10.0 mL). The white powder isolated was dried at room temperature for 24 hours and characterised as outlined below (Section S3).

S2.2 ZnBDC-NH_2 I and HRP@ZnBDC-NH_2 I

Terephthalic acid (0.25 mmol) or other derivative ligands were deprotonated using sodium hydroxide solution (1 M) and transferred into 10.0 mL DI water. The pH of the solution was adjusted to 7.0 using NaOH (1 M) and HCl (1 M). HRP (2.5 mg) was added directly to the ligand solution. Separately, a zinc(II) nitrate solution was prepared by dissolving zinc(II) nitrate hexahydrate (0.75 mmol) in 10.0 mL DI water. The ligand solution and Zn(II) solution were then mixed with continuous stirring for 48 hours. Any precipitate which formed was collected by centrifugation, washed with DI water (2 × 10.0 mL) and with methanol (1 × 10.0 mL). The white powder isolated was dried at room temperature for 24 hrs and characterised as outlined below (Section S3).

S2.3 ZnBDC-NH_2 II and HRP@ZnBDC-NH_2 II

2-Aminoterephthalic acid (90.58 mg, 0.5 mmol) in DI water was deprotonated using sodium hydroxide solution (1 M) and diluted with DI water to make final volume to 20 mL. After that, 2.5 mg HRP was dissolved into the ligand solution. Zinc nitrate hexahydrate (446.22 mg, 1.5 mmol) was dissolved into 20.0 mL DI water to make the Zn(II) solution. The ligand solution was mixed with the Zn (II) solution and the reaction mixture was stirred for 24 hours. The white precipitate was washed with DI water and methanol twice, respectively. The ZnBDC-NH_2 II was synthesized according to the same synthetic procedure as HRP@ZnBDC-NH_2 II, except the synthesis was performed in the absence of HRP.

S2.4 HRP@ZnBPDC-NH_2 and ZnBPDC-NH_2

A solution of BPDC-NH_2 (27.25 mg, 0.1 mmol) in DI water was deprotonated using NaOH (1 M) and made up to a final volume of 10.0 mL. HRP (1.0 mg) was then added to the ligand solution (29.75 mg, 0.1 mmol). A separate solution of zinc(II) nitrate hexahydrate (29.74 mg, 0.1 mmol) was also prepared in 10.0 mL DI. The ligand solution was mixed with the Zn(II) solution and the reaction was stirred for 2 hours. The resultant white precipitate was collected and washed with DI water and methanol twice.

ZnBPDC-NH_2 was synthesized according to the same synthetic procedure as HRP@ZnBPDC-NH_2, except the synthesis was performed in the absence of HRP.

S2.5 HRP@ZnTPDC-NH_2 and ZnTPDC-NH_2

A solution of TPDC-NH_2 (34.83 mg, 0.1 mmol) in DI water was deprotonated using NaOH (1 M) and made up to a final volume to 10.0 mL. HRP (1.0 mg) was then added to the ligand solution. Zinc(II) nitrate hexahydrate (29.75 mg, 0.1 mmol) was dissolved in 10.0 mL DI water to make the Zn(II) solution. The ligand solution was then mixed with the Zn(II) solution and stirred continuously for 2 hours after which time the white precipitate which had formed was collected, washed with DI water and methanol twice and dried.

ZnTPDC-NH_2 was synthesized according to the same synthetic procedure as HRP@ZnTPDC-NH_2, except the synthesis was performed in the absence of HRP.

S2.6 Attempted synthesis of HRP@ZnBTB-NH_2

BTB-NH_2 (48.3 mg, 0.1 mmol) was dissolved into 20.0 mL DI water with 0.5 mL NaOH solution (1 M). The mixture was heated and stirred at 90 °C for 3 hours until a clear solution was obtained. The insoluble impurity was removed by filtration, and the pH of the solution was adjusted to 9.0 with HCl solution (1 M). HRP (1.0 mg) was then dissolved into the BTB-NH_2 ligand solution. Zinc(II) nitrate hexahydrate (297.5 mg, 1.0 mmol) was dissolved into 20.0 mL DI water to prepare the Zn (II) solution. The ligand solution was mixed with Zn(II) solution and a white precipitate formed immediately. The precipitate was collected by centrifugation and washed with DI water twice and methanol. The final product was dried at room temperature before characterisation.

S3 Characterisation of Protein@MOFs

S3.1 Characterisation methods

Powder X-ray Diffraction (PXRD): PXRD characterisation was performed with a Philips X'Pert MPD X-ray diffractometer equipped with a Philips high-intensity ceramic sealed tube (3 kW) with a Cu anode X-ray source. The operating power was set as 40 kV, 45 mA. The data was processed using PowDLL Converter and phase analysis was performed with X'Pert HighScore Plus. Peak finding and indexing were performed using Topas6.[3] Outputted candidate unit cells were searched against the Cambridge Structure Database to find the corresponding structure.[4] Le Bail profile fit analysis for the found unit cells were performed in Jana2020.[5] Idealised calculated patterns were generated from the structure files as obtained from the CCDC using Mercury,[6] with a FWHM of 0.2 and inclusion of Cu $K\alpha 1/K\alpha_2$ radiation in a 0.5 ratio.

Fourier-transform Infrared Spectroscopy (FTIR): FTIR characterisation was performed on Bruker FTIR Alpha Spectrometer.

Elemental analysis: C, H, N & S analysis was performed on a Thermo CFlash 2000 Elemental Analyser. ICP-OES was employed for the quantification of iron and zinc using a Thermo Scientific iCAP 6300 Duo.

Thermogravimetric analysis (TGA): TGA was performed on a Mettler- Toledo STARe instrument. Sample mass losses were measured between 25 and 650°C with heating perfomed under a constant nitrogen gas stream.

Scanning electron microscope (SEM) and Energy-Dispersive X-ray spectroscopy (EDX): The HRP@MOFs and MOFs samples were dried by a LABCONCO FreeZone 2.5 L-84C Benchtop Freeze Dryer for 24 hours prior to their attachment on aluminium stubs using conductive carbon tape. The samples were coated with platinum before analysis. The morphology and the surface elemental mapping spectra of the HRP@MOFs and MOFs were visualised by FEI Quanta 250 FEG-SEM + Gatan 3view.

Confocal fluorescence microscope: Fluorescence confocal spectra were collected using a Leica SP8 Upright Laser Confocal Microscope with a 20× objective lens. The wavelength of the excitation laser was set as 495 nm, and the emission was collected between 500 – 580 nm. The Pinhole was set as 1.0.

Raman Spectroscopy: Raman spectra were collected by RENISHAW inVia™ InSpect confocal Raman microscope with a 785 nm laser source and a 50× objective lens. Exposure time was used as 1 second, the laser power was set as 10% and accumulation was chosen as 200.

S3.2 Protein Quantification

Bradford Assay[7]: To measure the protein concentration in the supernatant following biomimetic crystallisation the standard Bradford assay parameters were modified, to generate a calibration plot with data points between 1- 100 μg/mL.

Before each assay, the dye reagent was allowed to warm to room temperature. A standard solution of BSA (2 mg/mL) was diluted to a range of concentrations (100 μg/mL, 50 μg/mL, 25 μg/mL, 12.5 μg/mL, 10 μg/mL 5 μg/mL, 1 μg/mL) and added individually to wells of a 96-well plate. Each of the BSA solutions and the experimental samples were mixed with 5 μL of Coomassie protein assay reagent. The absorbance at 595 nm for each well was measured and the protein concentration of the experimental samples was determined from the calibration curve.

Encapsulation efficiency (EE%) and Loading Content (LC%):

EE and LC for each sample were calculated as previously detailed in the literature.[8]

$$\text{Encapsulation efficiency (EE\%)} = \frac{m_{(HRP)} - CV}{m_{(HRP)}} \times 100\%$$

Equation S1: Calculation of encapsulation efficiency; where $m_{(HRP)}$(mg) = total HRP amount, C(mg/mL) = protein concentration in the supernatant, V(mL) = supernatant volume.

$$\text{Loading content (LC\%)} = \frac{m_{(HRP\ in\ HRP@MOFs)}}{m_{(HRP@MOFs)}} \times 100\%$$

Equation S2: Calculation of loading content; where $m_{(HRP\ in\ HRP@MOFs)}$ (mg) = the amount of HRP loaded in MOFs, $m_{(HRP@MOFs)}$ = the amount of HRP@MOFs

S3.3 Evaluation of BSA@ZnBDC-R Formation (where R= -H, -OH, $-CH_3$, NH_2, Br)

	Reaction Time (hr)							
	0.5	1	2	4	8	12	24	36
terephthalic acid (-H)								neg.
2-hydroxyterephthalic acid (-OH)	No precipitate							
2-methylterephthalic acid (-Me)	No precipitate							
2-aminoterephthalic acid ($-NH_2$)	116.3 (78%)	142.5 (95%)	150.2 (100%)	141.3 (94%)	147.9 (99%)	167.4 (112%)	144.6 (97%)	154.7 (103%)
2-bromoterephthalic acid (-Br)	Amorphous precipitate							

Table S1: Weight of precipitate formed in mg from functionalised BDC ligands coordinating with zinc(II) ions (M:L 1:1) in the presence of BSA (10.0 wt %). Crystalline precipitate was only recovered from the time points shaded green. Neg indicates negligible precipitate was collected; % mass indicated in brackets corresponds to a maximum final yield. The final yield was an average value calculated from the mass of product collected at timepoints between 1 hr and 36 hrs. After approximately one hour no increase in mass was observed for 2-aminoterephthalic acid and it was deduced the reaction was complete.

S3.4 Characterisation of protein@ZnBDC-NH$_2$

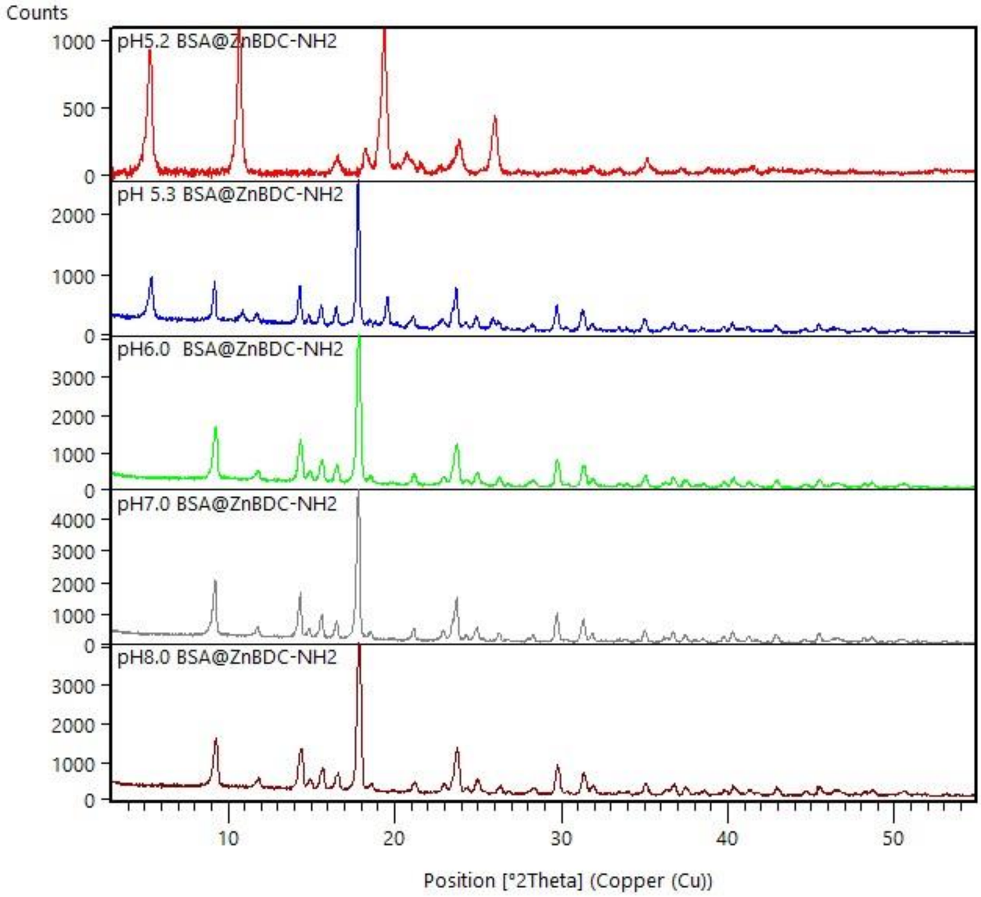

Figure S41: PXRD patterns of BSA@ZnBDC-NH$_2$ precipitate synthesized between pH 5.2 -8.0.

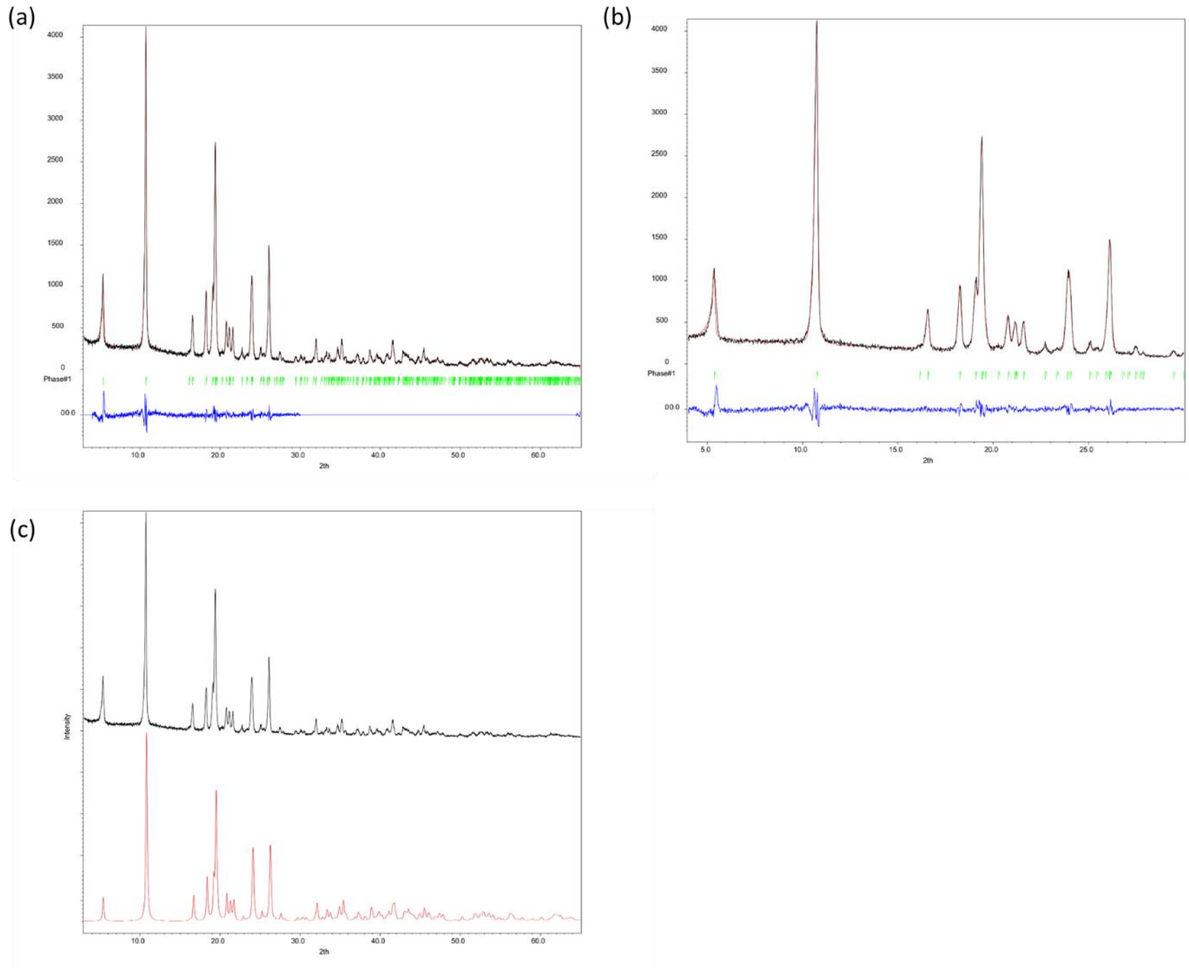

Figure S42. Le Bail profile refinement analysis for BSA@ZnBDC-NH_2 I. (a) Full range experimental data for BSA@ZnBDC-NH_2 II (black), fitted profile (red) and difference (blue) including tick marks for expected reflection positions (green); (b) experimental data range selected for Le Bail profile fitting analysis for BSA@ZnBDC-NH_2 I (black), fitted profile (red) and difference (blue) including tick marks for expected reflection positions (green); (c) full range experimental data for BSA@ZnBDC-NH_2 I (black) and simulated PXRD pattern from CCDC 1943053 (red).

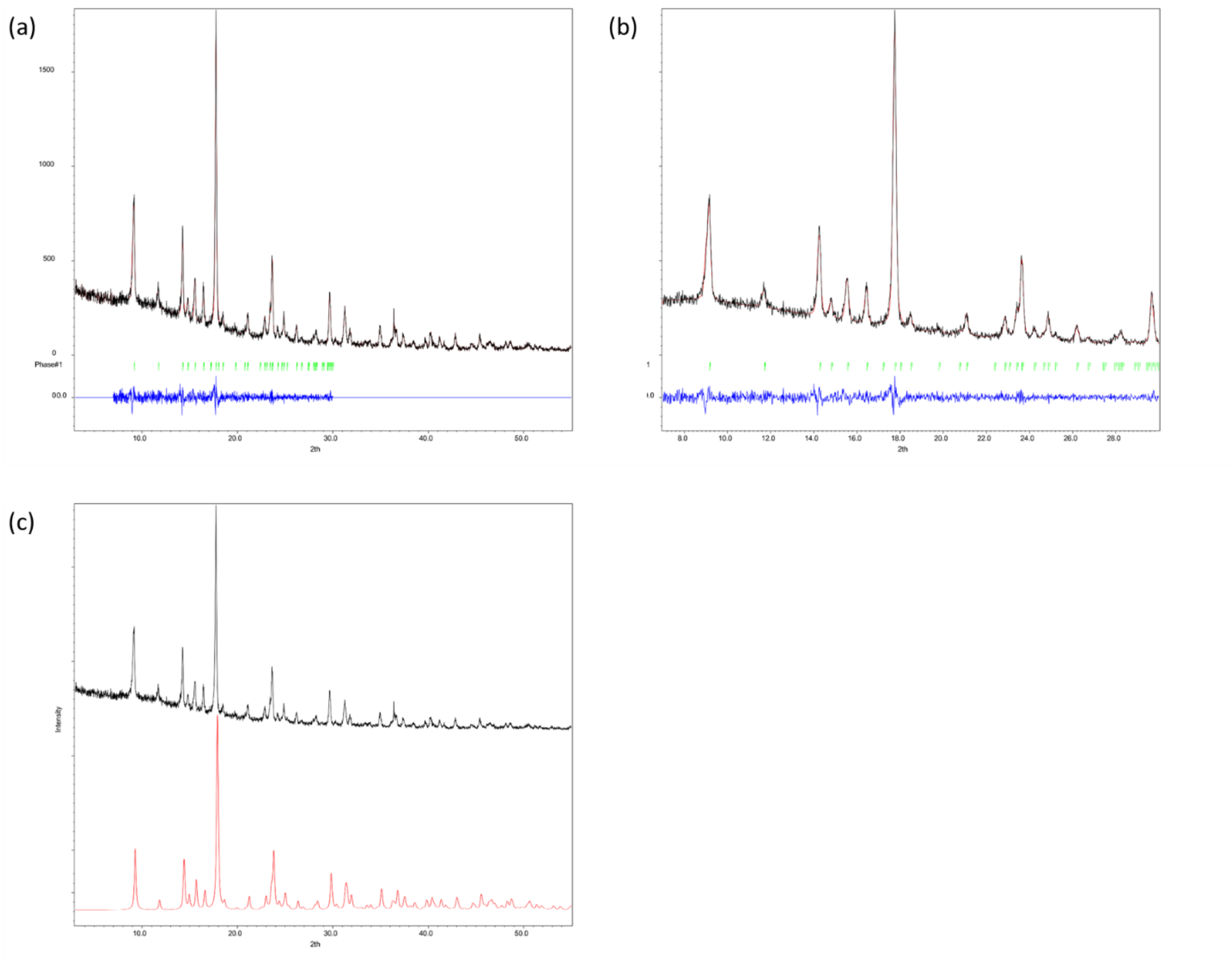

Figure S43. Le Bail profile refinement analysis for BSA@ZnBDC-NH_2 II. (a) Full range experimental data for BSA@ZnBDC-NH_2 II (black), fitted profile (red) and difference (blue) including tick marks for expected reflection positions (green); (b) experimental data range selected for Le Bail profile fitting analysis for BSA@ZnBDC-NH_2 II (black), fitted profile (red) and difference (blue) including tick marks for expected reflection positions (green); (c) full range experimental data for BSA@ZnBDC-NH_2 II (black) and simulated PXRD pattern from CCDC 607821 (red).

**Compound**	**a**	**b**	**c**	**α**	**β**	**γ**
**BSA@ZnBDC-NH_2 I**	33.124(4)	5.4165(4)	9.1827(9)	90	96.539(8)	90
**BSA@ZnBDC-NH_2 II**	9.578(2)	6.4686(13)	15.045(2)	90	95.031(9)	90

Table S2: Unit cell values obtained from Le Bail profile fitting results.

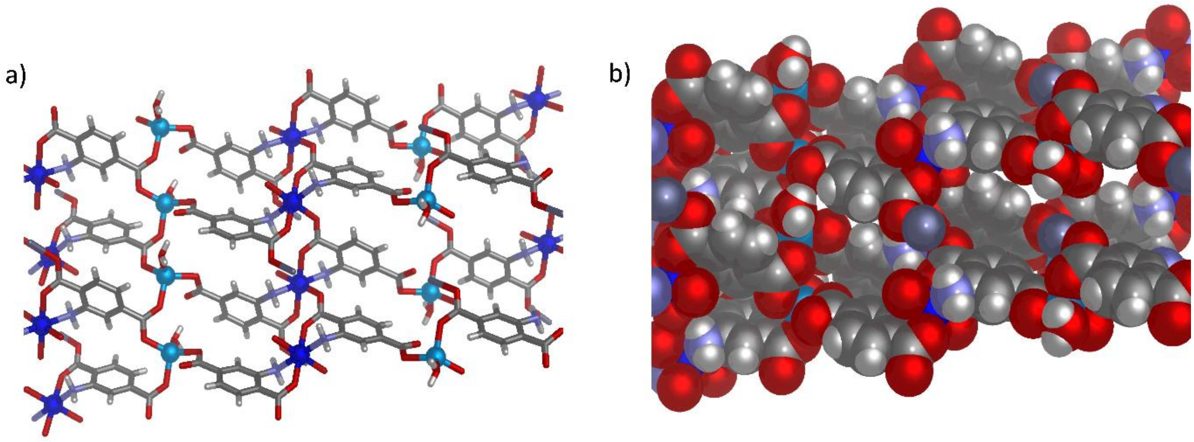

Figure S44: Structural illustration of BSA@ZnBDC-NH_2 I: (a) ball and stick style figure of crystal structure. (b) Space-filled representation of HRP@ZnBDC-NH_2 I (red: oxygen, grey: hydrogen, black: carbon, dark blue and light blue represent zinc(II) ions in differing coordination environments).

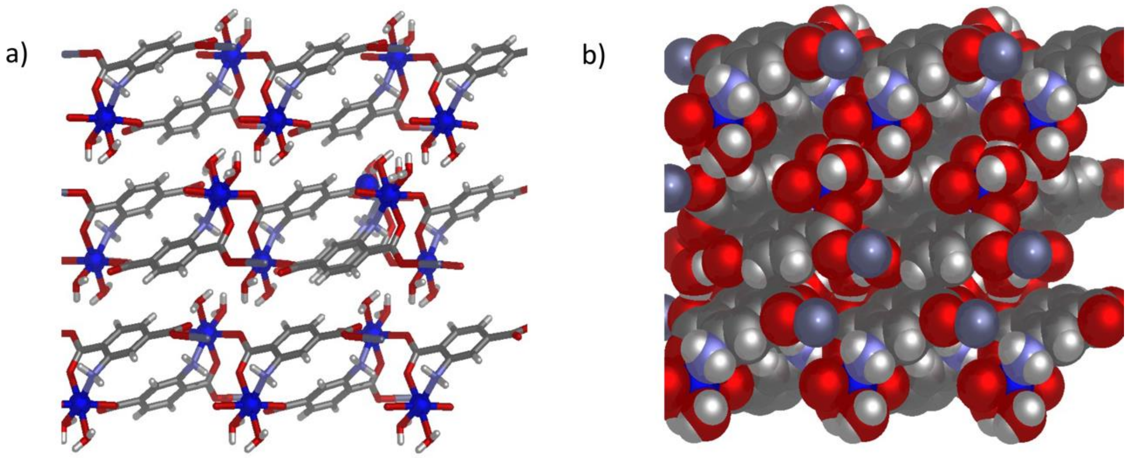

Figure S45: Structural illustration of HRP@ZnBDC-NH_2 II: (a) ball and stick style figure of crystal structure. (b) space-filled representation of crystal structure with water molecules (red: oxygen, grey: hydrogen, black: carbon, blue: zinc(II) ions).

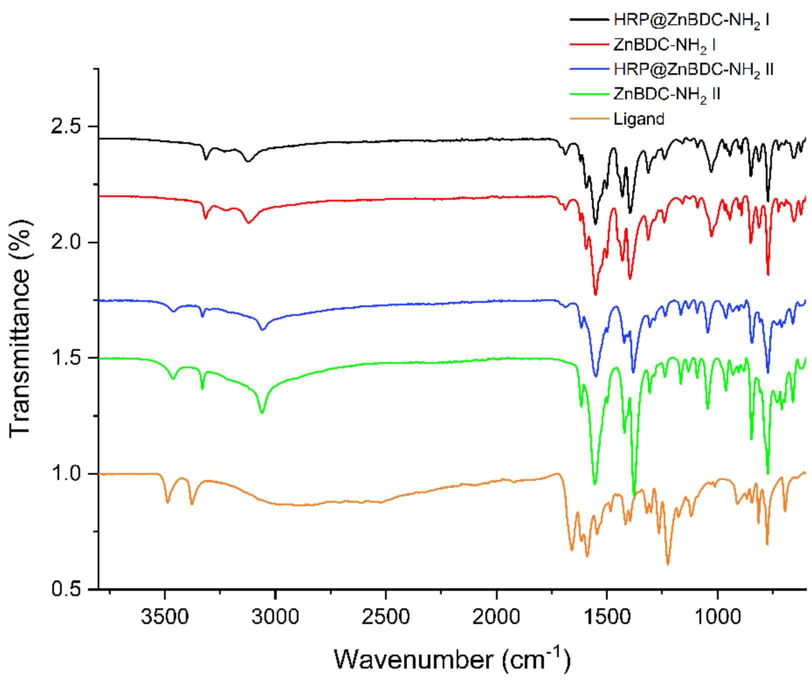

Figure S46: FTIR patterns of $ZnBDC-NH_2$ I in the presence(black) and absence(red) of HRP, $ZnBDC-NH_2$ II in the presence(blue) and absence(green) of HRP and the 2-aminoterephthalic acid ligand (orange).

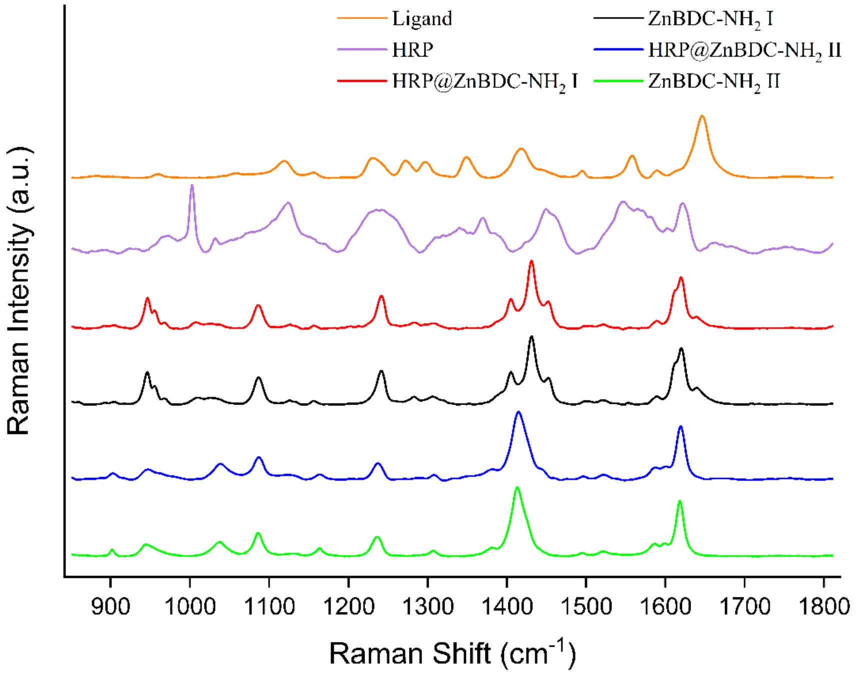

Figure S47: Raman spectra of HRP@$ZnBDC-NH_2$ I (black) & II (blue), $ZnBDC-NH_2$ I (red) & II (green) and HRP (purple) collected with 785 nm laser source. The absence of evidence for HRP encapsulation within MOFs is attributed to the low loading content of HRP and potential peak degeneracy.

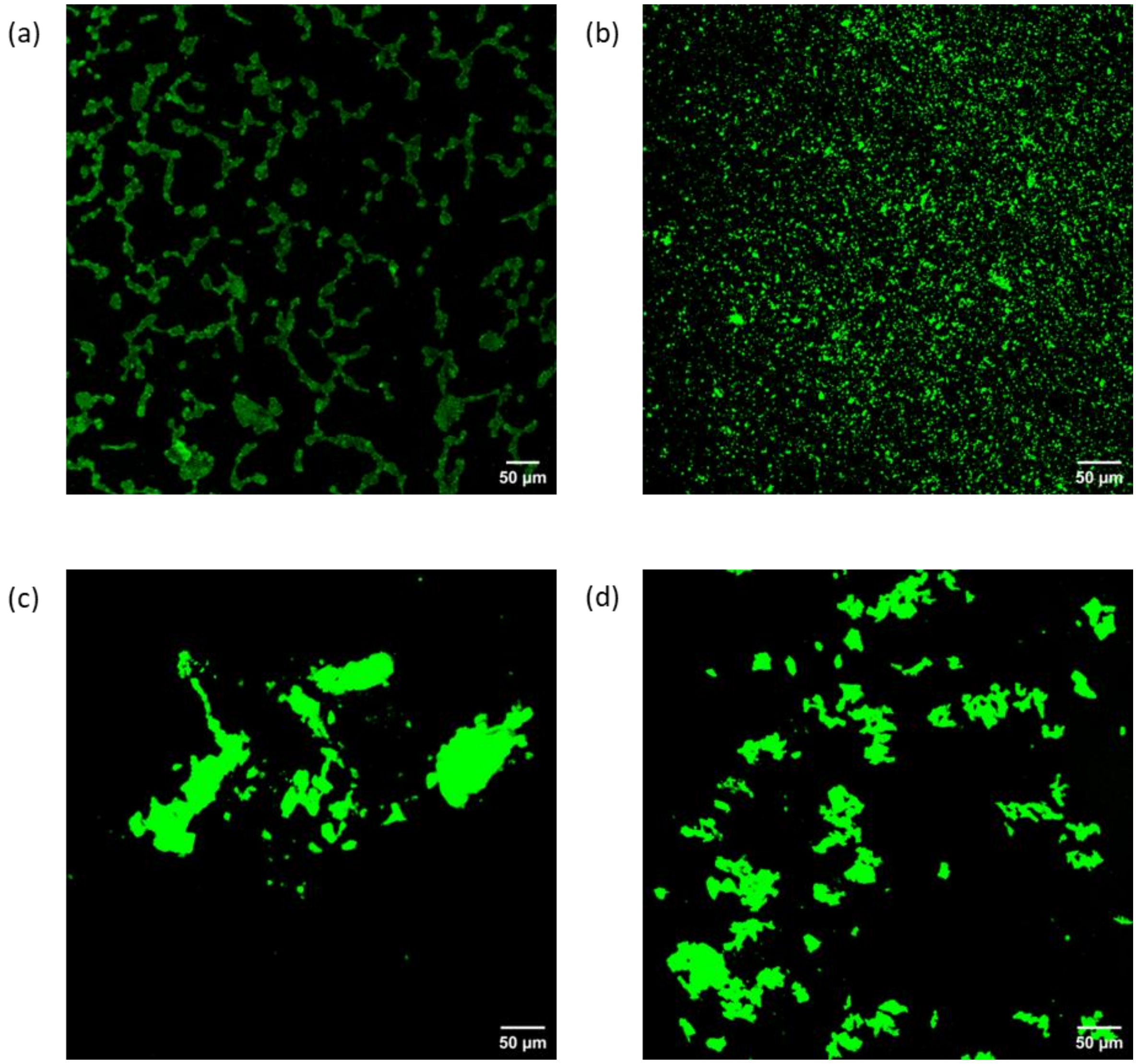

Figure S48: Confocal fluorescence imaging of (a) FITC-BSA@ZnBDC-NH_2 I, (b) FITC-BSA@ZnBDC-NH_2 II, (c) FITC-BSA@ZnBPDC-NH_2 and (d) FITC-BSA@ZnTPDC-NH_2.

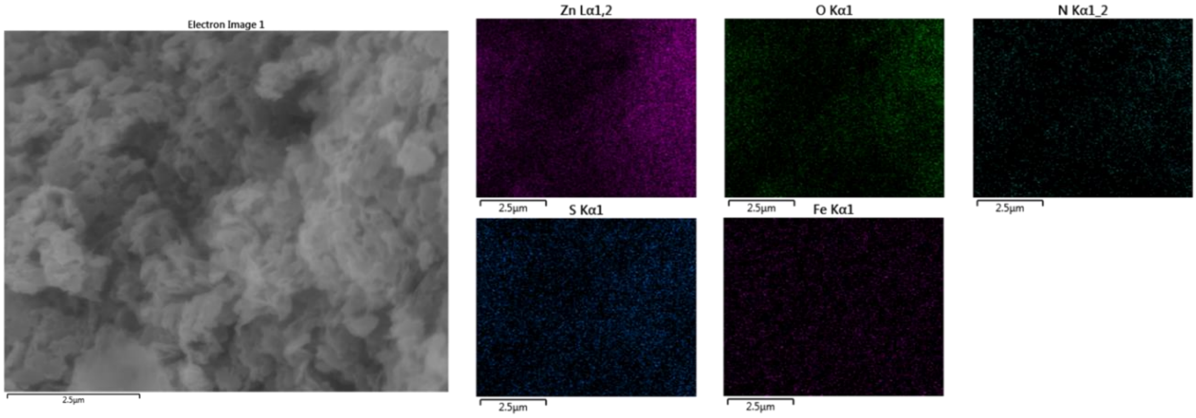

Figure S49: SEM-EDX image of HRP@ZnBDC-NH_2 I and the corresponding elemental mapping of zinc, oxygen, nitrogen, sulphur, and iron.

SEM-EDX Map Sum Spectrum	
**Element**	**Weight %**
Zn	28.6
O	10.0
N	2.2
S	0.5
Fe	0.1

Table S3: SEM-EDX map sum spectrum of HRP@ZnBDC-NH_2 I.

	Elemental Analysis (wt%)	
**Element**	**ZnBDC-NH_2 I**	**HRP@ZnBDC-NH_2 I**
C	34.10	38.17
H	3.23	2.35
N	4.84	5.44
Zn	23.36	23.40
Fe	Not found	0.40

Table S4: Elemental analysis results for ZnBDC-NH_2 I and HRP@ZnBDC-NH_2 I.

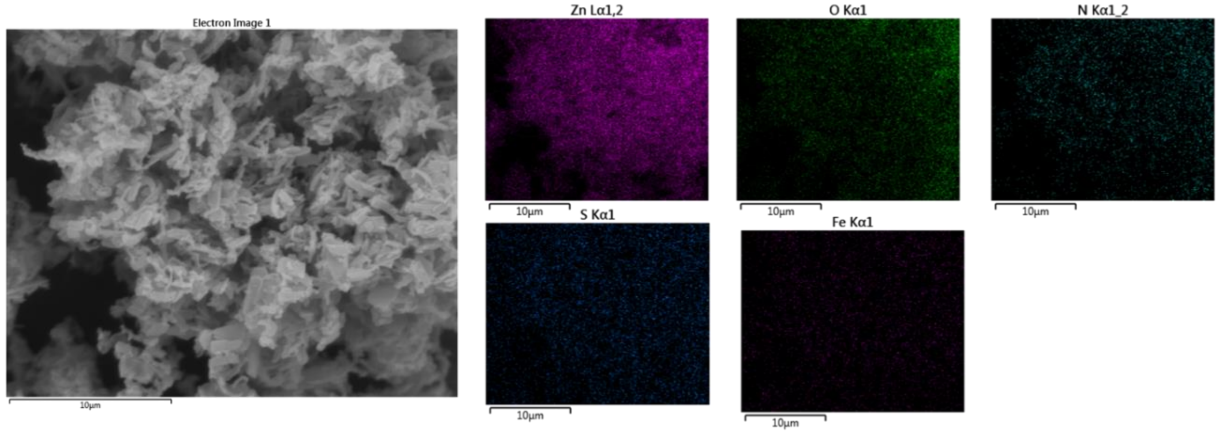

Figure S50: SEM-EDX image of HRP@ZnBDC-NH_2 II and the corresponding elemental mapping of zinc, oxygen, nitrogen, sulphur and iron.

SEM-EDX Map Sum Spectrum	
**Element**	**Weight %**
Zn	30.2
O	15.9
N	6.3
S	0.5
Fe	0.2

Table S5: SEM-EDX Map Sum Spectrum of HRP@ZnBDC-NH_2 II.

	Elemental Analysis (wt%)	
**Element**	**ZnBDC-NH_2 II**	**HRP@ZnBDC-NH_2 II**
C	37.12	34.21
H	2.85	5.08
N	4.89	3.24
Zn	24.53	21.92
Fe	Not found	0.10

Table S6: Elemental analysis data for ZnBDC-NH_2 II and HRP@ZnBDC-NH_2 II.

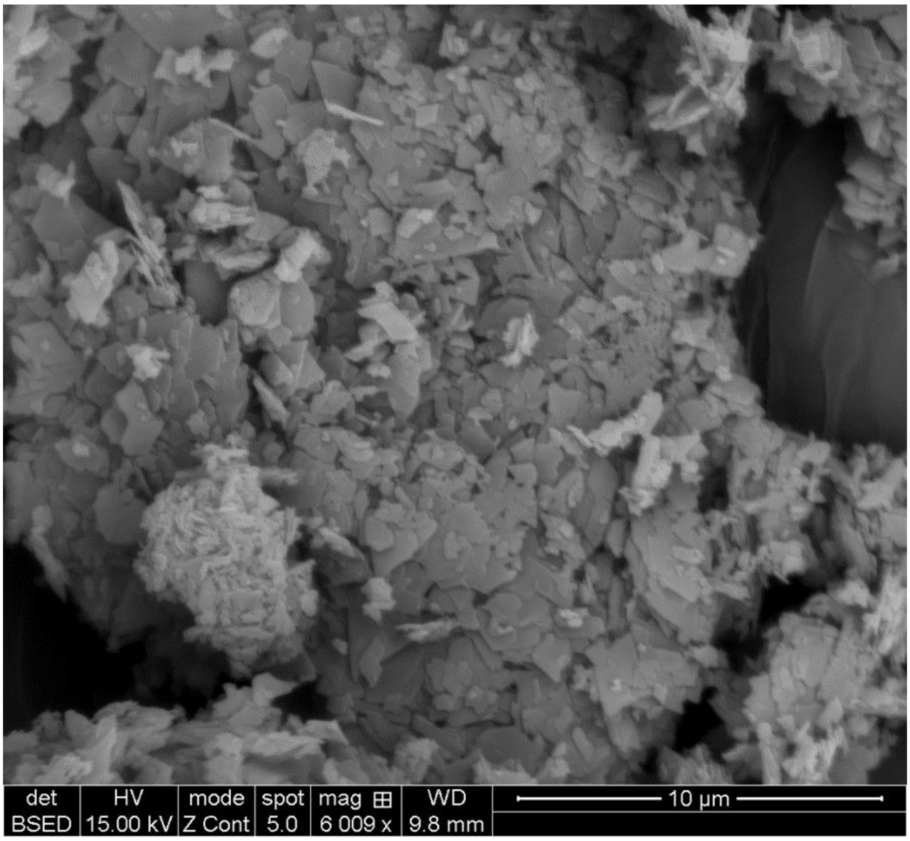

Figure S51: SEM image of ZnBDC-NH_2 I. The sample comprises of irregularly shaped plates which have clustered into aggregates; heterogenous particle sizes are observed for MOF polymorph I and no higher organisation of these particles was observed over extended periods.

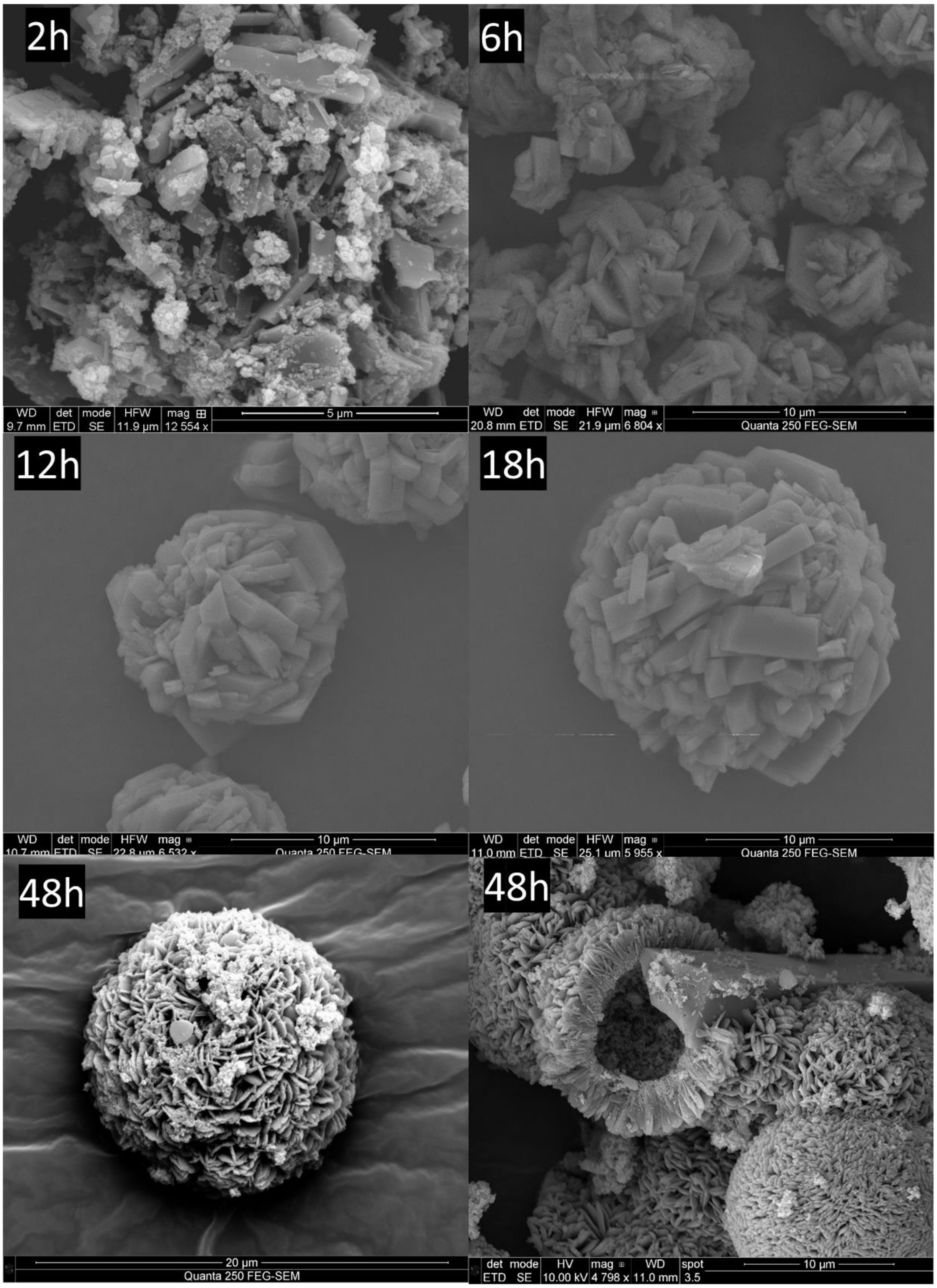

Figure S52: SEM images of HRP@ZnBDC-NH_2 II obtained after different crystallisation times (from 2 hours to 48 hours). Early timepoint images show irregularly shaped plates/ blocks. At increasing time points the plates are shown to self-assemble into spherical aggregates.

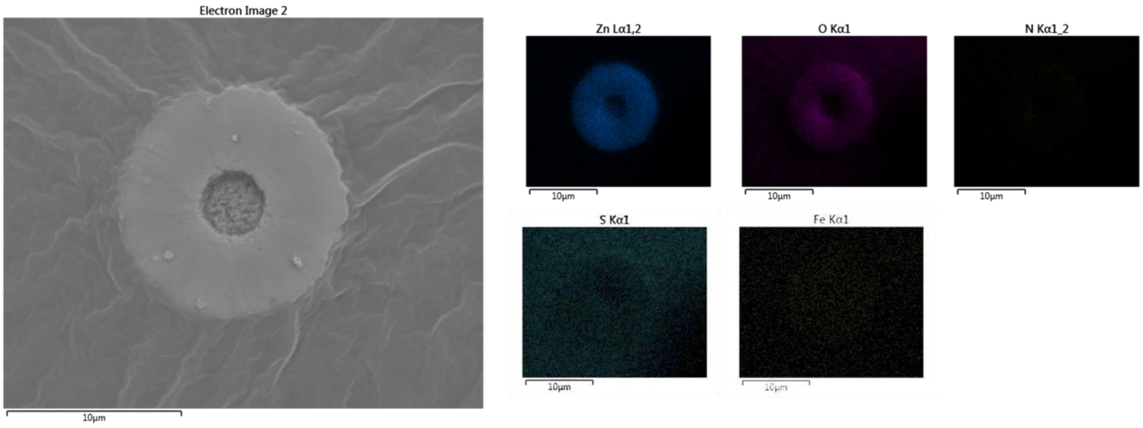

Figure S53: SEM-EDX image of an HRP@ZnBDC-NH_2 II hemisphere and the elemental map of zinc, oxygen, nitrogen, sulphur and iron.

S3.5 Characterisation of ZnBPDC-NH_2 and HRP@ZnBPDC-NH_2

.

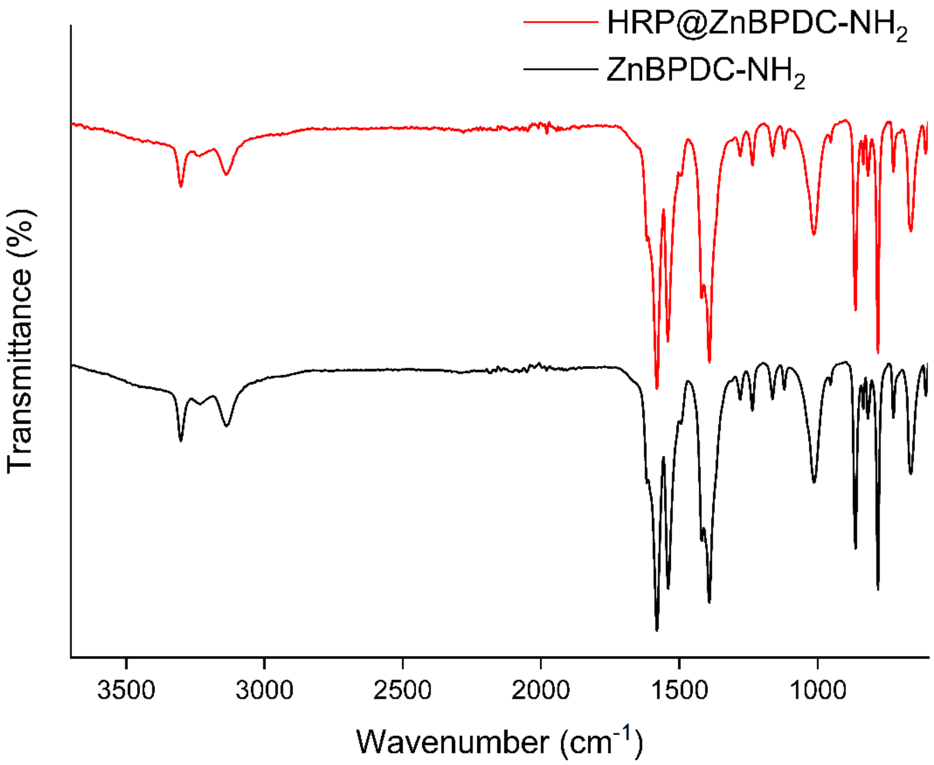

Figure S54: FTIR spectra of HRP@ZnBPDC-NH_2(red) and ZnBPDC-NH_2 (black).

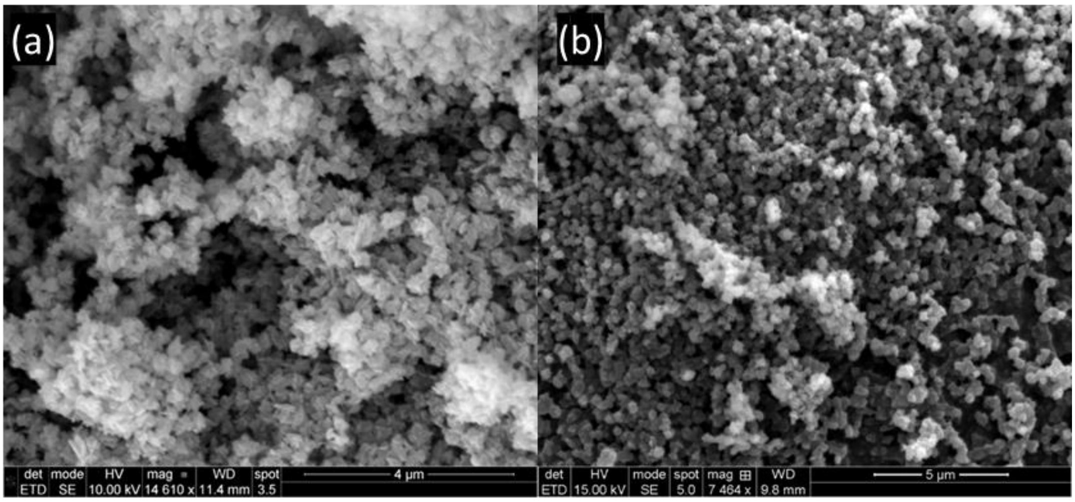

Figure S55: SEM image of a) HRP@ZnBPDC-NH_2 and b) ZnBPDC-NH_2. Particles incorporating HRP are observed as small star-like clusters.

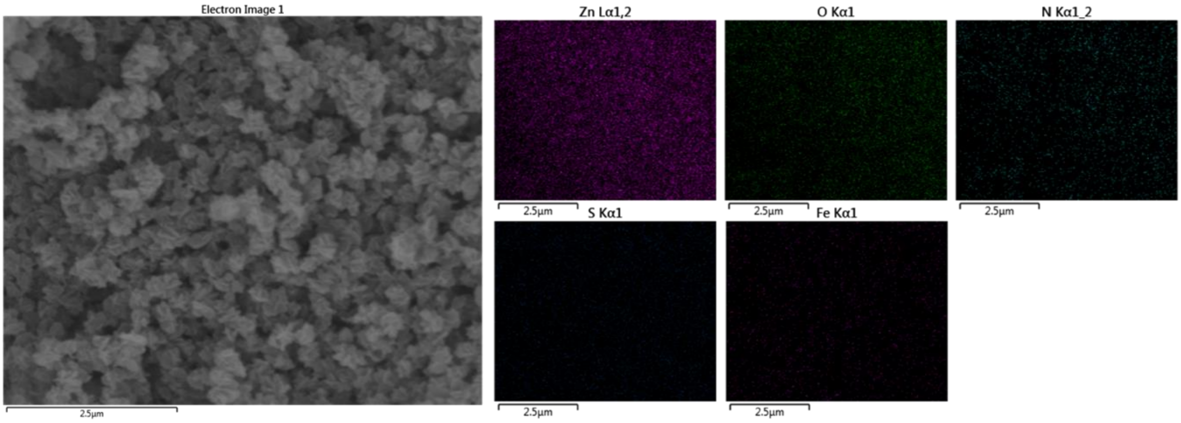

Figure S56: SEM-EDX image of HRP@ZnBPDC-NH_2 and the elemental mapping of zinc, oxygen, nitrogen, sulphur, and iron.

**SEM-EDX Map Sum Spectrum**	
**Element**	**Weight %**
Zn	29.7
O	13.6
N	5.2
S	0.5
Fe	0.3

Table S7: SEM-EDX map sum spectrum of HRP@ZnBPDC-NH_2.

	Elemental Analysis (wt%)	
**Element**	**$ZnBPDC-NH_2$**	**$HRP@ZnBPDC-NH_2$**
C	46.28	46.43
H	3.25	3.07
N	7.63	7.75
Zn	18.28	16.77
Fe	Not found	0.58

Table S8: Elemental analysis data of $ZnBPDC-NH_2$ and $HRP@ZnBPDC-NH_2$

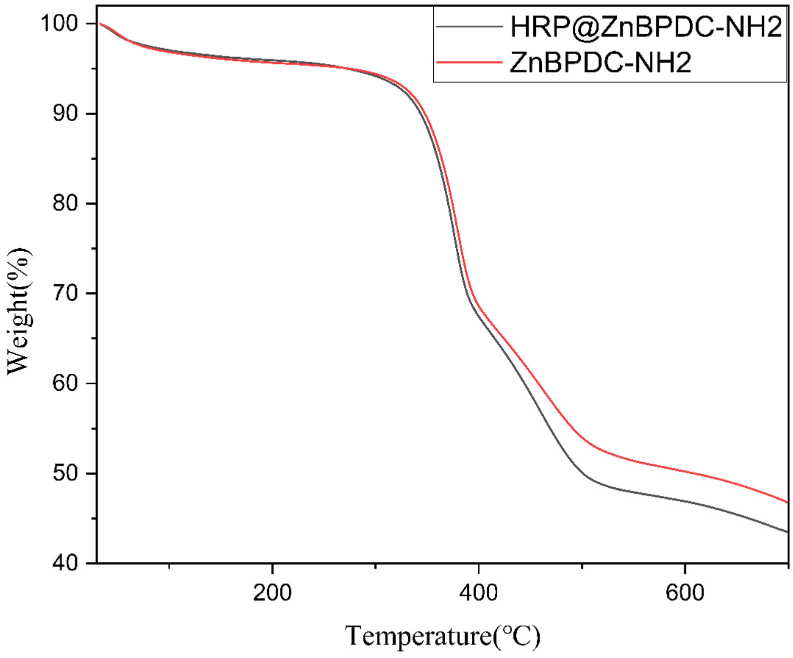

Figure S57: TGA curves of $HRP@ZnBPDC-NH_2$ (black) and $ZnBPDC-NH_2$ (red).

S3.6 Characterisation of $ZnTPDC-NH_2$ and $HRP@ZnTPDC-NH_2$

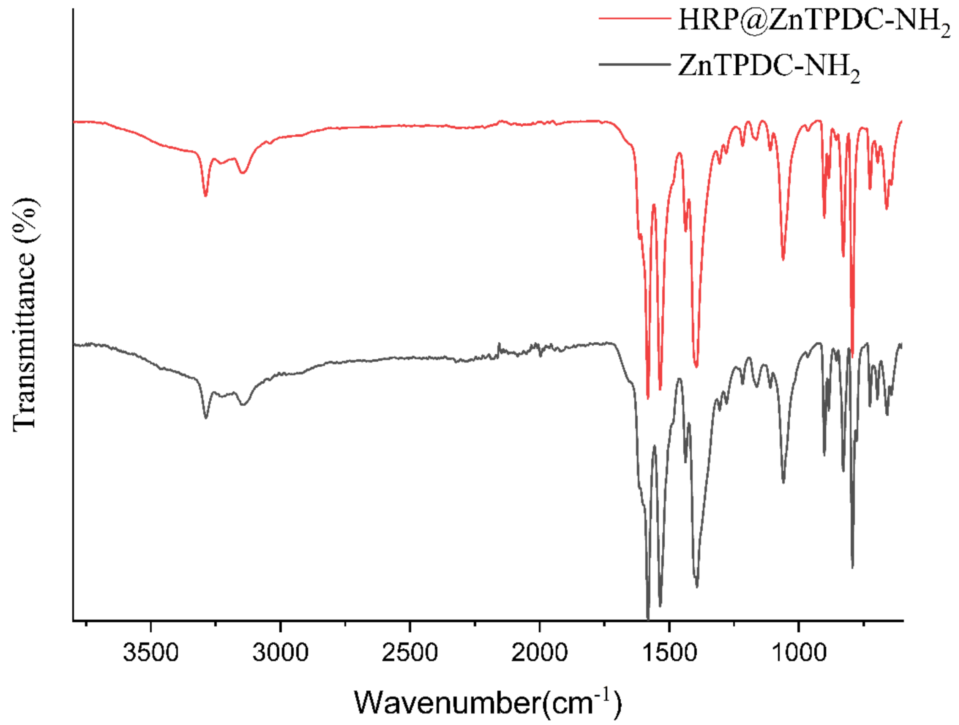

Figure S58: FTIR spectrum of $HRP@ZnTPDC-NH_2$ (red) and $ZnTPDC-NH_2$ (black).

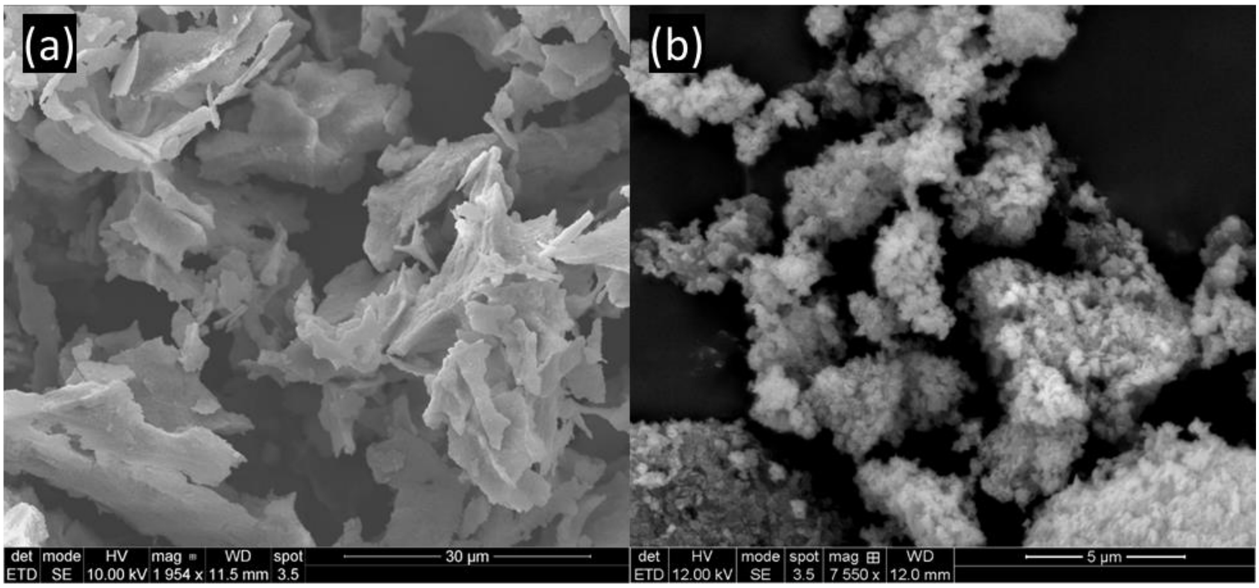

Figure S59: SEM image of $HRP@ZnTPDC-NH_2$ (a) and $ZnTPDC-NH_2$ (b).

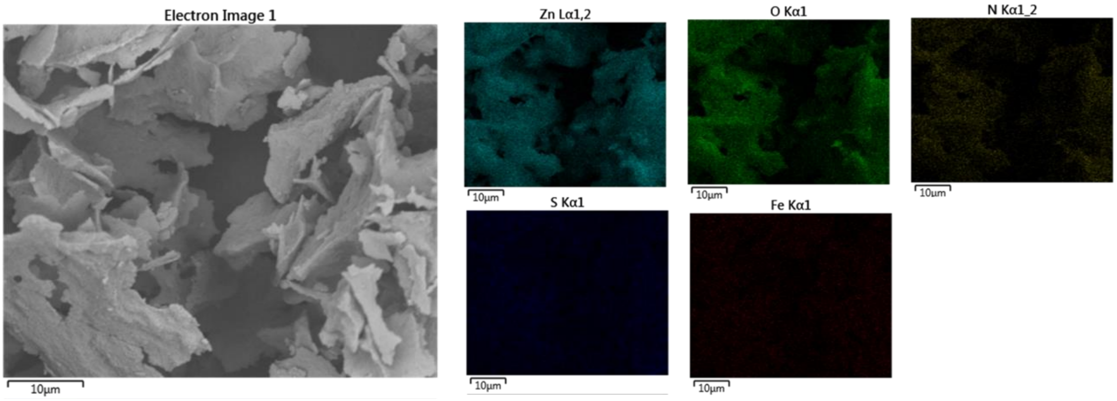

Figure S60: SEM-EDX image of HRP@ZnTPDC-NH_2 and the elemental mapping of zinc, oxygen, nitrogen, sulphur and iron.

**SEM-EDX Map Sum Spectrum**	
**Element**	**Weight %**
Zn	31.21
O	18.72
N	6.46
S	0.67
Fe	0.2

Table S9: SEM-EDX map sum spectrum of HRP@ZnTPDC-NH_2.

	**Elemental Analysis (wt%)**	
**Element**	**ZnTPDC-NH_2**	**HRP@ZnTPDC-NH_2**
C	47.52	37.84
H	3.54	3.24
N	5.63	5.40
Zn	18.83	19.17
Fe	Not found	0.33

Table S10: Elemental analysis data for ZnTPDC-NH_2 and HRP@ZnTPDC-NH_2.

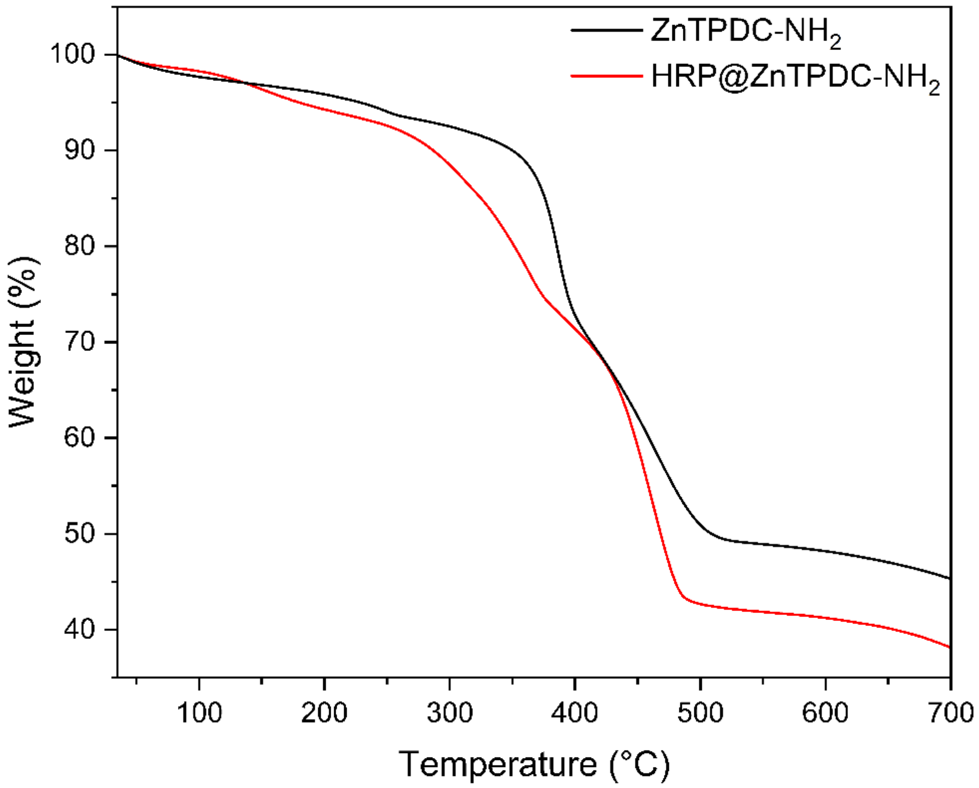

Figure S61: TGA curves of HRP@ZnTPDC-NH_2(red) and ZnTPDC-NH_2(black). A larger mass loss is recorded for the sample containing HRP at a lower temperature, consistent with the results of similar studies in the literature.

S3.7 PXRD Pattern Recorded for HRP@ZnBTB-NH_2

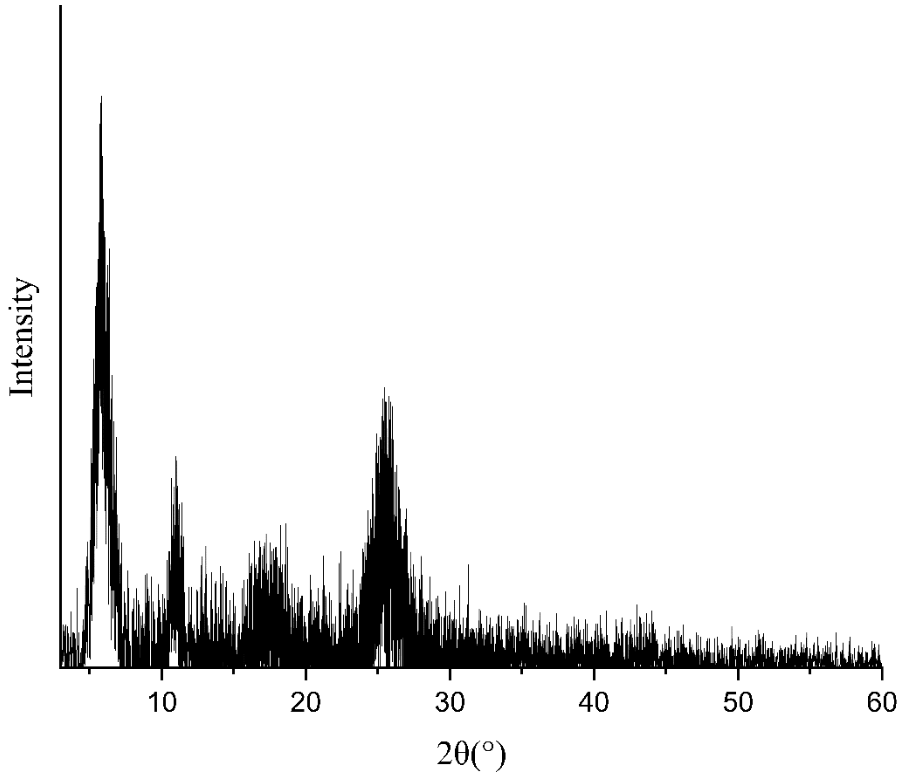

Figure S62: PXRD pattern of HRP@ZnBTB-NH_2. The broadened features indicate a significant reduction in crystallinity compared with the other ligands investigated in this study. As a result, no further analysis of HRP@ZnBTB-NH_2 was undertaken.

S3.8 Comparison of Key Protein@MOF Parameters

Samples	Z-average (nm)	Polydispersity index (PdI)
HRP@ZnBDC-NH_2 I	193.47	0.253
HRP@ZnBDC-NH_2 II	780.95	0.682
HRP@ZnBPDC-NH_2	354.77	0.089
HRP@ZnTPDC-NH_2	354.73	0.648

Table S11: DLS measurement for HRP@ZnBDC-NH_2 I, HRP@ZnBDC-NH_2 II, HRP@ZnBPDC-NH_2 and HRP@ZnTPDC-NH_2.

Catalysts	EE (%)	LC (%)
HRP@ZnBDC-NH_2 I	55.6	1.5
HRP@ZnBDC-NH_2 II	63.7	6.1
HRP@ZnBPDC-NH_2	86.1	1.0
HRP@ZnTPDC-NH_2	24.5	0.1

Table S12: Encapsulation efficiency (EE) and loading content (LC) of HRP@ZnBDC-NH_2 I, HRP@ZnBDC-NH_2 II, HRP@ZnBPDC-NH_2 and HRP@ZnTPDC-NH_2 calculated based on the amount of protein remaining in the supernatant following biomimetic crystallisation, as determined from the Bradford assay.

S4 Kinetic Analysis

S4.1 General Kinetics Protocol

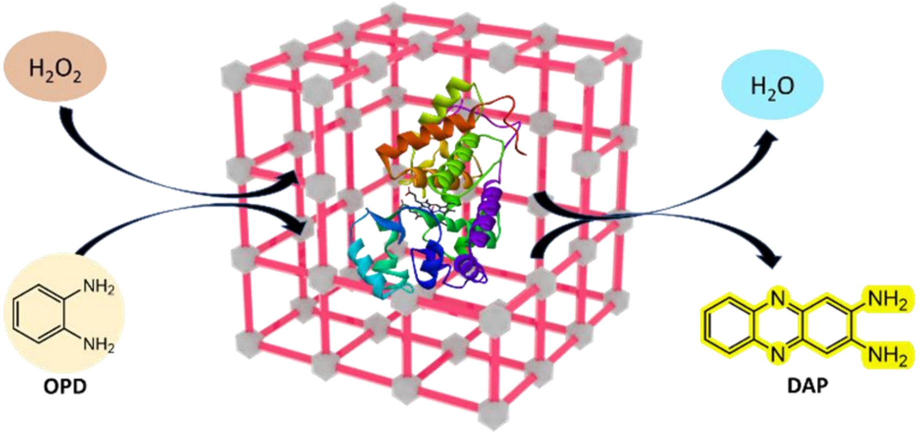

Scheme S4: Schematic illustration of the OPD (*o*-phenylenediamine) assay catalysed by HRP@MOFs. Reaction progress is followed by observation of the DAP (2,3-diaminophenazine) signal at 417 nm.

The catalytic activity of the HRP@MOFs synthesised in this study was determined using a modified OPD assay adapted for use in 96-well plates.[9]

Stable stock suspensions of each of the HRP@MOFs were prepared by dispersing HRP@MOFs (1.0 mg) in DI water (1.0 mL) with ultrasonic dispersion. Based on the HRP Loading Content (Table S11), the appropriate volume of stock solution was transferred to each reaction well ensuring that the relative concentration of HRP in the working solution was standardised to ~50 ng/mL (1.25 nM). The concentration of substrate OPD was varied from 100 µM to 2000 µM per reaction well, the final volume in each well was made up to 300 µL with DI water. The reaction was initiated upon the addition of 1 µL hydrogen peroxide (8.9 mM) to each well and the absorbance at 417 nm was recorded at 30 second intervals for 120 minutes.

Background correction: Background absorbance attributed to scattering from the MOF particles and slow aerial oxidation of OPD was removed. Fresh background measurements were recorded for each experiment from solutions of HRP@MOF in the absence of the OPD and H_2O_2, as well as an OPD and H_2O_2 solution in the absence of HRP@MOF.

Following background correction, key Michaelis-Menten parameters (Km and Vmax) were extracted using established processing procedures.

$$E+S \underset{k_r}{\overset{k_f}{\rightleftharpoons}} ES \xrightarrow{k_{cat}} E+P$$

$$v = \frac{d[P]}{dt} = \frac{Vm[S]}{Km + [S]}$$

Equation 3: Michaelis -Menten equation and the derivation of K_m and V_m.

S4.2 Kinetic Data for HRP@MOF Samples

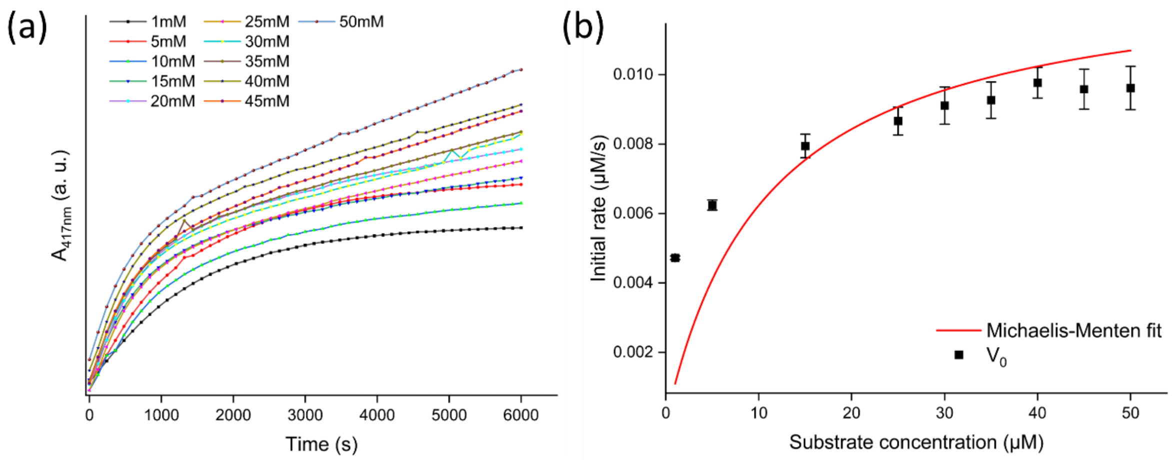

Figure S63: (a) Absorbance versus time plots following the OPD assay with HRP as a catalyst. (b) Michaelis-Menten nonlinear fitting of the V_0 to substrate concentration curve.

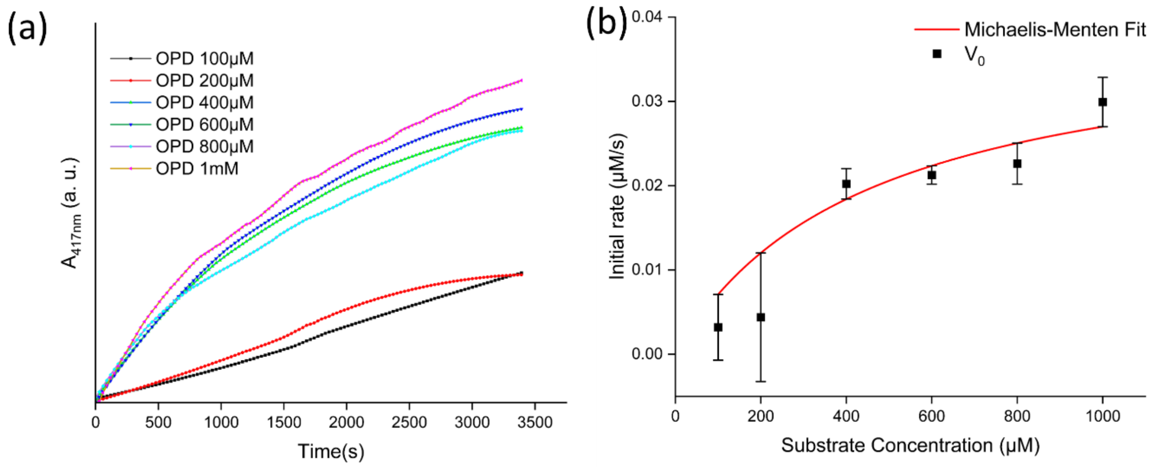

Figure S64: (a) Absorbance versus time plots following the OPD assay with HRP@ZnBDC-NH_2 I as the catalyst. (b) Michaelis-Menten nonlinear fitting of the V_0 to substrate concentration curve.

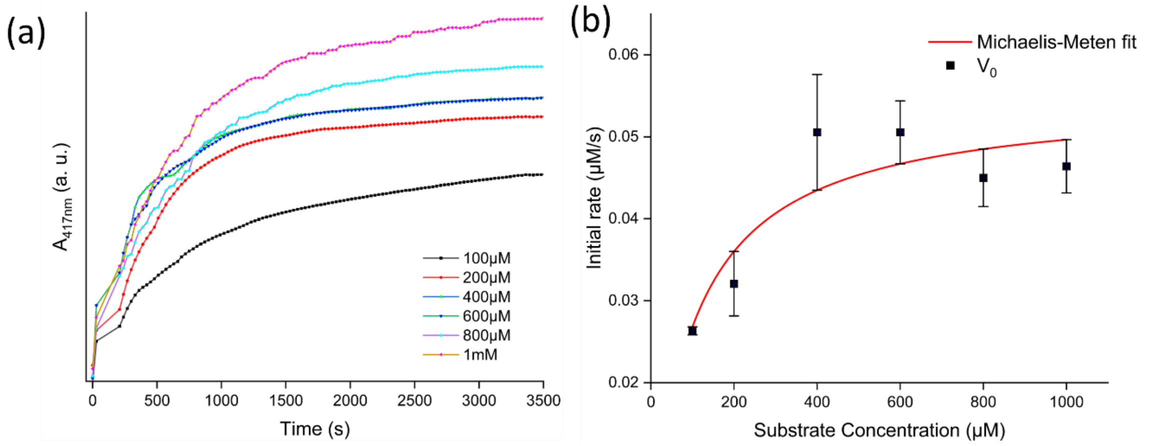

Figure S65: (a) Absorbance versus time plots following the OPD assay with HRP@ZnBDC-NH_2 II as the catalyst. (b) Michaelis-Menten nonlinear fitting of the V_0 to substrate concentration curve.

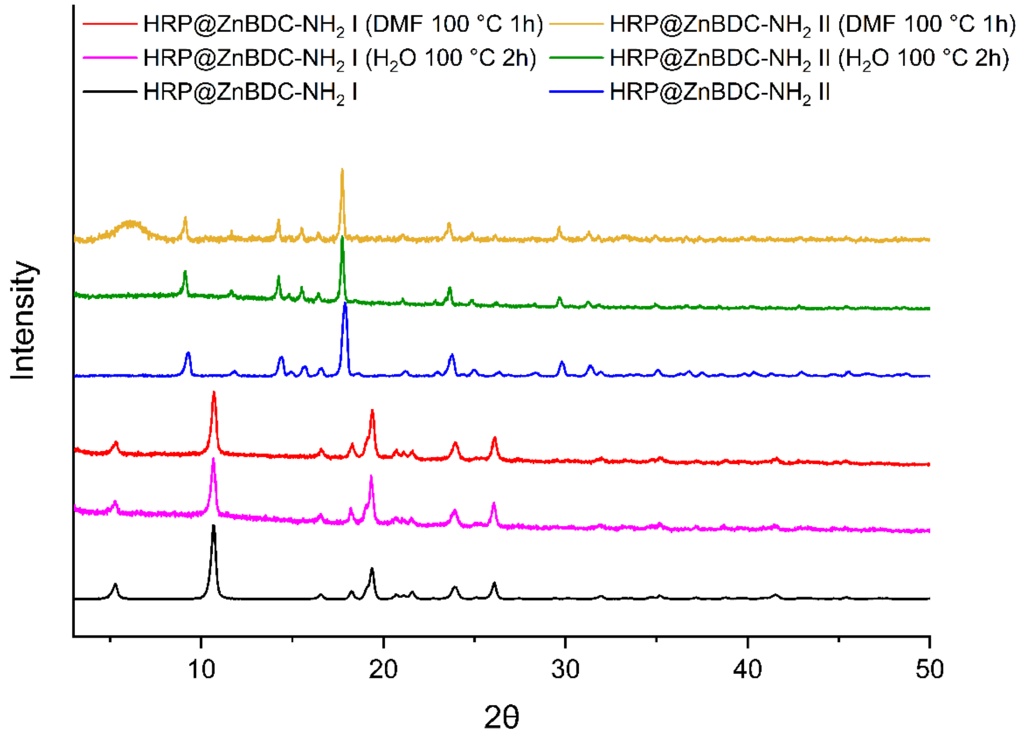

Figure S66: PXRD pattern of HRP@ZnBDC-NH_2 I and HRP@ZnBDC-NH_2 II recorded after different thermal treatments.

S5 References

1. Luo, T.-Y., et al., *Rare earth pcu metal–organic framework platform based on $RE_4(\mu_3\text{-}OH)_4(COO)_6^{2+}$ clusters: rational design, directed synthesis, and deliberate tuning of excitation wavelengths.* J. Am. Chem. Soc., 2017. **139**(27), 9333-9340.
2. Zhang, Y.-B., et al., *Introduction of functionality, selection of topology, and enhancement of gas adsorption in multivariate metal–organic framework-177.* J. Am. Chem. Soc., 2015. **137**(7), 2641-2650.
3. Coelho, A.A., *TOPAS and TOPAS-Academic: an optimization program integrating computer algebra and crystallographic objects written in C++.* J. Appl. Crystallogr., 2018. **51**(1), 210-218.
4. Groom, C.R., et al., *The Cambridge structural database.* Acta Cryst. B, 2016. **72**(2), 171-179.
5. Petříček, V., et al., *Jana2020–a new version of the crystallographic computing system Jana.* Z. Kristallogr. Cryst. Mater., 2023. **238**(7-8), 271-282.
6. Macrae, C.F., et al., *Mercury 4.0: From visualization to analysis, design and prediction.* J. Appl. Crystallogr, 2020. **53**(1),226-235.
7. Thermoscientific. *Protein assay technical handbook : Tools and reagents for improved quantitation of total or specific proteins*. 2017; Available from: https://assets.thermofisher.com/TFS-Assets/LSG/brochures/protein-assay-technical-handook.pdf.
8. Miao, Y.-B., Q. Zhong, and H.-X. Ren, *Engineering a thermostable biosensor based on biomimetic mineralization HRP@ Fe-MOF for Alzheimer's disease.* Anal. Bioanal. Chem, 2022, 1-9.
9. Thermoscientific. *User Guide: OPD ASSAY*. 2011; Available from: https://www.thermofisher.com/document-connect/document-connect.html?url=https://assets.thermofisher.com/TFS-Assets%2FLSG%2Fmanuals%2FMAN0011188_OPD_UG.pdf.
